# Epidemiological and Evolutionary Dynamics of Influenza B Viruses in Malaysia, 2012-2014

**DOI:** 10.1371/journal.pone.0136254

**Published:** 2015-08-27

**Authors:** Xiang Yong Oong, Kim Tien Ng, Tommy Tsan-Yuk Lam, Yong Kek Pang, Kok Gan Chan, Nik Sherina Hanafi, Adeeba Kamarulzaman, Kok Keng Tee

**Affiliations:** 1 Department of Medicine, Faculty of Medicine, University of Malaya, Kuala Lumpur, Malaysia; 2 School of Public Health, The University of Hong Kong, Hong Kong Special Administrative Region, Hong Kong, China; 3 Division of Genetics and Molecular Biology, Institute of Biological Sciences, Faculty of Science, University of Malaya, Kuala Lumpur, Malaysia; 4 Department of Primary Care Medicine, Faculty of Medicine, University of Malaya, Kuala Lumpur, Malaysia; Icahn School of Medicine at Mount Sinai, UNITED STATES

## Abstract

Epidemiological and evolutionary dynamics of influenza B Victoria and Yamagata lineages remained poorly understood in the tropical Southeast Asia region, despite causing seasonal outbreaks worldwide. From 2012–2014, nasopharyngeal swab samples collected from outpatients experiencing acute upper respiratory tract infection symptoms in Kuala Lumpur, Malaysia, were screened for influenza viruses using a multiplex RT-PCR assay. Among 2,010/3,935 (51.1%) patients infected with at least one respiratory virus, 287 (14.3%) and 183 (9.1%) samples were tested positive for influenza A and B viruses, respectively. Influenza-positive cases correlate significantly with meteorological factors—total amount of rainfall, relative humidity, number of rain days, ground temperature and particulate matter (PM10). Phylogenetic reconstruction of haemagglutinin (HA) gene from 168 influenza B viruses grouped them into Yamagata Clade 3 (65, 38.7%), Yamagata Clade 2 (48, 28.6%) and Victoria Clade 1 (55, 32.7%). With neuraminidase (NA) phylogeny, 30 intra-clade (29 within Yamagata Clade 3, 1 within Victoria Clade 1) and 1 inter-clade (Yamagata Clade 2-HA/Yamagata Clade 3-NA) reassortants were identified. Study of virus temporal dynamics revealed a lineage shift from Victoria to Yamagata (2012–2013), and a clade shift from Yamagata Clade 2 to Clade 3 (2013–2014). Yamagata Clade 3 predominating in 2014 consisted of intra-clade reassortants that were closely related to a recent WHO vaccine candidate strain (B/Phuket/3073/2013), with the reassortment event occurred approximately 2 years ago based on Bayesian molecular clock estimation. Malaysian Victoria Clade 1 viruses carried H274Y substitution in the active site of neuraminidase, which confers resistance to oseltamivir. Statistical analyses on clinical and demographic data showed Yamagata-infected patients were older and more likely to experience headache while Victoria-infected patients were more likely to experience nasal congestion and sore throat. This study describes the evolution of influenza B viruses in Malaysia and highlights the importance of continuous surveillance for better vaccination policy in this region.

## Introduction

Influenza A and B viruses are important pathogens to humans, contributing to a large proportion of morbidity and mortality in respiratory infections worldwide [1]. Both viruses have a segmented negative-stranded genome with an enveloped structure [2]. Due to the limited host range (with no natural animal hosts other than humans and seals) of influenza B viruses as compared to influenza A viruses [3], antigenic shift does not occur in influenza B viruses, and hence they have little pandemic potential [4]. However, influenza B viruses evolve through antigenic drift, enabling them to escape host immunity and continue to adapt to new environments [5], causing significant disease burden to the global population [6]. Two major genetically and antigenically distinct influenza B lineages, B/Victoria/2/87-like (Victoria lineage) and B/Yamagata/16/88-like (Yamagata lineage) have been detected since 1983 [7, 8]. However, in recent decades, the changing demographics and rapid movement of human populations between countries [9] have resulted in the co-circulation and recurring outbreaks of two influenza B lineages in many regions of the world [6, 10–19].

Studies have shown that the tropical East and Southeast Asia region played a role in reseeding new genetic variants of influenza A (H3N2) viruses in the temperate region, causing an annual H3N2 epidemic worldwide [20, 21]. In contrast, recent phylogeographic analyses from 2000 to 2012 revealed that influenza B Victoria and Yamagata lineage viruses within East and Southeast Asia region commonly circulate exclusively within this region for several years with limited spreading to other regions [22]. Whether this region continues to play a role in global influenza B epidemics still requires further investigation as recent dominant strains isolated in several countries in both Northern and Southern hemispheres during the 2014/2015 season were closely related to strains with a Southeast Asia origin [23, 24].

A recent report on the evolution and epidemiology of influenza B virus in Malaysia form 1995–2008, which generally involved the children population, described the incidence and evolutionary changes of predominant circulating Victoria and Yamagata lineages over the years [25]. In view of this, we had conducted an influenza surveillance on more adult and elderly patients with respiratory infections and investigated the epidemiological and evolutionary dynamics of influenza B lineages circulating in Malaysia between 2012 and 2014 using phylogenetic methods. Clinical presentation and demographic profile of patients infected by both lineages were compared. Although studies in temperate regions have highlighted the role of humidity and temperature in shaping influenza seasonality [26–28], such studies were lacking in the tropical Southeast Asia region. Hence, we also aimed to identify possible meteorological predictors that drive the seasonal periodicity of influenza viruses by associating climatic variables to influenza activities.

## Materials and Methods

### Ethical Statement

This study was approved by the University of Malaya Medical Centre (UMMC) Medical Ethics Committee (MEC890.1). Standard, multilingual consent forms validated by the Medical Committee were used. Written consent was obtained from all study participants.

### Clinical Specimen Collections

A total of 3,935 nasopharyngeal swab samples were collected from outpatients experiencing symptoms of acute upper respiratory tract infection at the Primary Care Clinic of UMMC in Kuala Lumpur, Malaysia between February 2012 and May 2014. The presence of symptoms associated with acute respiratory tract infection (sneezing, nasal discharge, nasal obstruction, headache, sore throat, hoarseness of voice, muscle ache and cough) was self-recorded by the patient on a questionnaire, which was designed based on previously reported criteria [29]. The nasopharyngeal samples were then transported in universal transport medium to the laboratory and stored at -80°C before further processing.

### Rapid Detection, Amplification and Sequencing of Influenza B Viruses

Total nucleic acids from nasopharyngeal samples were extracted using the NucliSENS easyMAG automated nucleic acid extraction system (BioMérieux, France) [30, 31]. Influenza B viruses and other respiratory viruses (including influenza A viruses) were then detected using the xTAG Respiratory Virus Panel (RVP) *FAST* multiplex RT-PCR assay (Luminex Molecular Diagnostics, USA) [32, 33]. Both extraction and detection procedures were performed based on manufacturer’s protocol.

A two-step reverse transcription PCR (RT-PCR) approach was adopted following protocols recommended by the World Health Organization (WHO) on influenza B-positive samples [34]. The HA and NA genes were amplified as overlapping halves using WHO-recommended gene specific primers (primer sequences and cycling conditions are listed in S1 Table). PCR products were purified and sequencing was performed in an ABI PRISM 3730XL Genetic Analyzer using the BigDye Terminator v3.1 cycle sequencing kit chemistry (Applied Biosystems, USA). The HA and NA sequence reads were assembled into a contig and aligned with the WHO vaccine and reference sequences.

### Phylodynamic Analysis of the HA and NA Genes

Evolutionary analyses of HA and NA gene sequences were carried out on all Malaysian influenza B viruses and other related virus strains worldwide. Our sequence data set included those from the Global Initiative on Sharing all Influenza Data (GISAID) [35] and GenBank within the study period. WHO recommended vaccine and reference strains were also included [23, 24]. All gene sequences were edited and aligned in the BioEdit 7.2 [36]. Phylogenetic trees of the HA and NA genes were reconstructed using maximum likelihood (ML) method heuristically inferred using subtree pruning and regrafting and nearest neighbor interchange algorithms with general time-reversible (GTR) nucleotide substitution model, a proportion of invariant sites (+I) and four categories of gamma rate heterogeneity (+Γ_4_), implemented in PAUP version 4.0 [37]. Robustness of the branching orders was evaluated by bootstrap analysis of 1,000 replicates.

Molecular clock dating analysis using the Bayesian Markov chain Monte Carlo (MCMC) method implemented in BEAST 1.7 [38] was used to estimate the timescale for the emergence of the B/Phuket/3073/2013-like viruses detected in the region, as previously described [14, 39–41]. The uncorrelated lognormal relaxed clock model with GTR+I+Γ_4_ nucleotide substitution model and Bayesian skyline plot (BSP) model was employed to estimate the posterior distribution of phylogenies, nucleotide substitution rates, and the time of most recent common ancestor (tMRCA) of B/Phuket/3073/2013-like viruses. Three independent MCMC runs of 50 million steps sampled for every 50,000 states were performed on HA and NA genes separately. The MCMC sampling was assessed for convergence (effective sampling size > 200) after 10% burn-in using Tracer 1.4 (http://tree.bio.ed.ac.uk). Bayesian maximum clade credibility (MCC) trees were annotated using TreeAnnotator in BEAST package and visualized in FigTree (http://tree.bio.ed.ac.uk/software/figtree/) to determine the posterior probability of B/Phuket/3073/2013-like clade.

### Statistical Analysis on Meteorological, Demographical and Clinical Data

To understand the seasonality of influenza viruses in Malaysia, meteorological data were obtained from the Malaysian Meteorological Department (Kuala Lumpur) (03°06'N, 101°39'E). Meteorological factors including daily rainfall amount, number of rainy days, relative humidity, ground temperature and particulate matter with size less than 10 micrometers in diameter (PM10) were collected between February 2012 and May 2014. The associations between these data and influenza-positive cases were determined through linear correlation (bivariate and partial) and regression analysis using the Statistical Package for Social Sciences version 22.0 (SPSS Inc., Chicago, USA). Demographic and clinical features between patients infected with influenza B Victoria lineage and Yamagata lineage were also compared using independent samples *t*-test. Association between symptoms and lineages was accessed using Pearson’s chi-square or Fischer’s exact test and binary logistic regression [42].

### Nucleotide Sequence Accession Numbers

HA and NA nucleotide sequences of the Malaysian influenza B viruses generated in this study are available in GenBank under accession numbers KR073326-KR073659.

## Results

### Seasonality of Influenza Viruses in Malaysia

**Fig 1:**
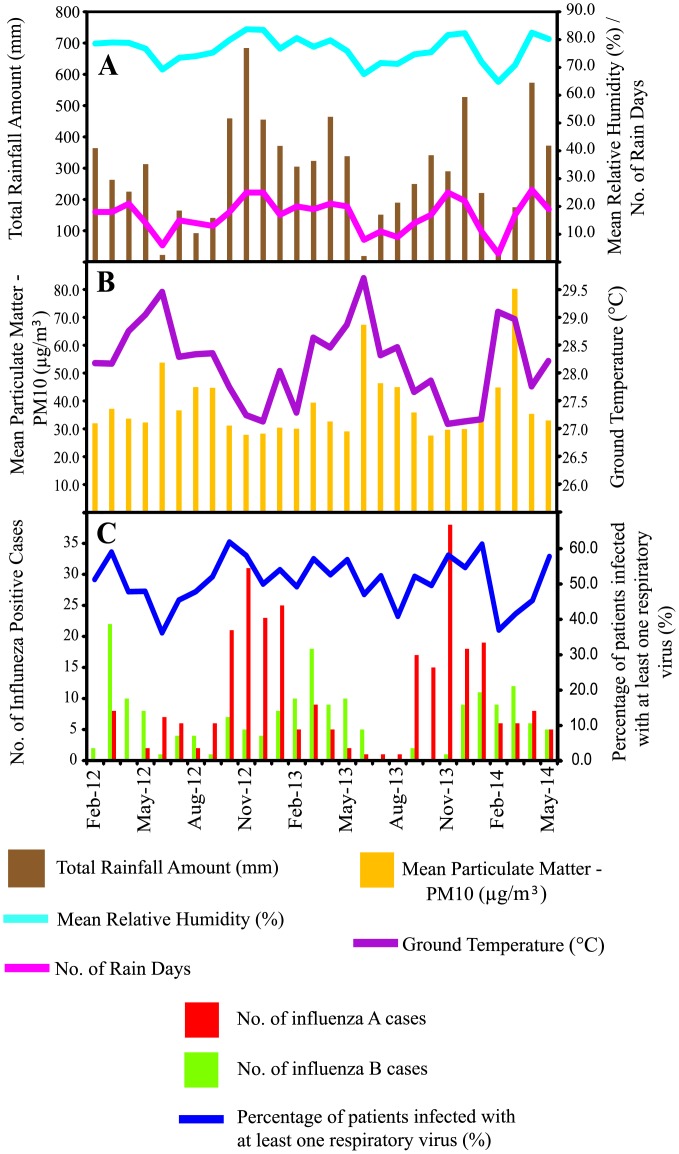
Seasonality of influenza infections and meteorological factors in Malaysia between 2012 and 2014. (A) Total Rainfall Amount (mm), Mean Relative Humidity (%) and Number of Rain Days. (B) Mean Particulate Matter—PM10 (μg/m^3^) and Ground Temperature (°C). (C) Monthly distribution of influenza A and B infections in Kuala Lumpur, Malaysia.

During the study period from February 2012 to May 2014, a total of 287 (14.3%) and 183 (9.1%) samples tested positive for influenza A and B viruses, respectively among 2,010/3,935 (51.1%) patients infected with at least one respiratory virus. We found that the seasonality of influenza viruses showed similar patterns with meteorological factors such as the total amount of rainfall, relative humidity and number of rain days, such that peaks were observed between October to May, and hit their lowest marks around June to September in 2012 and 2013 (Fig 1A and 1C). However, both particulate matter (PM10) and ground temperature showed opposite trends as influenza activity was reduced during an increase in particular matter and ground temperature (Fig 1B and 1C).

The bivariate correlation between three meteorological factors (total amount of rainfall, relative humidity and number of rain days) and number of influenza cases were significantly positive (*p* < 0.05), while both particulate matter and ground temperature showed significant negative correlation (*p* < 0.05) with number of influenza cases (Table 1). Though, partial correlation showed that only ground temperature was significantly associated with the number of influenza cases (*p* < 0.05). A multiple linear regression analysis showed that the linear combination of all five meteorological factors (non-ordered predictors) was significantly related to the number of influenza cases (outcome) (*R* ^2^ = 0.458, adjusted *R* ^2^ = 0.329, *F* (5, 21) = 3.549, *p* < 0.05). Based on both linear correlational and regression analyses, ground temperature is perhaps the main predictor (standard regression coefficient, beta = -0.545, t (26) = -2.308, *p* < 0.05) which accounts for 40.5% (*r*: -0.636, *R* ^2^ = 0.405) of the variance of the number of influenza cases, while the other variables contributed only an additional 5.3% (45.8%- 40.5% = 5.3%). However, such judgments about the relative importance of these predictors are challenging because they are also correlated among themselves (range of *r*: 0.407–0.931, *p* < 0.05, data not shown).

**Table 1:** Linear correlations and regression between meteorological factors and number of influenza positive cases (March 2012 –May 2014).

		Bivariate Correlations(Correlation between each predictors and the no. of positive cases)		Partial Correlations(Correlation between each predictor and the no. of positive cases controlling for all other predictors)		Standardized regression coefficients	*t*
Meteorological Factors (Predictors)	Mean (± S.D.)	*r*	*p*	*r*	*p*	*Beta*	*t*
Total Rainfall Amount (mm)	287.867 (± 168.141)	0.545	0.003*	0.176	0.423	0.265	0.817
Relative Humidity (%)	76.259 (±5.015)	0.518	0.006*	-0.143	0.514	-0.391	-0.664
No. of Rain Days	16.590 (±5.995)	0.520	0.005*	0.154	0.482	0.351	0.716
Particulate Matter (μg/m^3^)	38.551 (±12.352)	-0.407	0.035*	0.029	0.896	0.034	0.132
Ground Temperature (°C)	28.200 (±0.747)	-0.636	<0.001*	-0.450	0.031*	-0.545	-2.308

*S*.*D*.: *standard deviation; r*: Pearson correlation coefficient (high correlation: 0.5 to 1.0 or -0.5 to -1.0; moderate correlation: 0.3 to 0.5 or -0.3 to -0.5); *p*: level of significance (2-tailed);

* correlation is significant at the 0.05 level.

Notable characteristic waves of influenza viruses were also observed (Fig 1C): First, influenza A cases consistently peaked ahead of influenza B and fell between September and January, whereas influenza B cases peaked later between February and April when influenza A cases decreased. Second, the overall prevalence of influenza B infection was consistently lower than that of influenza A virus.

### Phylogenetic Classification of Influenza B Viruses

A total of 168 full-length HA and 166 full-length NA gene sequences were obtained from 170 patients in the present study (S2 Table). Additional 23 Malaysian influenza B viruses with full-length HA and NA gene sequences and collection dates from January 2012 to June 2014 were also retrieved from GISAID and GenBank databases. These published sequences were originated from the National Influenza Centre at the Institute of Medical Research (IMR) Malaysia. Hence, a total of 193 Malaysian influenza B viruses were included for phylogenetic classification.

**Fig 2:**
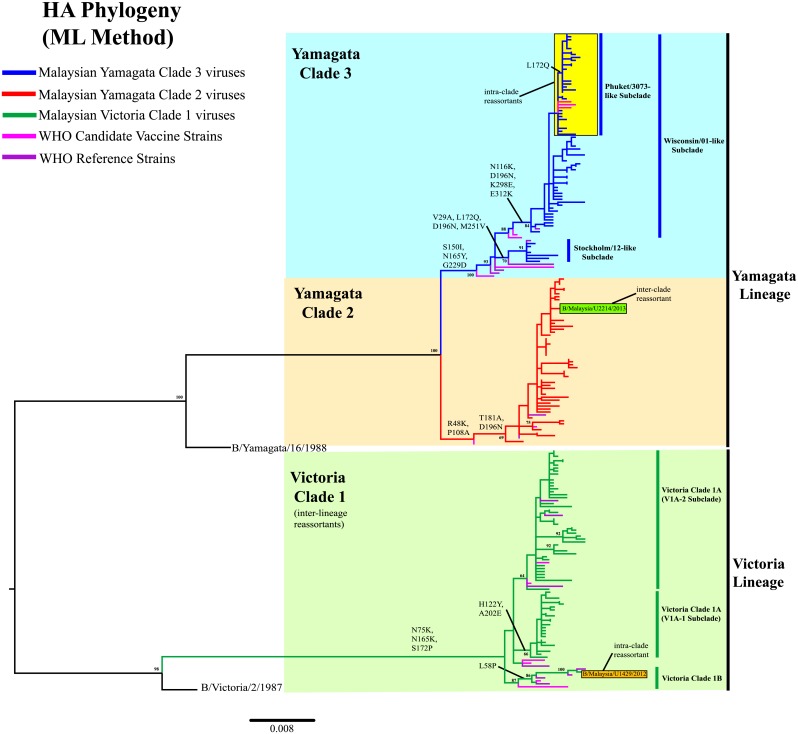
Phylogenetic analysis of the HA gene of influenza B viruses in Malaysia from 2012 to 2014. HA sequences of Malaysian influenza B viruses were compared with WHO recommended candidate vaccine and reference strains. The phylogeny was reconstructed using maximum likelihood (ML) method. Bootstrap values (>60%) and amino acid substitutions are mapped to key branches. Intra-and inter-clade reassortants are indicated as boxes. Yamagata Clade 3 (Yam-3) (blue), Yamagata Clade 2 (Yam-2) (orange) and Victoria Clade 1 (Vic-1) (green) are indicated. Scale bar represents a genetic distance of 0.008 substitutions/site. Phylogenetic tree for HA gene with complete taxa identity is shown in S1 Fig.

**Fig 3:**
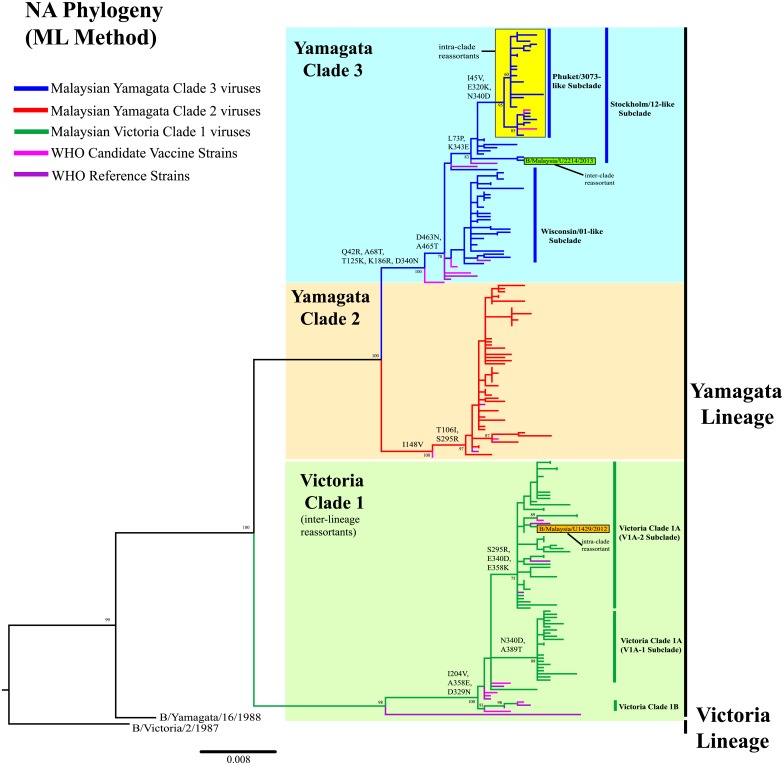
Phylogenetic analysis of the NA gene of influenza B viruses in Malaysia from 2012 to 2014. NA sequences of Malaysian influenza B viruses were compared with WHO recommended candidate vaccine and reference strains. The phylogeny was reconstructed using maximum likelihood (ML) method. Bootstrap values (>60%) and amino acid substitutions are mapped to key branches. Intra-and inter-clade reassortants are indicated as boxes. Yamagata Clade 3 (Yam-3) (blue), Yamagata Clade 2 (Yam-2) (orange) and Victoria Clade 1 (Vic-1) (green) are indicated. Scale bar represents a genetic distance of 0.008 substitutions/site. Phylogenetic tree for NA gene with complete taxa identity is shown in S2 Fig.

Phylogenetic analysis of the HA sequences (1,758bp) shows that 67.3% (113/168) of Malaysian influenza B viruses from this study belonged to Yamagata lineage, while 32.7% (55/168) viruses belonged to Victoria lineage (Fig 2, S1 Fig). In contrast, evaluation of the NA sequences (1,401bp) shows that all 166 viruses from this study belonged to the Yamagata lineage (Fig 3, S2 Fig). Both phylogenies indicate that all Malaysian Victoria viruses detected had Victoria-lineage HA and Yamagata-lineage NA. This inter-lineage reassortment has long been seen in B/Brisbane/60/2008-like viruses in previous studies [13, 18, 43, 44], which were derived from B/Brisbane/32/2002-like viruses. They have a HA gene segment that evolved from B/Shangdong/7/97-like viruses of Victoria lineage and NA gene segment that evolved from B/Sichuan/379/99-like viruses of Yamagata lineage [39].

Our 2012–2014 Malaysian influenza B viruses were grouped into 3 major clades in the HA and NA phylogenies: Yamagata Clade 3 (Yam-3), Yamagata Clade 2 (Yam-2) and Victoria Clade 1 (Vic-1A and Vic-1B), based on the recent WHO genetic groupings [23, 24] (Figs 2 and 3, S1 and S2 Figs). In Yam-3, two well-supported (>70% bootstrap) subclades, represented by B/Wisconsin/01/2010-like (Wisconsin/01-like) and B/Stockholm/12/2011-like (Stockholm/12-like) were identified. From there we detected a major group of intra-clade reassortants sharing HA and NA genes from Wisconsin/01-like and Stockholm/12-like subclades, respectively. The earliest strain (B/Malaysia/U2462/2013) of such HA-NA reassortant form was sampled on 31^st^ May 2013. By including other Malaysian influenza B viruses isolated from IMR, the number of similar intra-clade reassortants was found to increase to 32 in Malaysia by June 2014, and they formed a marginally-supported new monophyletic subclade (>80% bootstrap value for NA phylogeny but <60% for HA phylogeny) according to WHO recommendations [45].

We further assessed the phylogenetic positions of 2,005 global full-length HA and NA sequences retrieved from GISAID and GenBank databases with a collection year of 2012–2015, and we identified 446 sequences from other countries that fell into this new subclade which had HA and NA gene that derived from Wisconsin/01-like subclade and Stockholm/12-like subclade respectively (S3 and S4 Figs). Interestingly, a recent WHO recommended candidate vaccine strain for the Northern and Southern Hemisphere of the 2015–2016 influenza season—B/Phuket/3073/2013 strain from Thailand, fell into this new subclade as well [23, 24]. Thus, we conveniently denote this new subclade as Phuket/3073-like subclade. Though it is noteworthy that B/Malaysia/U2462/2013 was by far the earliest strain detected in this subclade (on 31st May 2013)- 5 months before B/Phuket/3073/2013 was sampled (on 21st November 2013). The earliest non-Malaysian strain (B/Dominican Republic/7672/2013) was sampled in the Dominican Republic on 12th July 2013 (S3–S6 Figs).

**Fig 4:**
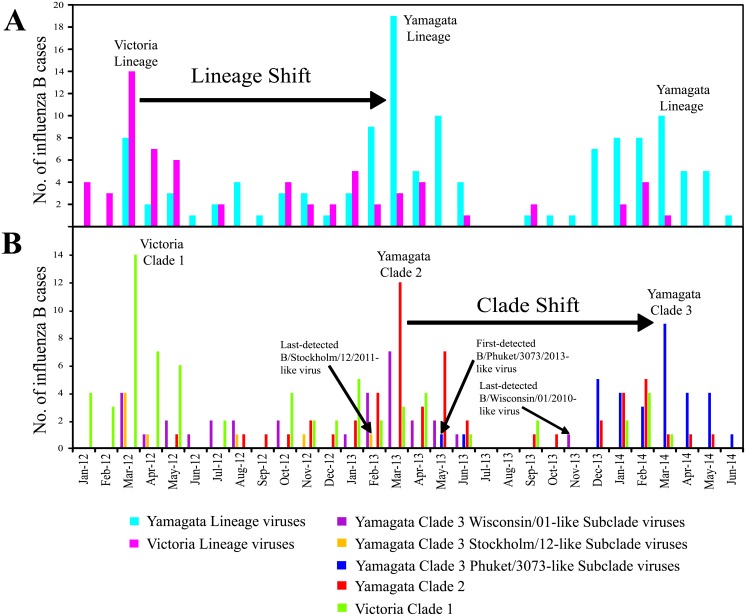
Lineage and clade shift of influenza B viruses between 2012 and 2014. Monthly distribution of influenza B viruses by (A) lineage and (B) clade.

Phylogenetic classification of Malaysian influenza B viruses into lineages, clades and subclades allowed better understanding of their prevalence and temporal distribution in Malaysia with greater details (Fig 4). Based on HA phylogeny, the overall prevalence of Yam-3, Yam-2 and Vic-1 from February 2012 to May 2014 in this study were 38.7% (65/168), 28.6% (48/168) and 32.7% (55/168), respectively. The prevalence of B/Phuket/3073/2013-like intra-clade reassortants was 17.3% (29/168). Considering the number of influenza B virus detected over time (including those viruses isolated from IMR), a lineage shift (change) from Victoria to Yamagata occurred between 2012 and 2013 (Fig 4A). Although co-circulation of Yamagata lineage and Victoria lineage was observed, the Victoria lineage predominated briefly first in 2012 followed by the Yamagata lineage in 2013 that remained dominant since then. However, between 2013 and 2014, we observed a clade shift inside the Yamagata lineage: from Yam-2 to Yam-3 (Fig 4B). Notably, all Yam-3 viruses that predominated in 2014 were B/Phuket/3073/2013-like intra-clade reassortants from Phuket/3073-like subclade.

Evidences from phylogenetic and prevalence analyses in a Malaysian context suggest that a single intra-clade reassortment event occurring between Wisconsin/01-like and Stockholm/12-like subclades may contribute to the recent predomination of Phuket/3073-like subclade. The HA gene that derived from Stockholm/12-like subclade was last detected on February 2013, but the NA gene derived from this subclade was later seen in all Phuket/3073-like subclade viruses, which had HA gene that derived from Wisconsin/01-like subclade (Fig 4). Another single intra-clade reassortment event was also detected within Vic-1 where a Malaysian virus (B/Malaysia/U1429/2013) had HA gene from Vic-1B (represented by B/Odessa/3886/2010) and NA gene from Vic-1A (represented by B/Brisbane/60/2008) (Figs 2 and 3, S1 and S2 Figs). Furthermore, in Yamagata lineage, we detected an inter-clade reassortant (B/Malaysia/U2214/2013), possessing HA gene from Yam-2 (represented by B/Massachusetts/02/2012) and NA gene from Yam-3 (represented by B/Wisconsin/01/2010) (Figs 2 and 3, S1 and S2 Figs). However, these two reassortant forms were found in single virus strains, suggesting that they were sporadic reassortant viruses.

### Evolutionary Dynamics of Phuket/3073-like Subclade

The emergence time of Phuket/3073-like subclade was investigated, by including all 32 Malaysian viruses and 446 global influenza B viruses retrieved from GISAID that were phylogenetically grouped under this subclade (S3–S6 Figs).

**Fig 5:**
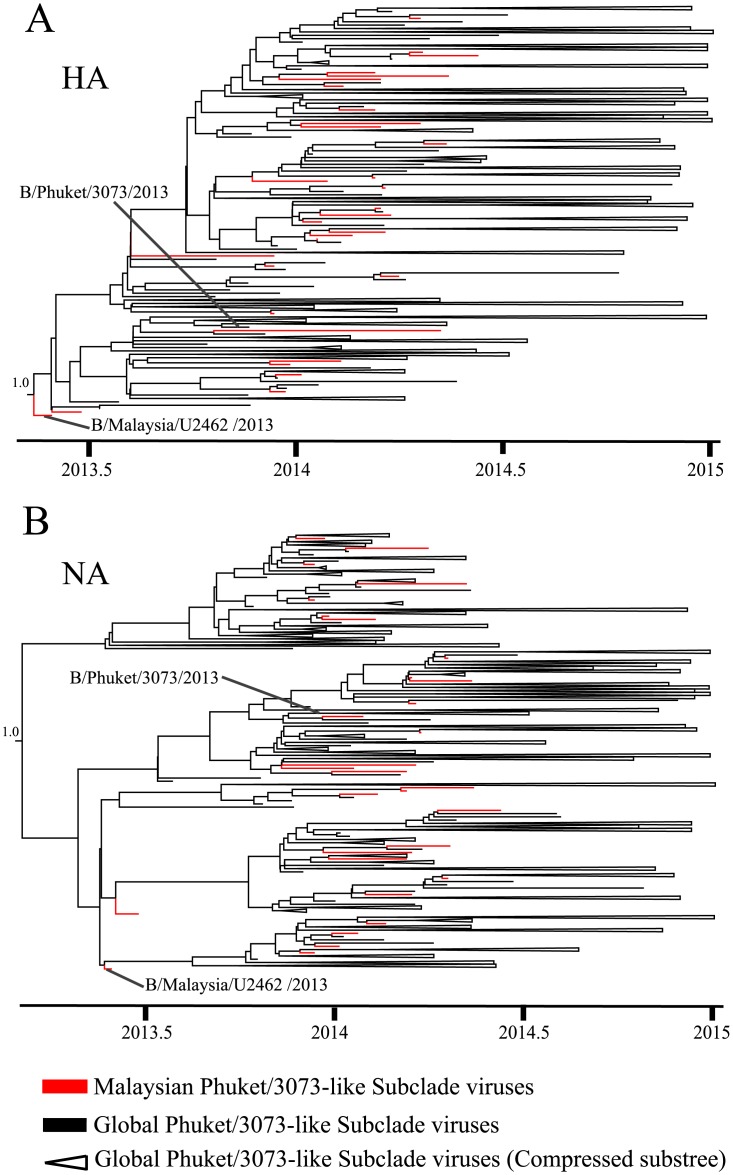
Maximum clade credibility (MCC) tree reconstruction of Phuket/3073-like subclade. (A) HA gene and (B) NA gene. The 95% highest posterior density (HPD) for the ancestral node is indicated. Timescale is shown at the bottom of the tree. MCC tree with complete taxa identity is shown in S5 and S6 Figs.

Applying a relaxed molecular clock model in the Bayesian MCMC analysis obtained the estimates of the evolutionary rates for both HA and NA gene at 2.2 (95% HPD: 1.9–2.6) x 10^−3^ and 3.0 (2.5–3.4) x 10^−3^ substitutions/site/year, respectively (Table 2), similar to previously published data [39, 41]. The time of the most recent common ancestor (tMRCA) for HA and NA gene of Phuket/3073-like subclade were estimated to be 2013.2 (March 2013) (in year fraction; 95% HPD: 2012.9–2013.4, November 2012-May 2013) and 2013.1 (February 2013) (95% HPD: 2012.8–2013.4, October 2012-May 2013) respectively (Table 2). These estimates collectively suggested that the intra-clade reassortment event could have occurred in March 2013 or earlier, which is about 10 months before the B/Phuket/3073/2013 vaccine strain was first isolated in Thailand. The maximum clade credibility (MCC) tree reconstruction for HA (Fig 5A) and NA (Fig 5B) gene of Phuket/3073-like subclade showed that B/Malaysia/U2462/2013 virus consistently occupied the basal position of this subclade while other Malaysian viruses intermingled with global viruses. This suggested that Malaysia could possibly be the place where early Phuket/3073 subclade-like viruses have been circulating, from which the virus was disseminated to other places, and that re-introduction back to Malaysia have also occurred.

**Table 2:** Evolutionary rate and age of influenza B Phuket/3073-like subclade.

Gene	Rates of evolution(95% HPD)	tMRCA (95% HPD)(Year Fraction)	tMRCA (95% HPD)(Month & Year)
**HA**	2.2 (1.9–2.6) x10^-3^	2013.2 (2012.9–2013.4)	March 2013 (November 2012 –May 2013)
**NA**	3.0 (2.5–3.4) x10^-3^	2013.1 (2012.8–2013.4)	February 2013 (October 2012 –May 2013)

tMRCA: time of the most recent common ancestor; HPD: highest posterior density

### Influenza B HA and NA Protein Sequence Analysis

In general, all Malaysian Vic-1, Yam-2 and Yam-3 viruses shared more than 99.0% average nucleotide and amino acid similarity with representative candidate vaccine strains B/Brisbane/60/2008, B/Massachusetts/02/2012 and B/Wisconsin/01/2010, for both HA and NA gene (S3 Table). These similarities generally decreased every year, reflecting that the Malaysian viruses were evolving away from respective candidate vaccine strains (S3 Table).

A total of 99 amino acid substitutions on the HA protein were detected in all three clades when compared to respective representative candidate vaccine strains (S4–S6 Tables). Among these substitutions, only 15 substitutions occurred on positively selected sites of the four major epitopes of HA1 subunit and their surrounding regions as reported previously [46–49] (Table 3). In particular, N116K substitution (shared by 64 Yam-3 viruses) and H122Y substitution (shared by 22 Vic-1 viruses) were found in the 120-loop of HA1 subunit. I146V substitution was shared by 67 Vic-1 viruses in the 150-loop. These substitutions occurred on regions that serve as antibody binding sites, and could contribute to the structural alteration of the HA protein, leading to changes in antigenicity [15, 48, 50]. Notably, D194N (D194N based on B/HongKong/73 numbering; D197N based on B/Brisbane/60/2008 vaccine strain numbering; D196N based on B/Massachusetts/02/2012 and B/Wisconsin/01/2010 vaccine strain numbering) substitution was found on the 190-helix hot spot (HA_1_ 194–196, based on B/HongKong/73 numbering) which is a potential glycosylation site [47]. This substitution (N-glycosylation) was detected in all Malaysian viruses, and was also found in the recent Thailand [18] and Beijing [15] strains. Substitution at this amino acid position has been known to alter the antigenicity of the virus [51] and may help to shield the 190-helix epitope from antibody recognition [47], though the role of D194N substitution in increasing viral fitness requires additional investigation.

**Table 3:** Amino acid substitutions found on the HA protein of influenza B viruses.

Subunit	Epitope (Residue location)	Vic-1 (n = 67)	Yam-2 (n = 53)	Yam-3 (n = 71)
**HA1**	**120-loop (116–137)**	***H122Y (22)***		***N116K (64)***
				***T121A (10)***
				V124A (2)
	**150-loop (141–150)**	***I146V (67)***		
	**160-loop (162–167)**	A169E (2)		
	**190-helix (194–202)**	***D197N (66)***	***D196N (53)***	***D196N (71)***
		***T199N (2)***		***S202N (6)***
		***A202E (21)***		
	**Surrounding region**	V6A (2)	V6I (18)	V11A (2)
		G68S (2)	***T75N (2)***	V29A (7)
		V90I (6)	V176I (2)	L172Q (30)
		***A154E (8)***	***Y178N (3)***	S207P (3)
		N171S (4)	***G229D (3)***	M251V (13)
		V177I (5)	***T234R (3)***	***K298E (64)***
		***I180V (3)***		E312K (64)
		V190I (3)		
		K209N (18)		
		I267V (2)		
**HA2**		N46D (6)		

Only amino acid substitutions shared by 2 viruses or more are reported in this table. Substitutions are compared with vaccine strains of respective clades (S4–S6 Tables). Amino acid substitutions are numbered according to respective vaccine strains (B/Brisbane/60/2008 for Vic-1, B/Massachusetts/02/2012 for Yam-2, B/Wisconsin/01/2010 for Yam-3). Bold and italic text indicate previously reported positively selected sites. The number of Malaysian influenza viruses having the substitution is indicated inside parenthesis.

On the NA protein, a total of 127 amino acid substitutions were found in all 3 clades when compared to respective representative candidate vaccine strains (S7–S9 Tables). Only 2/68 (2.94%) Malaysian viruses from Vic-1 displayed H274Y substitution in the NA active sites, which was a neuraminidase inhibitor (oseltamivir)-resistant substitution [52]. No other substitutions were observed on the NA active sites that confer to neuraminidase inhibitor resistance, as reported elsewhere [53–57], suggesting that the virus would still be sensitive to neuraminidase inhibitors. Notably, three Malaysian viruses from Vic-1 and all viruses from Yam-3 including the Phuket/3073-like subclade had a novel D463N substitution on a non-active site, which introduced a potential glycosylation at that position, potentially shielding the nearby active site. Further functional study of this substitution is warranted.

Comparison of protein sequences with candidate vaccine strains (B/Brisbane/60/2008 for Vic-1, B/Massachusetts/02/2012 for Yam-2 and B/Wisconsin/01/2010 for Yam-3) allowed us to identify several signature amino acid substitutions shared by major clades and subclades. Interestingly, 21 out of 32 Malaysian viruses within Phuket/3073-like subclade had an additional L172Q substitution apart from sharing N116K, K298E and E312K substitutions on the HA protein with Wisconsin/01-like subclade (Table 4). However, in the NA protein, all viruses within Phuket/3073-like subclade had additional I45V, E320K and E340D substitutions besides sharing L73P and K343E substitutions on the NA protein with Stockholm/12-like subclade. These molecular signatures further corroborate the phylogenetic tree that the HA and NA protein of Phuket/3073-like subclade was originated from the Wisconsin/01-like and Stockholm/12-like subclades, respectively.

**Table 4:** Major signature amino acid substitutions. These substitutions are summarized from S4–S9 Tables.

	Vic-1				Yam-3		
	Vic-1A						
Protein	V1A-1	V1A-2	Vic-1B	Yam-2	Stockholm/12-like	Wisconsin/01-like	Phuket/3073-like
**HA**	***N75K***			R48K	***S150I***		
	***N165K***			P108A	***N165Y***		
	***S172P***			T181A	***D196N***		
	***I146V***		L58P	D196N	***G229D***		
	H122Y				V29A	***N116K***	
	A202E				L172Q	***K298E***	
					M251V	***E312K***	
							L172Q
**NA**	***I204V***			I148V	***Q42R***		
	***A358E***			T106I	***A68T***		
	***D329N***			S295R	***T125K***		
	***N340D***				***K186R***		
	A389T				***D463N***		
		S295R			***A465T***		
		E358K			***D340N***		N340D
					L73P		L73P
					K343E		K343E
							I45V
							E320K

Amino acid substitutions are numbered according to vaccine strains (B/Brisbane/60/2008 for Vic-1, B/Massachusetts/02/2012 for Yam-2, B/Wisconsin/01/2010 for Yam-3). Bold and italic text indicates substitutions shared by two subclades or more.

Besides, within Vic-1 lineage, additional H122Y and A202E substitutions were detected on the HA protein and A389T substitution on the NA protein. These substitutions were shared by 22 viruses within Vic-1A (characterized by I146V substitution on HA protein), which were grouped under V1A-1 subclade (Figs 2 and 3, S1 and S2 Figs). The remaining strains within Vic-1A did not acquire H122Y and A202E substitutions on the HA protein but share additional S295R and E358K substitutions on the NA protein. They were grouped under V1A-2 subclade (Figs 2 and 3, S1 and S2 Figs).

### Comparison of Demographic and Clinical Characteristics between Influenza Lineages

A comparison of demographic and clinical characteristics between Yamagata and Victoria lineage-infected patients is shown in Table 5. Patients infected by the Yamagata lineage viruses were significantly older than patients infected by the Victoria lineage virus, with mean age (years±S.D) for Yamagata being 43.58±18.22 versus Victoria being 32.11±16.18 (*p*<0.001; Independent Samples t-Test). Similarly, a higher proportion of adults over 56 years old were infected by the Yamagata lineage than the Victoria lineage (51.8% versus 14.3%; *p* = 0.006; Pearson’s chi-square test). In contrast, we found no significant difference in day(s) onset of symptoms (number of days after the first appearance of symptoms) between the two different lineages. Besides, only two symptoms: nasal congestion and sore throat were found to have an association with patients infected by Victoria lineage viruses (*p* = 0.033, OR = 2.22 and *p* = 0.020, OR = 2.49 respectively; Pearson’s chi-square test).

**Table 5:** Comparison on the demographic and clinical characteristics of patients infected by influenza B Victoria and Yamagata lineages.

		Victoria Lineage	Yamagata Lineage			Yamagata vs Victoria (ref.)				
Characteristics		(n = 56)	(n = 114)	*p*	OR (95% CI)	B	SE	*p*	aOR	95% CI
**Demographic Features**										
Mean (± S.D.) age in years		32.11 ± 16.18	43.58 ± 18.22	**<0.001** ^a^	-	-	-	-	-	-
No. (%) of patients≤ 24 yrs old		21 (37.5)	20 (17.5)	**0.006** ^b^	-	ref.		0.085^c^	ref.	
No. (%) of patients25-55 yrs old		27 (48.2)	35 (30.7)		-	0.576	0.459	0.210^c^	1.779	0.723–4.377
No. (%) of patients≥ 56 years old		8 (14.3)	59 (51.8)		-	1.466	0.662	**0.027** ^c^	**4.33**	**1.184–15.838**
**Clinical Features**										
Median (IQR) days onset of symptoms		4 (2–6)	4 (3–5)	0.670^a^	-	-0.032	0.089	0.722^c^	0.969	0.814–1.153
No. (%) of patients with:										
Sneezing	Yes	44 (78.6)	81 (71.1)	0.296^b^	1.49 (0.70–3.18)	-0.497	0.522	0.341^c^	0.608	0.219–1.692
	No	12 (21.4)	33 (28.9)		ref.				ref.	
Nasal Discharge	Yes	43 (76.8)	88 (77.2)	0.953^b^	0.98 (0.46–2.09)	0.565	0.523	0.280^c^	1.76	0.631–4.910
	No	13 (23.2)	26 (22.8)		ref.				ref.	
Nasal Congestion	Yes	44 (78.6)	71 (62.3)	**0.033** ^b^	**2.22 (1.06–4.67)**	-1.042	0.513	**0.042** ^c^	**0.353**	**0.139–0.963**
	No	12 (21.4)	43 (37.7)		ref.				ref.	
Headache	Yes	40 (71.4)	89 (78.1)	0.341^b^	0.70 (0.34–1.46)	1.282	0.514	**0.013** ^c^	**3.603**	**1.316–9.896**
	No	16 (28.6)	25 (21.9)		ref.				ref.	
Sore throat	Yes	46 (82.1)	74 (64.9)	**0.02** ^b^	**2.49 (1.14–5.45)**	-1.42	0.542	**0.009** ^c^	**0.242**	**0.084–0.699**
	No	10 (17.9)	40 (35.1)		ref.				ref.	
Hoarseness of voice	Yes	43 (76.8)	93 (81.6)	0.463^b^	0.75 (0.34–1.63)	0.874	0.523	0.095^c^	2.398	0.860–6.683
	No	13 (23.2)	21 (18.4)		ref.				ref.	
Muscle ache	Yes	45 (80.4)	97 (85.1)	0.434^b^	0.72 (0.31–1.66)	-0.467	0.583	0.423^c^	0.627	0.200–1.964
	No	11 (19.6)	17 (14.9)		ref.				ref.	
Cough	Yes	54 (96.4)	109 (95.6)	0.802^b^	1.24 (0.23–6.60)	-0.681	1.059	0.520^c^	0.506	0.064–4.032
	No	2 (3.6)	5 (4.4)		ref.				ref.	

n: number of patients; OR: odds ratio; CI: confidence interval; B: coefficient; SE: standard error; SD: standard deviation; aOR: adjusted odds ratio; ref: reference group; *p*: level of significance (2-tailed) at the 0.05 level

^a^p-value calculated by Independent Samples t-Test

^b^p-value calculated by Pearson’s Chi-square test/Fischer’s Exact test

^c^p-value calculated by Binary Logistic Regression

In addition, when binary logistic regression was used to predict the likelihood of patients infected with either Yamagata or Victoria lineage virus, four factors made a unique statistically significant contribution (*p*<0.05) to the regression model: patients ≥56 years old, nasal congestion, headache and sore throat. The strongest predictors of a Yamagata lineage-infection were headache and age ≥56 years old (aOR: 3.603, 95% CI: 1.316–9.896, *p*: 0.013 and aOR: 4.33, 95% CI: 1.184–15.838, *p*: 0.027, respectively), while the strongest predictors of a Victoria lineage-infection were nasal congestion and sore throat (aOR: 2.833 (1/0.353) 95% CI: 1.038–7.194 (1/0.139–1/0.963), *p*:0.042 and aOR: 4.132 (1/0.242), 95% CI: 1.431–11.905 (1/0.084–1/0.699), *p*: 0.009, respectively) (Table 5).

## Discussion

Environmental factors such as higher amount of rainfall, higher relative humidity and colder temperature were found to increase the risk of seasonal influenza transmission [58, 59]. These factors may affect virus survivability by lengthening the protective effect of droplets on viruses trapped on fomites or aerosols [26, 58–60]. In particular, colder temperature has been found promoting influenza virus survival in aerosols [25], whereas higher humidity might increase the amount of virus particles that is deposited on the surface, hence encouraging contact transmission of the virus [57]. Malaysia has a tropical equatorial climate accompanied by two monsoon seasons, the Southwest Monsoon (May to September) and Northeast Monsoon (November to March). The Northeast Monsoon brings in more rainfall compared to the Southwest Monsoon [61]. In this study, as expected, we observed that the peak of the total amount of rainfall, number of rain days and relative humidity coincided with the Malaysian Northeast Monsoon season (Fig 1). The lowest ground temperature was also recorded during this period. Based on bivariate correlation, higher number of influenza cases was found to associate significantly with lower temperature and higher amount of rainfall, rain days and relative humidity (Table 1). Similar association for influenza A was reported in previous studies as well [26, 28]. However, when partial correlation and multiple linear regression was performed, only ground temperature shows significant negative association with no. of influenza cases and was the strongest predictor among all meteorological factors (Table 1). The actual causal relationship between ground temperature and influenza seasonality remains in question. Though, these findings suggest that the influenza A and B seasonal activity probably coincided with a combination of colder and rainier Malaysian Northeast Monsoon instead of the Southwest Monsoon. A significant negative correlation between particulate matter (PM10) and influenza activity was also found in this study (Table 1). Similar relationship was reported in Southern China [62, 63], which postulated that lower PM10 would result in higher ultraviolet radiation (UVR) that may decrease immune function. However, the role of UVR in increasing host susceptibility to influenza remains controversial and requires further investigation.

There was a difference in the overall prevalence between influenza A and B viruses during the study period. The number of influenza B cases was consistently lower compared to influenza A cases every year, and there is a time lag between their peak activities (Fig 1C). On the basis of several reports indicating lower rates of nucleotide mutation and selection pressure in influenza B viruses compared to influenza A viruses [39, 64], it is tempting to suggest that they may play a role in the lower prevalence of influenza B viruses. Though such description remains speculative as prevalence studies could be affected by sampling artifacts or the scale of surveillance. While both influenza and B viruses continue to co-circulate, we also observed a slight increase of influenza B incidence when influenza A incidence decreased) (Fig 1C) The increase of influenza B incidence also coincided with shifts in the predominant influenza B lineage (from Victoria lineage in 2012 to Yamagata lineage in 2013) and clade (from Yam-2 in 2013 to Yam-3 in 2014) (Fig 4). The mechanism on how the decrease of influenza A incidence may lead to a change of influenza B lineage and clade requires further investigation.

However, we hypothesize that the turnover of antigenically distinct lineages from Victoria lineage in 2012 to Yamagata lineage in 2013 could be a result of immune selection due to accumulated herd immunity in the human population [39]. We suggest that the less dominant Yamagata lineage with distinct antigenicity may regain dominance when the predominating Victoria lineage has induced sufficient herd immunity in the hosts, either through recovery from infection or vaccination. It was expected that such alteration of dominance would continue and hence Victoria lineage would become dominant again in 2014. Surprisingly, the Yamagata lineage continued its domination in the influenza B viral population with a clade shift from Yam-2 in 2013 to Yam-3 in 2014 that consists mainly of B/Phuket/3073/2013-like viruses (Fig 4).

Besides possessing an intra-clade reassortment property (HA from Wisconsin/01-like subclade and NA from Stockholm/12-like subclade) (Figs 2 and 3), Phuket/3073-like subclade viruses also shared several signature amino acid substitutions (Table 4). Haemagglutination inhibition (HI) tests by WHO have shown that the representative B/Phuket/3073/2013 strains have acquired significant antigenic drift (≥4 folds of titer reduction) from the B/Wisconsin/1/2010 strains and (2 folds of titer reduction) from the B/Stockholm/12/2011 strains, in which both are representatives of B/Phuket/3073/2013 HA and NA parental clades, respectively [65]. Whether the signature amino acid substitutions may play a role in antigenic drift will require further in-depth molecular experiments to confirm, as antigenic characterization was not performed such that the detection of antigenic drift relied on prediction based on protein sequences. Though, overall, it is worth highlighting that a combination of meteorological factors, influenza population prevalence, genetic reassortment and antigenic drift and possibly other factors (vaccination uptake by the susceptible young or elderly population, socio-economic factors etc.) may shape the epidemiological and evolutionary dynamics of influenza B viruses in the lineage, clade and subclade levels.

Our analysis on the evolutionary dynamics of Phuket/3073-like subclade provides further evidence that Southeast Asia region with tropical and subtropical climate might be a regional and global hub for the emergence of novel influenza viruses [20, 22]. Notably, there is almost a two-year difference between the estimated time of intra-clade reassortment event (February-March 2013) (Table 2) and time when B/Phuket/3073/2013 vaccine strain was announced on September 2014 for the Southern Hemisphere and on February 2015 for the Northern Hemisphere [23]. This suggests that influenza B surveillance in Malaysia and other Southeast Asian countries should be intensified for early detection of emerging strains with epidemic potential.

Our observation of notable age difference between influenza B lineages, with Yamagata viruses were more likely to infect the older adults (≥56 years old) in the population, has also been observed in previous studies (Table 5) [14, 42]. This could be the result of a difference in background population immunity, in which older adults with weaker immunity are more susceptible to the current Yamagata strains due to their recent evolution. However, lineage-specific transmissibility among older adults is still currently unclear. We also observed that Yamagata-infected older patients were more likely to experience headache while Victoria-infected patients were more likely to experience nasal congestion and sore throat. Since this is the first association found between these symptoms with specific lineages, it would require additional data and studies to provide a more conclusive evidence of this association.

In summary, this study highlights the importance of continuous surveillance of influenza B viruses in order to better understand the current epidemiology and evolutionary dynamics of these viruses in Malaysia. The main limitation of this study is that only HA and NA surface genes were sequenced and analyzed, which were unable to detect the reassortment involving other internal genes. Phylogenetic analysis of all gene segments will provide a better understanding on influenza B evolution.

## Supporting Information

S1 Fig Phylogenetic analysis of the HA gene of influenza B viruses in Malaysia from 2012 to 2014 using the maximum likelihood (ML) method with complete taxa identity. (PDF)

S2 Fig Phylogenetic analysis of the NA gene of influenza B viruses in Malaysia from 2012 to 2014 using the maximum likelihood (ML) method with complete taxa identity. (PDF)

S3 Fig Phylogenetic analysis of the HA gene of Global and Malaysian Yamagata Clade 3 viruses isolated from 2012 to 2014 using the neighbor-joining (NJ) method (Kimura’s two-parameter distance model and 1,000 bootstrap replicates, as implemented in MEGA 6.0) with complete taxa identity. (PDF)

S4 Fig Phylogenetic analysis of the NA gene of Global and Malaysian Yamagata Clade 3 viruses isolated from 2012 to 2014 using the neighbor-joining (NJ) method (Kimura’s two-parameter distance model and 1,000 bootstrap replicates, as implemented in MEGA 6.0) with complete taxa identity. (PDF)

S5 Fig Maximum clade credibility (MCC) tree reconstruction of HA Gene of Phuket/3073-like subclade viruses with complete taxa identity. (PDF)

S6 Fig Maximum clade credibility (MCC) tree reconstruction of NA Gene of Phuket/3073-like subclade viruses with complete taxa identity. (PDF)

S1 Table Primer sets, Reagents and Thermocycling Conditions used for two-step Reverse-Transcription Polymerase Chain Reaction (RT-PCR) for Amplification of Influenza B HA and NA genes. Source: London WHO Collaborating Centre, May 2011; WHO information for molecular diagnosis of influenza virus.
(PDF)

S2 Table Influenza B virus clinical isolates sequenced in this study. (PDF)

S3 Table Average nucleotide and amino acid sequence homology. (PDF)

S4 Table Amino acid substitutions found on the HA protein for all Malaysian Victoria Clade 1 viruses (n = 67). Grey highlight indicates major signature amino acid substitutions. Substitutions are compared with B/Brisbane/60/2008 vaccine strain.
(PDF)

S5 Table Amino acid substitutions found on the HA protein for all Malaysian Yamagata Clade 2 viruses (n = 53). Grey highlight indicates major signature amino acid substitutions. Substitutions are compared with B/Massachusetts/02/2012 vaccine strain.
(PDF)

S6 Table Amino acid substitutions found on the HA protein for all Malaysian Yamagata Clade 3 viruses (n = 71). Grey highlight indicates major signature amino acid substitutions. Substitutions are compared with B/Wisconsin/01/2010 vaccine strain.
(PDF)

S7 Table Amino acid substitutions found on the NA protein for all Malaysian Victoria Clade 1 viruses (n = 68). Grey highlight indicates major signature amino acid substitutions. Substitutions are compared with B/Brisbane/60/2008 vaccine strain.
(PDF)

S8 Table Amino acid substitutions found on the NA protein for all Malaysian Yamagata Clade 2 viruses (n = 52). Grey highlight indicates major signature amino acid substitutions. Substitutions are compared with B/Massachusetts/02/2012 vaccine strain.
(PDF)

S9 Table Amino acid substitutions found on the NA protein for all Malaysian Yamagata Clade 3 viruses (n = 69). Grey highlight indicates major signature amino acid substitutions. Substitutions are compared with B/Wisconsin/01/2010 vaccine strain.
(PDF)

## References

[1] Yamashita M, Krystal M, Fitch WM, Palese P. Influenza B virus evolution: co-circulating lineages and comparison of evolutionary pattern with those of influenza A and C viruses. Virology. 1988;163(1):112–22. 3267218 10.1016/0042-6822(88)90238-3

[2] Lamb RA, Choppin PW. The gene structure and replication of influenza virus. Annual Review of Biochemistry. 1983;52(1):467–506. 10.1146/annurev.bi.52.070183.002343 6351727

[3] Yoon S-WW, Webby RJ, Webster RG. Evolution and ecology of influenza A viruses. Current Topics in Microbiology and Immunology. 2014;385:359–75. 10.1007/82_2014_396 24990620

[4] Rota PA, Wallis TR, Harmon MW, Rota JS, Kendal AP, Nerome K. Cocirculation of two distinct evolutionary lineages of influenza type B virus since 1983. Virology. 1990;175(1):59–68. 2309452 10.1016/0042-6822(90)90186-u

[5] McCullers JA, Wang GC, He S, Webster RG. Reassortment and insertion-deletion are strategies for the evolution of influenza B viruses in nature. Journal of Virology. 1999;73(9):7343–8. 10438823 10.1128/jvi.73.9.7343-7348.1999 PMC104260

[6] Paul Glezen W, Schmier JK, Kuehn CM, Ryan KJ, Oxford J. The burden of influenza B: a structured literature review. American Journal of Public Health. 2013;103(3):e43–e51. 10.2105/AJPH.2012.301137 23327249 PMC3673513

[7] Rota PA, Hemphill ML, Whistler T, Regnery HL, Kendal AP. Antigenic and genetic characterization of the haemagglutinins of recent cocirculating strains of influenza B virus. The Journal of General Virology. 1992;73 (1):2737–42. 1402807 10.1099/0022-1317-73-10-2737

[8] Kanegae Y, Sugita S, Endo A, Ishida M, Senya S, Osako K, et al Evolutionary pattern of the hemagglutinin gene of influenza B viruses isolated in Japan: cocirculating lineages in the same epidemic season. Journal of Virology. 1990;64(6):2860–5. 2335820 10.1128/jvi.64.6.2860-2865.1990 PMC249468

[9] Glezen WP. The changing epidemiology of respiratory syncytial virus and influenza: impetus for new control measures. The Pediatric Infectious Disease Journal. 2004;23(11):S202–S6. 15577574 10.1097/01.inf.0000144662.86396.07

[10] McCullers JA, Saito T, Iverson AR. Multiple Genotypes of Influenza B Virus Circulated between 1979 and 2003. Journal of Virology. 2004;78(23):12817–28. 15542634 10.1128/JVI.78.23.12817-12828.2004 PMC525012

[11] Chi XS, Bolar TV, Zhao P, Rappaport R, Cheng S-M. Cocirculation and Evolution of Two Lineages of Influenza B Viruses in Europe and Israel in the 2001–2002 Season. Journal of Clinical Microbiology. 2003;41(12):5770–3. 14662979 10.1128/JCM.41.12.5770-5773.2003 PMC309036

[12] Ghazanfar A, Haitham MA, Fahad NA. Hemagglutinin and neuraminidase genes of influenza B viruses circulating in Riyadh, Saudi Arabia during 2010–2011: Evolution and sequence analysis. Journal of Medical Virology. 2014;86(6):1003–16. 10.1002/jmv.23819 24150926

[13] Yang J-RR, Huang Y-PP, Chang F-YY, Hsu L-CC, Lin Y-CC, Huang H-YY, et al Phylogenetic and evolutionary history of influenza B viruses, which caused a large epidemic in 2011–2012, Taiwan. PLoS One. 2012;7(10):e47179 10.1371/journal.pone.0047179 23071751 PMC3470568

[14] Tan Y, Guan W, Lam TT, Pan S, Wu S, Zhan Y, et al Differing epidemiological dynamics of influenza B virus lineages in Guangzhou, southern China, 2009–2010. Journal of Virology. 2013;87(22):12447–56. 10.1128/JVI.01039-13 24027322 PMC3807886

[15] Fang Q, Gao Y, Chen M, Guo X, Yang X, Wei L. Molecular epidemiology and evolution of influenza A and B viruses during winter 2013–2014 in Beijing, China. Archives of Virology. 2015;160(4):1083–95. 10.1007/s00705-015-2362-x 25676826

[16] Barr IG, Komadina N, Hurt A, Shaw R, Durrant C, Iannello P, et al Reassortants in recent human influenza A and B isolates from South East Asia and Oceania. Virus Research. 2003;98(1):35–44. 14609628 10.1016/j.virusres.2003.08.011

[17] Dapat C, Saito R, Kyaw Y, Naito M, Hasegawa G, Suzuki Y, et al Epidemiology of human influenza A and B viruses in Myanmar from 2005 to 2007. Intervirology. 2009;52(6):310–20. 10.1159/000237738 19776616

[18] Tewawong N, Suwannakarn K, Prachayangprecha S, Korkong S, Vichiwattana P, Vongpunsawad S, et al Molecular Epidemiology and Phylogenetic Analyses of Influenza B Virus in Thailand during 2010 to 2014. PLoS One. 2015;10(1):e0116302 10.1371/journal.pone.0116302 25602617 PMC4300180

[19] Jumat MR, Sugrue RJ, Tan B-HH. Genetic characterisation of influenza B viruses detected in Singapore, 2004 to 2009. BMC Research Notes. 2014;7(1):863. 25435177 10.1186/1756-0500-7-863 PMC4265450

[20] Colin AR, Terry CJ, Ian GB, Nancy JC, Rebecca JG, Vicky G, et al Influenza vaccine strain selection and recent studies on the global migration of seasonal influenza viruses. Vaccine. 2008;26:D31–D4. 19230156 10.1016/j.vaccine.2008.07.078

[21] Russell CA, Jones TC, Barr IG, Cox NJ, Garten RJ, Gregory V, et al The global circulation of seasonal influenza A (H3N2) viruses. Science. 2008;320(5874):340–6. 10.1126/science.1154137 18420927

[22] Trevor B, Steven R, Ian GB, Shobha B, Mandeep C, Nancy JC, et al Global circulation patterns of seasonal influenza viruses vary with antigenic drift. Nature. 2015;523(7559):217–20. 10.1038/nature14460 26053121 PMC4499780

[23] WHO. Influenza Centre London. September 2014 interim report. Report prepared for the WHO annual consultation on the composition of infuenza vaccine for the Southern Hemisphere 2015. 22nd-24th September 2014. 2014.

[24] WHO. Influenza Centre London. February 2015 interim report. Report prepared for the WHO annual consultation on the composition of influenza vaccine for the Northern Hemisphere 2015/16. 23rd -25th February 2015. 2015.

[25] Sam ICC, Su YC, Chan YF, Nor'E SS, Hassan A, Jafar FL, et al Evolution of influenza B virus in Kuala Lumpur, Malaysia between 1995 and 2008. Journal of Virology. 2015 10.1128/JVI.00708-15 PMC4542393 26136576

[26] Lowen AC, Mubareka S, Steel J, Palese P. Influenza virus transmission is dependent on relative humidity and temperature. PLoS Pathogens. 2007;3(10):1470–6. 17953482 10.1371/journal.ppat.0030151 PMC2034399

[27] Anice CL, John S. Roles of Humidity and Temperature in Shaping Influenza Seasonality. Journal of Virology. 2014;88(14):7692–5. 10.1128/JVI.03544-13 24789791 PMC4097773

[28] Chan PK, Mok HY, Lee TC, Chu IM, Lam W-YY, Sung JJ. Seasonal influenza activity in Hong Kong and its association with meteorological variations. Journal of Medical Virology. 2009;81(10):1797–806. 10.1002/jmv.21551 19697414

[29] Jackson GG, Dowling H, Spiesman IG, Boand AV. Transmission of the common cold to volunteers under controlled conditions. I. The common cold as a clinical entity. AMA Archives of Internal Medicine. 1958;(101):267–78. 13497324 10.1001/archinte.1958.00260140099015

[30] Perandin F, Pollara PC, Gargiulo F, Bonfanti C, Manca N. Performance evaluation of the automated NucliSens easyMAG nucleic acid extraction platform in comparison with QIAamp Mini kit from clinical specimens. Diagnostic Microbiology and Infectious Disease. 2009;64(2):158–65. 10.1016/j.diagmicrobio.2009.02.013 19500527

[31] Loens K, Bergs K, Ursi D, Goossens H, Ieven M. Evaluation of NucliSens easyMAG for automated nucleic acid extraction from various clinical specimens. Journal of Clinical Microbiology. 2007;45(2):421–5. 17166966 10.1128/JCM.00894-06 PMC1829055

[32] Hwang SM, Lim MS, Han M, Hong YJ, Kim TS, Lee HR, et al Comparison of xTAG Respiratory Virus Panel and Verigene Respiratory Virus Plus for Detecting Influenza Virus and Respiratory Syncytial Virus. Journal of Clinical Laboratory Analysis. 2014;29(2):116–21. 10.1002/jcla.21738 24796703 PMC6807105

[33] Pabbaraju K, Wong S, Tokaryk KL, Fonseca K, Drews SJ. Comparison of the Luminex xTAG respiratory viral panel with xTAG respiratory viral panel fast for diagnosis of respiratory virus infections. Journal of Clinical Microbiology. 2011;49(5):1738–44. 10.1128/JCM.02090-10 21411570 PMC3122679

[34] WHO. WHO information for molecular diagnosis of influenza virus. Updated March 2014. 2014.

[35] GISAID. EpiFlu™ database [1 March 2015]. Available from: http://platform.gisaid.org.

[36] Hall T. BioEdit: An important software for molecular biology. GERF Bulletin of Bioscience. 2011;2:60–1.

[37] Swofford DL. PAUP*: phylogenetic analysis using parsimony, version 4.0 b10. 2003.

[38] Drummond AJ, Suchard MA, Xie D, Rambaut A. Bayesian phylogenetics with BEAUti and the BEAST 1.7. Molecular biology and evolution. 2012;29(8):1969–73. 10.1093/molbev/mss075 22367748 PMC3408070

[39] Chen R, Holmes EC. The evolutionary dynamics of human influenza B virus. Journal of Molecular Evolution. 2008;66(6):655–63. 10.1007/s00239-008-9119-z 18504518 PMC3326418

[40] Dudas G, Bedford T, Lycett S, Rambaut A. Reassortment between Influenza B Lineages and the Emergence of a Coadapted PB1-PB2-HA Gene Complex. Molecular Biology and Evolution. 2015;32(1):162–72. 10.1093/molbev/msu287 25323575 PMC4271528

[41] Vijaykrishna D, Holmes EC, Joseph U, Fourment M, Su YC, Halpin R, et al The contrasting phylodynamics of human influenza B viruses. eLife. 2015;4:e05055 10.7554/eLife.05055 25594904 PMC4383373

[42] Sočan M, Prosenc K, Učakar V, Berginc N. A comparison of the demographic and clinical characteristics of laboratory-confirmed influenza B Yamagata and Victoria lineage infection. Journal of Clinical Virology. 2014;61(1):156–60. 10.1016/j.jcv.2014.06.018 25034374

[43] Denis KB, Bernard E, Monica M, Hannah K, Lukwago L, Josephine B, et al Genetic analysis of influenza B viruses isolated in Uganda during the 2009–2010 seasons. Virology Journal. 2013;10:11 10.1186/1743-422X-10-11 23289789 PMC3547786

[44] Na Z, Peng L, Jingfeng Y, Yongdong L, Jiuru Z, Han X, et al Molecular characterization of influenza B viruses isolated in east-central China in 2009–2010. Virus Genes. 2013;46(1):28–38. 10.1007/s11262-012-0826-6 23011776

[45] WHO. World Organisation for Animal Health/Food and Agriculture Organization (WHO/OIE/FAO) H5N1 Evolution Working Group. Revised and updated nomenclature for highly pathogenic avian influenza A (H5N1) viruses. Influenza Other Respir Viruses. 2013;8(3):384–8. 10.1111/irv.12230 PMC4181488 24483237

[46] Wang Q, Tian X, Chen X, Ma J. Structural basis for receptor specificity of influenza B virus hemagglutinin. Proceedings of the National Academy of Sciences. 2007;104(43):16874–9. 10.1073/pnas.0708363104 PMC2040455 17942670

[47] Wang Q, Cheng F, Lu M, Tian X, Ma J. Crystal structure of unliganded influenza B virus hemagglutinin. Journal of Virology. 2008;82(6):3011–20. 10.1128/JVI.02477-07 18184701 PMC2259021

[48] Shen J, Kirk BD, Ma J, Wang Q. Diversifying selective pressure on influenza B virus hemagglutinin. Journal of Medical Virology. 2009;81(1):114–24. 10.1002/jmv.21335 19031453 PMC2697605

[49] Ni F, Kondrashkina E, Wang Q. Structural basis for the divergent evolution of influenza B virus hemagglutinin. Virology. 2013;446(1):112–22. 24074573 10.1016/j.virol.2013.07.035 PMC3902124

[50] Tapasi R, Anurodh SA, Anupam M, Akhilesh CM, Mandeep SC, Harpreet K, et al Surveillance and molecular characterization of human influenza B viruses during 2006–2010 revealed co-circulation of Yamagata-like and Victoria-like strains in eastern India. Infection, Genetics and Evolution: Journal of Molecular Epidemiology and Evolutionary Genetics in Infectious Diseases. 2011;11(7):1595–601. 10.1016/j.meegid.2011.05.022 21708292

[51] Lugovtsev VY, Vodeiko GM, Strupczewski CM, Ye Z, Levandowski RA. Generation of the influenza B viruses with improved growth phenotype by substitution of specific amino acids of hemagglutinin. Virology. 2007;365(2):315–23. 17490701 10.1016/j.virol.2007.04.006

[52] Andrew JB, Tatiana B, Bindumadhav MM, Jianling A, Robert GW, Elena AG. Fitness costs for Influenza B viruses carrying neuraminidase inhibitor-resistant substitutions: underscoring the importance of E119A and H274Y. Antimicrobial Agents and Chemotherapy. 2014;58(5):2718–30. 10.1128/AAC.02628-13 24566185 PMC3993273

[53] Burnham AJ, Armstrong J, Lowen AC, Webster RG, Govorkova EA. Competitive Fitness of Influenza B Viruses with Neuraminidase Inhibitor-Resistant Substitutions in a Co-infection Model of the Human Airway Epithelium. Journal of Virology. 2015;89(8):4575–87. 10.1128/JVI.02473-14 25673705 PMC4442356

[54] Farrukee R, Leang S-KK, Butler J, Lee RT, Maurer-Stroh S, Tilmanis D, et al Influenza viruses with B/Yamagata- and B/Victoria-like neuraminidases are differentially affected by mutations that alter antiviral susceptibility. The Journal of Antimicrobial Chemotherapy. 2015:dkv065. 10.1093/jac/dkv065 25786478

[55] Oakley AJ, Barrett S, Peat TS, Newman J, Streltsov VA, Waddington L, et al Structural and functional basis of resistance to neuraminidase inhibitors of influenza B viruses. Journal of Medicinal Chemistry. 2010;53(17):6421–31. 10.1021/jm100621s 20695427 PMC2932999

[56] Hatakeyama S, Sugaya N, Ito M, Yamazaki M, Ichikawa M, Kimura K, et al Emergence of influenza B viruses with reduced sensitivity to neuraminidase inhibitors. JAMA: The Journal of the American Medical Association. 2007;297(13):1435–42. 17405969 10.1001/jama.297.13.1435

[57] Jackson D, Barclay W, Zürcher T. Characterization of recombinant influenza B viruses with key neuraminidase inhibitor resistance mutations. The Journal of Antimicrobial Chemotherapy. 2005;55(2):162–9. 15665027 10.1093/jac/dkh528

[58] Paynter S. Humidity and respiratory virus transmission in tropical and temperate settings. Epidemiology and Infection. 2014:1–9. 10.1017/S0950268814002702 PMC9507187 25307020

[59] Shaman J, Kohn M. Absolute humidity modulates influenza survival, transmission, and seasonality. Proceedings of the National Academy of Sciences of the United States of America. 2009;106(9):3243–8. 10.1073/pnas.0806852106 19204283 PMC2651255

[60] Scott FD. Seasonal Variation in Host Susceptibility and Cycles of Certain Infectious Diseases. Emerging Infectious Diseases. 2001;7(3):369–74. 11384511 10.3201/eid0703.010301 PMC2631809

[61] General Climate of Malaysia: Malaysian Meteorological Department; 2014 [1 March 2015]. Available from: http://www.met.gov.my/index.php?option=com_content&task=view&id=75&Itemid=1089

[62] Jun Q, Hong F, Zheng-hong C, Hong-nan Z, Li Z, Xiao-wen C. Impacts of Atmospheric Conditions on Influenza in Southern China. Part I. Taking Shenzhen City for Example. Open Journal of Air Pollution. 2012;1(3):59–66.

[63] Chit Ming W, Lin Y, Thuan Quoc T, Patsy Yuen Kwan C, King Pan C, Thomas GN, et al Modification by Influenza on Health Effects of Air Pollution in Hong Kong. Environmental Health Perspectives. 2008;117(2):248–53. 10.1289/ehp.11605 19270795 PMC2649227

[64] Nobusawa E, Sato K. Comparison of the mutation rates of human influenza A and B viruses. Journal of Virology. 2006;80(7):3675–8. 16537638 10.1128/JVI.80.7.3675-3678.2006 PMC1440390

[65] WHO. Recommended composition of influenza virus vaccines for use in the 2015 southern hemisphere influenza season (Table 2) 2014 [1 October 2014]. Available from: http://www.who.int/influenza/vaccines/virus/recommendations/201409_recommendation.pdf. 25313423

