# Supplementary material for: Epidemiological and Evolutionary Dynamics of Influenza B Viruses in Malaysia, 2012-2014
Source: PLoS One. 2015 Aug 27;10(8):e0136254. doi: 10.1371/journal.pone.0136254 (PMC4552379; doi:10.1371/journal.pone.0136254)
Supplement: S1 Fig — (PDF) [file pone.0136254.s001.pdf]

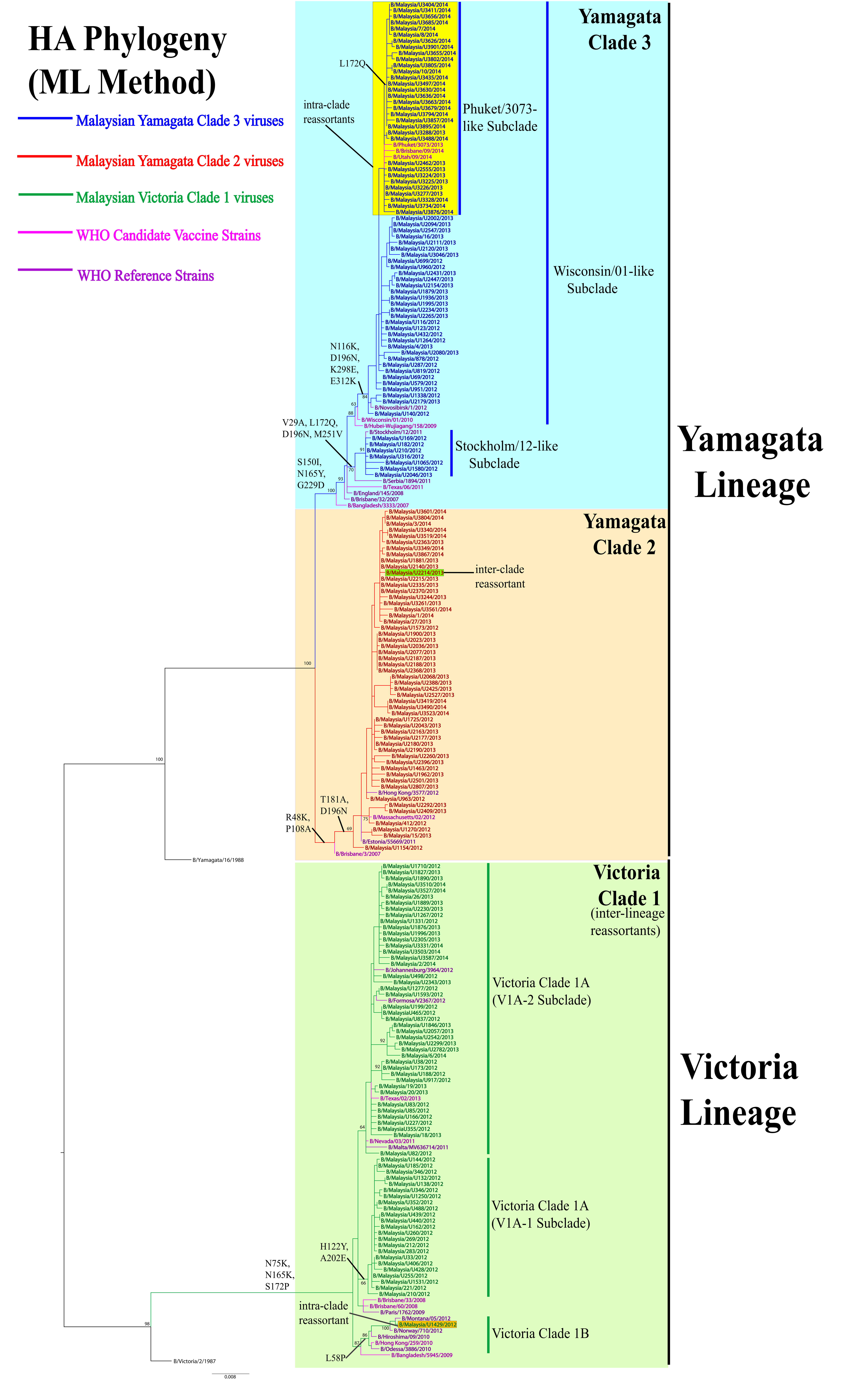
HA Phylogeny
(ML Method)
Malaysian Yamagata Clade 3 viruses
Malaysian Yamagata Clade 2 viruses
Malaysian Victoria Clade 1 viruses
WHO Candidate Vaccine Strains
WHO Reference Strains
Yamagata Clade 3
Phuket/3073-like Subclade
Wisconsin/01-like Subclade
Stockholm/12-like Subclade
Yamagata Lineage
Yamagata Clade 2
Victoria Clade 1 (inter-lineage reassortants)
Victoria Clade 1A (V1A-2 Subclade)
Victoria Clade 1A (V1A-1 Subclade)
Victoria Clade 1B
Victoria Lineage
L172Q
intra-clade reassortants
N116K, D196N, K298E, E312K
V29A, L172Q, D196N, M251V
S150I, N165Y, G229D
inter-clade reassortant
T181A, D196N
R48K, P108A
N75K, N165K, S172P
H122Y, A202E
intra-clade reassortant
L58P
B/Malaysia/U3404/2014
B/Malaysia/U3411/2014
B/Malaysia/U3656/2014
B/Malaysia/U3685/2014
B/Malaysia/7/2014
B/Malaysia/8/2014
B/Malaysia/U3626/2014
B/Malaysia/U3901/2014
B/Malaysia/U3655/2014
B/Malaysia/U3802/2014
B/Malaysia/U3805/2014
B/Malaysia/10/2014
B/Malaysia/U3435/2014
B/Malaysia/U3497/2014
B/Malaysia/U3630/2014
B/Malaysia/U3636/2014
B/Malaysia/U3663/2014
B/Malaysia/U3679/2014
B/Malaysia/U3794/2014
B/Malaysia/U3857/2014
B/Malaysia/U3895/2014
B/Malaysia/U3288/2013
B/Malaysia/U3488/2014
B/Phuket/3073/2013
B/Brisbane/09/2014
B/Utah/09/2014
B/Malaysia/U2462/2013
B/Malaysia/U2555/2013
B/Malaysia/U3224/2013
B/Malaysia/U3225/2013
B/Malaysia/U3226/2013
B/Malaysia/U3277/2013
B/Malaysia/U3328/2014
B/Malaysia/U3734/2014
B/Malaysia/U3876/2014
B/Malaysia/U2002/2013
B/Malaysia/U2094/2013
B/Malaysia/U2547/2013
B/Malaysia/16/2013
B/Malaysia/U2111/2013
B/Malaysia/U2120/2013
B/Malaysia/U3046/2013
B/Malaysia/U699/2012
B/Malaysia/U960/2012
B/Malaysia/U2431/2013
B/Malaysia/U2447/2013
B/Malaysia/U2154/2013
B/Malaysia/U1879/2013
B/Malaysia/U1936/2013
B/Malaysia/U1995/2013
B/Malaysia/U2234/2013
B/Malaysia/U2265/2013
B/Malaysia/U116/2012
B/Malaysia/U123/2012
B/Malaysia/U432/2012
B/Malaysia/U1264/2012
B/Malaysia/4/2013
B/Malaysia/U2080/2013
B/Malaysia/878/2012
B/Malaysia/U287/2012
B/Malaysia/U819/2012
B/Malaysia/U69/2012
B/Malaysia/U579/2012
B/Malaysia/U951/2012
B/Malaysia/U1338/2012
B/Malaysia/U2179/2013
B/Novosibirsk/1/2012
B/Malaysia/U140/2012
B/Wisconsin/01/2010
B/Hubei-Wujiagang/158/2009
B/Stockholm/12/2011
B/Malaysia/U169/2012
B/Malaysia/U182/2012
B/Malaysia/U210/2012
B/Malaysia/U316/2012
B/Malaysia/U1065/2012
B/Malaysia/U1580/2012
B/Malaysia/U2046/2013
B/Serbia/1894/2011
B/Texas/06/2011
B/England/145/2008
B/Brisbane/32/2007
B/Bangladesh/3333/2007
B/Malaysia/U3601/2014
B/Malaysia/U3804/2014
B/Malaysia/3/2014
B/Malaysia/U3340/2014
B/Malaysia/U3519/2014
B/Malaysia/U2363/2013
B/Malaysia/U3349/2014
B/Malaysia/U3867/2014
B/Malaysia/U1881/2013
B/Malaysia/U2140/2013
B/Malaysia/U2214/2013
B/Malaysia/U2215/2013
B/Malaysia/U2335/2013
B/Malaysia/U2370/2013
B/Malaysia/U3244/2013
B/Malaysia/U3261/2013
B/Malaysia/U3561/2014
B/Malaysia/1/2014
B/Malaysia/27/2013
B/Malaysia/U1573/2012
B/Malaysia/U1900/2013
B/Malaysia/U2023/2013
B/Malaysia/U2036/2013
B/Malaysia/U2077/2013
B/Malaysia/U2187/2013
B/Malaysia/U2188/2013
B/Malaysia/U2368/2013
B/Malaysia/U2068/2013
B/Malaysia/U2388/2013
B/Malaysia/U2425/2013
B/Malaysia/U2527/2013
B/Malaysia/U3419/2014
B/Malaysia/U3490/2014
B/Malaysia/U3523/2014
B/Malaysia/U1725/2012
B/Malaysia/U2043/2013
B/Malaysia/U2163/2013
B/Malaysia/U2177/2013
B/Malaysia/U2180/2013
B/Malaysia/U2190/2013
B/Malaysia/U2260/2013
B/Malaysia/U2396/2013
B/Malaysia/U1463/2012
B/Malaysia/U1962/2013
B/Malaysia/U2501/2013
B/Malaysia/U2807/2013
B/Hong Kong/3577/2012
B/Malaysia/U963/2012
B/Malaysia/U2292/2013
B/Malaysia/U2409/2013
B/Massachusetts/02/2012
B/Malaysia/412/2012
B/Malaysia/U1270/2012
B/Malaysia/15/2013
B/Estonia/55669/2011
B/Malaysia/U1154/2012
B/Brisbane/3/2007
B/Yamagata/16/1988
B/Malaysia/U1710/2012
B/Malaysia/U1827/2013
B/Malaysia/U1890/2013
B/Malaysia/U3510/2014
B/Malaysia/U3527/2014
B/Malaysia/26/2013
B/Malaysia/U1889/2013
B/Malaysia/U2230/2013
B/Malaysia/U1267/2012
B/Malaysia/U1331/2012
B/Malaysia/U1876/2013
B/Malaysia/U1996/2013
B/Malaysia/U2305/2013
B/Malaysia/U3331/2014
B/Malaysia/U3503/2014
B/Malaysia/U3587/2014
B/Malaysia/2/2014
B/Johannesburg/3964/2012
B/Malaysia/U498/2012
B/Malaysia/U2343/2013
B/Malaysia/U1277/2012
B/Malaysia/U1593/2012
B/Formosa/V2367/2012
B/Malaysia/U199/2012
B/MalaysiaU465/2012
B/Malaysia/U837/2012
B/Malaysia/U1846/2013
B/Malaysia/U2057/2013
B/Malaysia/U2542/2013
B/Malaysia/U2299/2013
B/Malaysia/U2782/2013
B/Malaysia/6/2014
B/Malaysia/U38/2012
B/Malaysia/U173/2012
B/Malaysia/U188/2012
B/Malaysia/U917/2012
B/Malaysia/19/2013
B/Malaysia/20/2013
B/Texas/02/2013
B/Malaysia/U83/2012
B/Malaysia/U85/2012
B/Malaysia/U166/2012
B/Malaysia/U227/2012
B/MalaysiaU355/2012
B/Malaysia/18/2013
B/Nevada/03/2011
B/Malta/MV636714/2011
B/Malaysia/U82/2012
B/Malaysia/U144/2012
B/Malaysia/U185/2012
B/Malaysia/346/2012
B/Malaysia/U132/2012
B/Malaysia/U138/2012
B/Malaysia/U346/2012
B/Malaysia/U1250/2012
B/Malaysia/U352/2012
B/Malaysia/U488/2012
B/Malaysia/U439/2012
B/Malaysia/U440/2012
B/Malaysia/U162/2012
B/Malaysia/U260/2012
B/Malaysia/269/2012
B/Malaysia/212/2012
B/Malaysia/283/2012
B/Malaysia/U33/2012
B/Malaysia/U406/2012
B/Malaysia/U428/2012
B/Malaysia/U255/2012
B/Malaysia/U1531/2012
B/Malaysia/221/2012
B/Malaysia/210/2012
B/Brisbane/33/2008
B/Brisbane/60/2008
B/Paris/1762/2009
B/Montana/05/2012
B/Malaysia/U1429/2012
B/Norway/710/2012
B/Hiroshima/09/2010
B/Hong Kong/259/2010
B/Odessa/3886/2010
B/Bangladesh/5945/2009
B/Victoria/2/1987
0.008
