# Supplementary material for: Epidemiological and Evolutionary Dynamics of Influenza B Viruses in Malaysia, 2012-2014
Source: PLoS One. 2015 Aug 27;10(8):e0136254. doi: 10.1371/journal.pone.0136254 (PMC4552379; doi:10.1371/journal.pone.0136254)
Supplement: S2 Fig — (PDF) [file pone.0136254.s002.pdf]

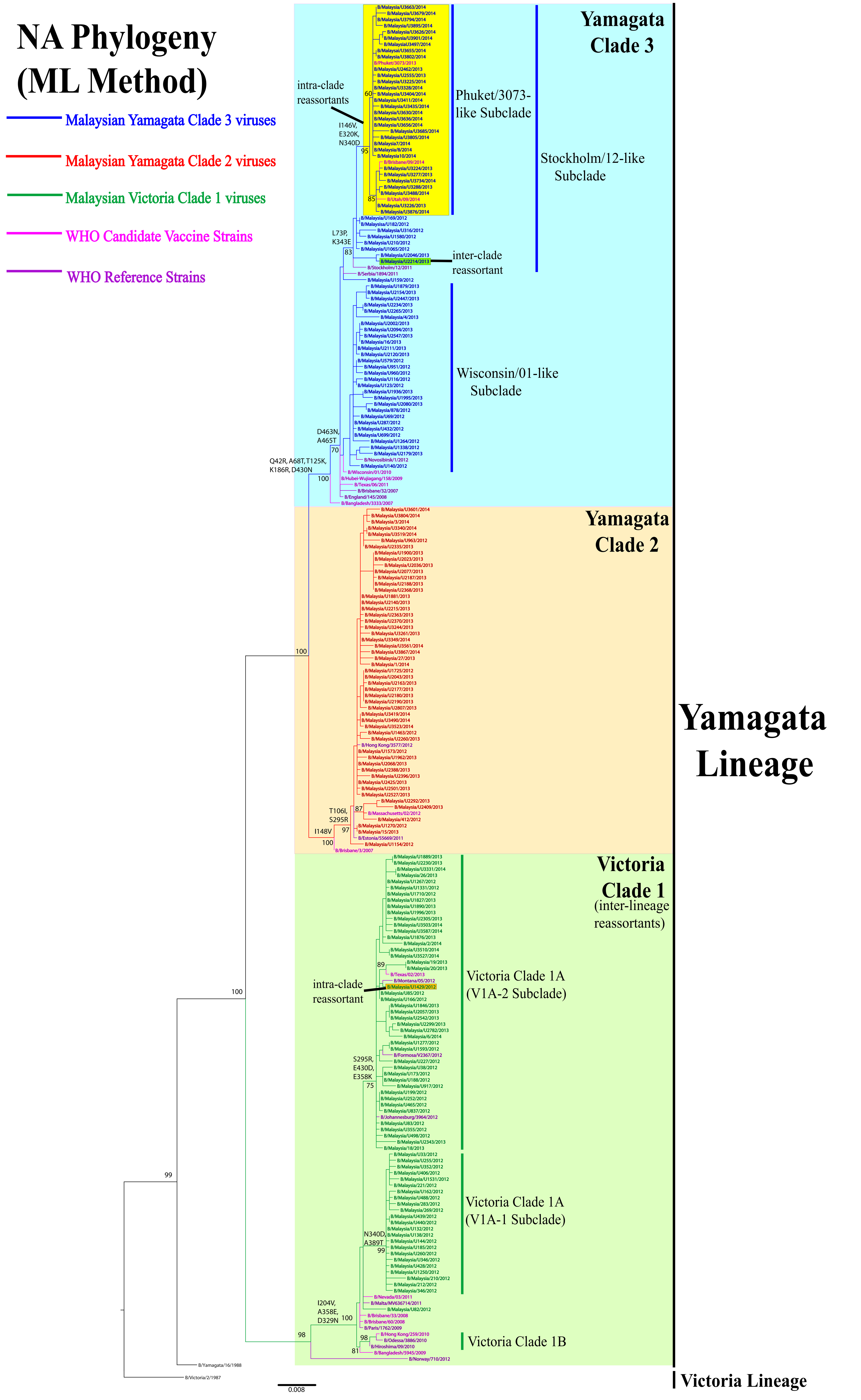

NA Phylogeny
(ML Method)
Malaysian Yamagata Clade 3 viruses
Malaysian Yamagata Clade 2 viruses
Malaysian Victoria Clade 1 viruses
WHO Candidate Vaccine Strains
WHO Reference Strains
Yamagata Clade 3
Phuket/3073-like Subclade
Stockholm/12-like Subclade
Wisconsin/01-like Subclade
intra-clade reassortants
I146V, E320K, N340D
inter-clade reassortant
L73P, K343E
D463N, A465T
Q42R, A68T, T125K, K186R, D430N
Yamagata Clade 2
T106I, S295R
I148V
Yamagata Lineage
Victoria Clade 1
(inter-lineage reassortants)
Victoria Clade 1A (V1A-2 Subclade)
intra-clade reassortant
S295R, E430D, E358K
Victoria Clade 1A (V1A-1 Subclade)
N340D, A389T
I204V, A358E, D329N
Victoria Clade 1B
B/Yamagata/16/1988
B/Victoria/2/1987
Victoria Lineage
0.008
