# Supplementary material for: Epidemiological and Evolutionary Dynamics of Influenza B Viruses in Malaysia, 2012-2014
Source: PLoS One. 2015 Aug 27;10(8):e0136254. doi: 10.1371/journal.pone.0136254 (PMC4552379; doi:10.1371/journal.pone.0136254)
Supplement: S1 Table — Source: London WHO Collaborating Centre, May 2011; WHO information for molecular diagnosis of influenza virus. (PDF) [file pone.0136254.s007.pdf]

S1 Table. Primer Sets, Reagents and Thermocycling Conditions used for two-step Reverse-Transcription Polymerase Chain Reaction (RT-PCR) for Amplification of Influenza B HA and NA genes

A. Primer sets

Gene fragment	Primer	Sequence (5' – 3')	PCR Product Size (bp)
All genes	Buni11W	AGCAGAAGCGS	-
HA-5'	BHAF1u	AGCAGAAGCAGAGCATTTTCTAATATC	1361
	BHAR1341	TTCGTTGTGGAGTTCATCCAT	
HA-3'	BHAF458	AGAAAAGGCACCAGGAGGACCCTA	1391
	BHA2R1	GTAATGGTAACAAGCAAACAAGCA	
NA-5'	BNAF1u	AGCAGAAGCAGAGCATCTTCTCA	1130
	BNAR2	GATGGACAAATCCTCCCTTGATGC	
NA-3'	BNAF2	GCACTCCTAATTAGCCCTCATAGA	1182
	BNAR1487	TAAGGACAATTGTTCAAAC	

Source: London WHO Collaborating Centre, May 2011; WHO information for molecular diagnosis of influenza virus

B. Reagents and their volumes used for reverse transcription (RT) step.

Reagent	Vol. (1X), µl
Universal Primer (Buni11W)	4.5
Template (RNA)	10.0
Water (Molecular Grade)	12.6
5X First-Strand Buffer*	8.0
0.1M DTT*	2.0
100mM dNTP*	0.9
Superscript™ III Reverse Transcriptase*	2.0
Total	40.0

* Invitrogen™, Life Technologies , USA

C. Thermocycling conditions for reverse transcription (RT) step.

Thermocycling steps	Temperature	Cycle	Time (Min)
Denaturation and primer annealing	Mix primer and RNA. Incubate at 65°C. Then chilled on ice.	1	5
Enzyme activation	25°C	1	5
Extension	50°C	1	60
Enzyme deactivation	70°C	1	15

D. Reagents and their volumes used for polymerase chain reaction (PCR) step.

Reagent	Vol. (1X), µl
10x PCR Buffer (15mM $MgCl_2$)*	5.00
dNTP mix (10mM of each)*	1.00
Primer F (10 µM)	2.00
Primer R (10µM)	2.00
HotStarTaq *Plus* DNA Polymerase (250 Units)*	0.25
RNase-free water	35.75
RT product	4.00
Total	50.00

* QIAGEN®, Germany

E. Thermocycling conditions for polymerase chain reaction (PCR) step.

Thermocycling steps	Temperature (°C)	Cycle	Time (Minute)
Initial activation	95	1	5
Denaturation	94		0.5
Annealing	58	40	1
Extension	68		1.5
Final Extension	68	1	10
