# Supplementary material for: Epidemiological and Evolutionary Dynamics of Influenza B Viruses in Malaysia, 2012-2014
Source: PLoS One. 2015 Aug 27;10(8):e0136254. doi: 10.1371/journal.pone.0136254 (PMC4552379; doi:10.1371/journal.pone.0136254)
Supplement: S2 Table — (PDF) [file pone.0136254.s008.pdf]

S2 Table. Influenza B virus clinical isolates sequenced in this study.

No	Name of Sample	Clade (Subclade)				Lineage	Collection Date	Day(s) after Onset of Disease	Age	Sneezing	Nasal Discharge	Nasal congestion	Headache	Sore throat	Hoarseness of voice	Muscle ache	Cough
		Accession No. (HA)	HA	Accession No. (NA)	NA												
1	B/Malaysia/U33/2012	KR073326	Vic-1A (V1A-1)	KR073494	Vic-1A (V1A-1)	Victoria	29-Feb-12	-	41	Yes	Yes	Yes	Yes	Yes	Yes	Yes	Yes
2	B/Malaysia/U38/2012	KR073327	Vic-1A (V1A-2)	KR073495	Vic-1A (V1A-2)	Victoria	29-Feb-12	-	65	Yes	Yes	Yes	No	No	Yes	Yes	Yes
3	B/Malaysia/U69/2012	KR073328	Yam-3 (Wisconsin/01)	KR073496	Yam-3 (Wisconsin/01)	Yamagata	7-Mar-12	-	13	Yes	Yes	Yes	Yes	No	No	Yes	Yes
4	B/Malaysia/U82/2012	KR073329	Vic-1A (V1A-2)	KR073497	Vic-1A (V1A-2)	Victoria	9-Mar-12	-	65	No	No	Yes	Yes	Yes	Yes	No	Yes
5	B/Malaysia/U83/2012	KR073330	Vic-1A (V1A-2)	KR073498	Vic-1A (V1A-2)	Victoria	9-Mar-12	-	28	Yes	Yes	Yes	Yes	Yes	Yes	Yes	Yes
6	B/Malaysia/U85/2012	KR073331	Vic-1A (V1A-2)	KR073499	Vic-1A (V1A-2)	Victoria	9-Mar-12	-	13	No	No	No	Yes	No	No	No	Yes
7	B/Malaysia/U116/2012	KR073332	Yam-3 (Wisconsin/01)	KR073500	Yam-3 (Wisconsin/01)	Yamagata	14-Mar-12	-	51	Yes	Yes	Yes	Yes	Yes	Yes	Yes	Yes
8	B/Malaysia/U123/2012	KR073333	Yam-3 (Wisconsin/01)	KR073501	Yam-3 (Wisconsin/01)	Yamagata	14-Mar-12	-	51	No	No	Yes	No	Yes	Yes	Yes	Yes
9	B/Malaysia/U132/2012	KR073334	Vic-1A (V1A-1)	KR073502	Vic-1A (V1A-1)	Victoria	16-Mar-12	-	16	Yes	Yes	No	Yes	No	No	No	Yes
10	B/Malaysia/U138/2012	KR073335	Vic-1A (V1A-1)	KR073503	Vic-1A (V1A-1)	Victoria	16-Mar-12	-	52	Yes	Yes	No	Yes	No	Yes	Yes	Yes
11	B/Malaysia/U140/2012	KR073336	Yam-3 (Wisconsin/01)	KR073504	Yam-3 (Wisconsin/01)	Yamagata	19-Mar-12	-	46	Yes	Yes	Yes	Yes	Yes	Yes	Yes	Yes
12	B/Malaysia/U144/2012	KR073337	Vic-1A (V1A-1)	KR073505	Vic-1A (V1A-1)	Victoria	19-Mar-12	-	13	Yes	Yes	Yes	Yes	Yes	Yes	Yes	Yes
13	B/Malaysia/U159/2012	-	-	KR073506	Yam-3 (Stockholm/12)	Yamagata	21-Mar-12	2 weeks	33	Yes	Yes	Yes	Yes	Yes	Yes	Yes	Yes
14	B/Malaysia/U162/2012	KR073338	Vic-1A (V1A-1)	KR073507	Vic-1A (V1A-1)	Victoria	21-Mar-12	3 days	26	Yes	Yes	Yes	Yes	Yes	Yes	Yes	Yes
15	B/Malaysia/U166/2012	KR073339	Vic-1A (V1A-2)	KR073508	Vic-1A (V1A-2)	Victoria	21-Mar-12	2 days	32	Yes	Yes	Yes	No	Yes	No	No	Yes
16	B/Malaysia/U169/2012	KR073340	Yam-3 (Stockholm/12)	KR073509	Yam-3 (Stockholm/12)	Yamagata	23-Mar-12	2 days	45	Yes	Yes	Yes	Yes	Yes	Yes	Yes	Yes
17	B/Malaysia/U173/2012	KR073341	Vic-1A (V1A-2)	KR073510	Vic-1A (V1A-2)	Victoria	23-Mar-12	3 days	27	Yes	Yes	Yes	Yes	Yes	Yes	Yes	Yes
18	B/Malaysia/U182/2012	KR073342	Yam-3 (Stockholm/12)	KR073511	Yam-3 (Stockholm/12)	Yamagata	26-Mar-12	4 days	72	Yes	Yes	Yes	Yes	Yes	Yes	Yes	Yes
19	B/Malaysia/U185/2012	KR073343	Vic-1A (V1A-1)	KR073512	Vic-1A (V1A-1)	Victoria	26-Mar-12	4 days	34	Yes	Yes	Yes	Yes	Yes	Yes	Yes	Yes
20	B/Malaysia/U188/2012	KR073344	Vic-1A (V1A-2)	KR073513	Vic-1A (V1A-2)	Victoria	26-Mar-12	4 days	26	Yes	Yes	Yes	Yes	Yes	Yes	Yes	Yes
21	B/Malaysia/U199/2012	KR073345	Vic-1A (V1A-2)	KR073514	Vic-1A (V1A-2)	Victoria	28-Mar-12	2 days	35	No	No	Yes	Yes	Yes	Yes	Yes	Yes
22	B/Malaysia/U210/2012	KR073346	Yam-3 (Stockholm/12)	KR073515	Yam-3 (Stockholm/12)	Yamagata	28-Mar-12	1 week	11	Yes	No	Yes	No	Yes	Yes	No	Yes
23	B/Malaysia/U227/2012	KR073347	Vic-1A (V1A-2)	KR073516	Vic-1A (V1A-2)	Victoria	31-Mar-12	4 days	24	Yes	Yes	Yes	Yes	Yes	Yes	Yes	Yes
24	B/Malaysia/U252/2012	-	-	KR073517	Vic-1A (V1A-2)	Victoria	4-Apr-12	1 week	35	No	No	Yes	Yes	Yes	Yes	Yes	Yes
25	B/Malaysia/U255/2012	KR073348	Vic-1A (V1A-1)	KR073518	Vic-1A (V1A-1)	Victoria	6-Apr-12	4 days	19	Yes	Yes	Yes	Yes	Yes	Yes	Yes	Yes
26	B/Malaysia/U260/2012	KR073349	Vic-1A (V1A-1)	KR073519	Vic-1A (V1A-1)	Victoria	6-Apr-12	4 days	23	No	No	No	Yes	Yes	Yes	Yes	Yes
27	B/Malaysia/U287/2012	KR073350	Yam-3 (Wisconsin/01)	KR073520	Yam-3 (Wisconsin/01)	Yamagata	13-Apr-12	5 days	38	Yes	Yes	No	Yes	No	Yes	Yes	Yes
28	B/Malaysia/U316/2012	KR073351	Yam-3 (Stockholm/12)	KR073521	Yam-3 (Stockholm/12)	Yamagata	16-Apr-12	4 days	49	Yes	Yes	No	Yes	Yes	No	Yes	Yes
29	B/Malaysia/U346/2012	KR073352	Vic-1A (V1A-1)	KR073522	Vic-1A (V1A-1)	Victoria	20-Apr-12	1 week	54	Yes	Yes	No	Yes	Yes	Yes	Yes	Yes
30	B/Malaysia/U352/2012	KR073353	Vic-1A (V1A-1)	KR073523	Vic-1A (V1A-1)	Victoria	23-Apr-12	4 days	7	Yes	Yes	Yes	No	No	Yes	Yes	Yes
31	B/Malaysia/U355/2012	KR073354	Vic-1A (V1A-2)	KR073524	Vic-1A (V1A-2)	Victoria	23-Apr-12	2 days	14	Yes	Yes	Yes	Yes	Yes	Yes	Yes	Yes
32	B/Malaysia/U406/2012	KR073355	Vic-1A (V1A-1)	KR073525	Vic-1A (V1A-1)	Victoria	30-Apr-12	4 days	58	Yes	Yes	No	No	Yes	Yes	Yes	Yes
33	B/Malaysia/U428/2012	KR073356	Vic-1A (V1A-1)	KR073526	Vic-1A (V1A-1)	Victoria	2-May-12	5 days	17	Yes	Yes	Yes	Yes	Yes	Yes	Yes	Yes
34	B/Malaysia/U432/2012	KR073357	Yam-3 (Wisconsin/01)	KR073527	Yam-3 (Wisconsin/01)	Yamagata	4-May-12	5 days	57	Yes	Yes	Yes	Yes	Yes	Yes	Yes	Yes
35	B/Malaysia/U439/2012	KR073358	Vic-1A (V1A-1)	KR073528	Vic-1A (V1A-1)	Victoria	4-May-12	3 days	20	Yes	Yes	Yes	Yes	Yes	Yes	No	Yes
36	B/Malaysia/U440/2012	KR073359	Vic-1A (V1A-1)	KR073529	Vic-1A (V1A-1)	Victoria	4-May-12	2 days	20	Yes	Yes	Yes	Yes	Yes	Yes	Yes	Yes
37	B/Malaysia/U465/2012	KR073360	Vic-1A (V1A-2)	KR073530	Vic-1A (V1A-2)	Victoria	9-May-12	3 days	30	Yes	Yes	Yes	Yes	Yes	Yes	Yes	Yes
38	B/Malaysia/U488/2012	KR073361	Vic-1A (V1A-1)	KR073531	Vic-1A (V1A-1)	Victoria	11-May-12	1 week	37	Yes	Yes	Yes	No	Yes	Yes	Yes	Yes
39	B/Malaysia/U498/2012	KR073362	Vic-1A (V1A-2)	KR073532	Vic-1A (V1A-2)	Victoria	14-May-12	3 days	37	Yes	Yes	Yes	No	Yes	Yes	Yes	Yes
40	B/Malaysia/U579/2012	KR073363	Yam-3 (Wisconsin/01)	KR073533	Yam-3 (Wisconsin/01)	Yamagata	30-May-12	5 days	68	No	Yes	Yes	No	Yes	No	Yes	Yes
41	B/Malaysia/U699/2012	KR073364	Yam-3 (Wisconsin/01)	KR073534	Yam-3 (Wisconsin/01)	Yamagata	18-Jun-12	2 weeks	72	Yes	Yes	No	No	Yes	Yes	No	Yes
42	B/Malaysia/U819/2012	KR073365	Yam-3 (Wisconsin/01)	-	-	Yamagata	9-Jul-12	2 days	32	No	No	No	Yes	Yes	Yes	Yes	Yes
43	B/Malaysia/U837/2012	KR073366	Vic-1A (V1A-2)	KR073535	Vic-1A (V1A-2)	Victoria	11-Jul-12	1 week	29	Yes	Yes	Yes	Yes	Yes	Yes	Yes	Yes
44	B/Malaysia/U917/2012	KR073367	Vic-1A (V1A-2)	KR073536	Vic-1A (V1A-2)	Victoria	23-Jul-12	10 days	63	No	Yes	Yes	No	Yes	No	No	Yes
45	B/Malaysia/U951/2012	KR073368	Yam-3 (Wisconsin/01)	KR073537	Yam-3 (Wisconsin/01)	Yamagata	3-Aug-12	4 days	23	Yes	Yes	Yes	Yes	Yes	Yes	Yes	Yes
46	B/Malaysia/U960/2012	KR073369	Yam-3 (Wisconsin/01)	KR073538	Yam-3 (Wisconsin/01)	Yamagata	6-Aug-12	3 days	12	Yes	No	Yes	No	No	Yes	No	Yes
47	B/Malaysia/U963/2012	KR073370	Yam-2	KR073539	Yam-2	Yamagata	6-Aug-12	5 days	46	Yes	Yes	No	Yes	Yes	Yes	Yes	Yes
48	B/Malaysia/U1065/2012	KR073371	Yam-3 (Stockholm/12)	KR073540	Yam-3 (Stockholm/12)	Yamagata	29-Aug-12	2 days	13	Yes	Yes	Yes	Yes	Yes	Yes	No	Yes
49	B/Malaysia/U1154/2012	KR073372	Yam-2	KR073541	Yam-2	Yamagata	12-Sep-12	3 days	49	No	Yes	No	Yes	No	Yes	Yes	Yes
50	B/Malaysia/U1250/2012	KR073373	Vic-1A (V1A-1)	KR073542	Vic-1A (V1A-1)	Victoria	4-Oct-12	5 days	24	Yes	Yes	Yes	Yes	Yes	Yes	Yes	Yes
51	B/Malaysia/U1264/2012	KR073374	Yam-3 (Wisconsin/01)	KR073543	Yam-3 (Wisconsin/01)	Yamagata	5-Oct-12	4 days	59	Yes	Yes	No	No	Yes	Yes	Yes	Yes
52	B/Malaysia/U1267/2012	KR073375	Vic-1A (V1A-2)	KR073544	Vic-1A (V1A-2)	Victoria	8-Oct-12	5 days	33	Yes	Yes	Yes	Yes	Yes	Yes	Yes	Yes
53	B/Malaysia/U1270/2012	KR073376	Yam-2	KR073545	Yam-2	Yamagata	8-Oct-12	3 days	57	Yes	Yes	Yes	Yes	No	Yes	Yes	Yes
54	B/Malaysia/U1277/2012	KR073377	Vic-1A (V1A-2)	KR073546	Vic-1A (V1A-2)	Victoria	8-Oct-12	1 week	71	No	No	No	No	Yes	No	Yes	Yes
55	B/Malaysia/U1331/2012	KR073378	Vic-1A (V1A-2)	KR073547	Vic-1A (V1A-2)	Victoria	17-Oct-12	1 day	58	Yes	No	No	No	Yes	Yes	Yes	No
56	B/Malaysia/U1338/2012	KR073379	Yam-3 (Wisconsin/01)	KR073548	Yam-3 (Wisconsin/01)	Yamagata	19-Oct-12	2 days	14	Yes	Yes	Yes	Yes	Yes	No	Yes	Yes
57	B/Malaysia/U1429/2012	KR073380	Vic-1B	KR073549	Vic-1A (V1A-2)	Victoria	9-Nov-12	2 days	34	Yes	Yes	Yes	Yes	Yes	Yes	Yes	Yes
58	B/Malaysia/U1463/2012	KR073381	Yam-2	KR073550	Yam-2	Yamagata	14-Nov-12	2 days	64	No	Yes	No	No	No	No	No	Yes
59	B/Malaysia/U1531/2012	KR073382	Vic-1A (V1A-1)	KR073551	Vic-1A (V1A-1)	Victoria	23-Nov-12	1 week	31	Yes	Yes	No	Yes	Yes	No	Yes	Yes
60	B/Malaysia/U1573/2012	KR073383	Yam-2	KR073552	Yam-2	Yamagata	28-Nov-12	4 days	54	No	Yes	No	Yes	No	Yes	Yes	Yes
61	B/Malaysia/U1580/2012	KR073384	Yam-3 (Stockholm/12)	KR073553	Yam-3 (Stockholm/12)	Yamagata	30-Nov-12	3 days	34	No	Yes	Yes	Yes	Yes	Yes	Yes	Yes
62	B/Malaysia/U1593/2012	KR073385	Vic-1A (V1A-2)	KR073554	Vic-1A (V1A-2)	Victoria	3-Dec-12	2 days	23	Yes	Yes	Yes	Yes	Yes	Yes	Yes	Yes
63	B/Malaysia/U1710/2012	KR073386	Vic-1A (V1A-2)	KR073555	Vic-1A (V1A-2)	Victoria	26-Dec-12	2 days	15	Yes	No	No	No	Yes	No	No	No
64	B/Malaysia/U1725/2012	KR073387	Yam-2	KR073556	Yam-2	Yamagata	28-Dec-12	4 days	22	Yes	Yes	Yes	Yes	Yes	Yes	Yes	No
65	B/Malaysia/U1827/2013	KR073388	Vic-1A (V1A-2)	KR073557	Vic-1A (V1A-2)	Victoria	11-Jan-13	10 days	61	No	No	Yes	No	No	Yes	Yes	Yes
66	B/Malaysia/U1846/2013	KR073389	Vic-1A (V1A-2)	KR073558	Vic-1A (V1A-2)	Victoria	14-Jan-13	3 days	21	Yes	Yes	Yes	Yes	Yes	Yes	Yes	Yes
67	B/Malaysia/U1876/2013	KR073390	Vic-1A (V1A-2)	KR073559	Vic-1A (V1A-2)	Victoria	21-Jan-13	4 days	27	Yes	Yes	Yes	No	Yes	Yes	Yes	Yes
68	B/Malaysia/U1879/2013	KR073391	Yam-3 (Wisconsin/01)	KR073560	Yam-3 (Wisconsin/01)	Yamagata	21-Jan-13	4 days	35	Yes	Yes	Yes	Yes	Yes	Yes	Yes	Yes
69	B/Malaysia/U1881/2013	KR073392	Yam-2	KR073561	Yam-2	Yamagata	21-Jan-13	1 week	58	No	No	Yes	Yes	No	Yes	Yes	Yes
70	B/Malaysia/U1889/2013	KR073393	Vic-1A (V1A-2)	KR073562	Vic-1A (V1A-2)	Victoria	23-Jan-13	2 days	71	Yes	Yes	No	No	Yes	No	No	Yes
71	B/Malaysia/U1890/2013	KR073394	Vic-1A (V1A-2)	KR073563	Vic-1A (V1A-2)	Victoria	23-Jan-13	3 days	27	Yes	Yes	Yes	No	No	Yes	No	Yes
72	B/Malaysia/U1900/2013	KR073395	Yam-2	KR073564	Yam-2	Yamagata	23-Jan-13	5 days	62	Yes	Yes	No	Yes	No	Yes	Yes	Yes
73	B/Malaysia/U1936/2013	KR073396	Yam-3 (Wisconsin/01)	KR073565	Yam-3 (Wisconsin/01)	Yamagata	4-Feb-13	2 days	57	Yes	Yes	Yes	Yes	Yes	Yes	Yes	Yes
74	B/Malaysia/U1962/2013	KR073397	Yam-2	KR073566	Yam-2	Yamagata	15-Feb-13	1 week	37	Yes	Yes	Yes	Yes	Yes	Yes	Yes	Yes
75	B/Malaysia/U1995/2013	KR073398	Yam-3 (Wisconsin/01)	KR073567	Yam-3 (Wisconsin/01)	Yamagata	20-Feb-13	5 days	28	Yes	Yes	No	Yes	Yes	Yes	Yes	Yes
76	B/Malaysia/U1996/2013	KR073399	Vic-1A (V1A-2)	KR073568	Vic-1A (V1A-2)	Victoria	20-Feb-13	2 days	30	No	No	No	No	Yes	No	No	Yes
77	B/Malaysia/U2002/2013	KR073400	Yam-3 (Wisconsin/01)	KR073569	Yam-3 (Wisconsin/01)	Yamagata	20-Feb-13	2 days	39	No	Yes	Yes	Yes	Yes	Yes	Yes	Yes
78	B/Malaysia/U2023/2013	KR073401	Yam-2	KR073570	Yam-2	Yamagata	22-Feb-13	5 days	32	Yes	Yes	Yes	Yes	Yes	Yes	Yes	Yes
79	B/Malaysia/U2036/2013	KR073402	Yam-2	KR073571	Yam-2	Yamagata	25-Feb-13	4 days	15	Yes	Yes	Yes	Yes	No	No	Yes	Yes
80	B/Malaysia/U2043/2013	KR073403	Yam-2	KR073572	Yam-2	Yamagata	25-Feb-13	2 days	56	Yes	Yes	Yes	Yes	Yes	Yes	Yes	Yes
81	B/Malaysia/U2046/2013	KR073404	Yam-3 (Stockholm/12)	KR073573	Yam-3 (Stockholm/12)	Yamagata	27-Feb-13	1 week	71	No	No	No	No	No	No	Yes	Yes
82	B/Malaysia/U2057/2013	KR073405	Vic-1A (V1A-2)	KR073574	Vic-1A (V1A-2)	Victoria	27-Feb-13	1 week	24	Yes	Yes	Yes	Yes	Yes	No	Yes	Yes
83	B/Malaysia/U2068/2013	KR073406	Yam-2	KR073575	Yam-2	Yamagata	1-Mar-13	4 days	41	No	Yes	Yes	Yes	Yes	Yes	Yes	Yes
84	B/Malaysia/U2077/2013	KR073407	Yam-2	KR073576	Yam-2	Yamagata	4-Mar-13	5 days	33	Yes	No	Yes	Yes	Yes	Yes	Yes	Yes
85	B/Malaysia/U2080/2013	KR073408	Yam-3 (Wisconsin/01)	KR073577	Yam-3 (Wisconsin/01)	Yamagata	6-Mar-13	2 days	47	Yes	No	Yes	Yes	Yes	Yes	Yes	Yes
86	B/Malaysia/U2094/2013	KR073409	Yam-3 (Wisconsin/01)	KR073578	Yam-3 (Wisconsin/01)	Yamagata	6-Mar-13	2 days	37	Yes	Yes	Yes	Yes	Yes	No	No	Yes
87	B/Malaysia/U2111/2013	KR073410	Yam-3 (Wisconsin/01)	KR073579	Yam-3 (Wisconsin/01)	Yamagata	11-Mar-13	3 days	20	Yes	Yes	Yes	Yes	Yes	Yes	Yes	Yes
88	B/Malaysia/U2120/2013	KR073411	Yam-3 (Wisconsin/01)	KR073580	Yam-3 (Wisconsin/01)	Yamagata	11-Mar-13	3 days	25	Yes	Yes	Yes	No	No	No	Yes	Yes
89	B/Malaysia/U2140/2013	KR073412	Yam-2	KR073581	Yam-2	Yamagata	15-Mar-13	2 days	56	Yes	No	No	Yes	Yes	No	Yes	Yes
90	B/Malaysia/U2154/2013	KR073413	Yam-3 (Wisconsin/01)	KR073582	Yam-3 (Wisconsin/01)	Yamagata	18-Mar-13	2 days	65	Yes	Yes	Yes	Yes	Yes	Yes	Yes	Yes
91	B/Malaysia/U2163/2013	KR073414	Yam-2	KR073583	Yam-2	Yamagata	20-Mar-13	2 days	69	Yes	Yes	No	Yes	No	No	Yes	Yes
92	B/Malaysia/U2177/2013	KR073415	Yam-2	KR073584	Yam-2	Yamagata	20-Mar-13	1 week	70	Yes	Yes	Yes	Yes	Yes	Yes	Yes	Yes
93	B/Malaysia/U2179/2013	KR073416	Yam-3 (Wisconsin/01)	KR073585	Yam-3 (Wisconsin/01)	Yamagata	22-Mar-13	3 days	21	Yes	Yes	Yes	Yes	Yes	Yes	Yes	Yes
94	B/Malaysia/U2180/2013	KR073417	Yam-2	KR073586	Yam-2	Yamagata	22-Mar-13	3 days	33	Yes	Yes	Yes	Yes	No	Yes	Yes	Yes
95	B/Malaysia/U2187/2013	KR073418	Yam-2	KR073587	Yam-2	Yamagata	22-Mar-13	4 days	15	Yes	No	No	Yes	Yes	Yes	Yes	Yes
96	B/Malaysia/U2188/2013	KR073419	Yam-2	KR073588	Yam-2	Yamagata	22-Mar-13	5 days	55	Yes	Yes	No	Yes	Yes	Yes	Yes	Yes
97	B/Malaysia/U2190/2013	KR073420	Yam-2	KR073589	Yam-2	Yamagata	25-Mar-13	3 days	71	Yes	Yes	No	No	No	Yes	Yes	Yes
98	B/Malaysia/U2214/2013	KR073421	Yam-2	KR073590	Yam-3 (Stockholm/12)	Yamagata	29-Mar-13	4 days	28	No	Yes	Yes	No	Yes	Yes	Yes	Yes
99	B/Malaysia/U2215/2013	KR073422	Yam-2	KR073591	Yam-2	Yamagata	29-Mar-13	2 days	32	No	No	Yes	Yes	No	No	No	Yes
100	B/Malaysia/U2230/2013	KR073423	Vic-1A (V1A-2)	KR073592	Vic-1A (V1A-2)	Victoria	1-Apr-13	6 days	20	Yes	Yes	Yes	No	Yes	Yes	Yes	Yes
101	B/Malaysia/U2234/2013	KR073424	Yam-3 (Wisconsin/01)	KR073593	Yam-3 (Wisconsin/01)	Yamagata	1-Apr-13	3 days	78	Yes	No	No	Yes	No	Yes	Yes	Yes
102	B/Malaysia/U2260/2013	KR073425	Yam-2	KR073594	Yam-2	Yamagata	8-Apr-13	5 days	26	Yes	Yes	Yes	Yes	Yes	Yes	Yes	Yes
103	B/Malaysia/U2265/2013	KR073426	Yam-3 (Wisconsin/01)	KR073595	Yam-3 (Wisconsin/01)	Yamagata	8-Apr-13	4 days	58	No	No	No	Yes	No	No	Yes	Yes
104	B/Malaysia/U2292/2013	KR073427	Yam-2	KR073596	Yam-2	Yamagata	15-Apr-13	3 days	79	No	Yes	No	No	Yes	Yes	Yes	Yes
105	B/Malaysia/U2299/2013	KR073428	Vic-1A (V1A-2)	KR073597	Vic-1A (V1A-2)	Victoria	17-Apr-13	4 days	21	Yes	Yes	Yes	Yes	No	No	Yes	Yes
106	B/Malaysia/U2305/2013	KR073429	Vic-1A (V1A-2)	KR073598	Vic-1A (V1A-2)	Victoria	17-Apr-13	2 days	26	No	No	Yes	Yes	Yes	Yes	No	Yes
107	B/Malaysia/U2335/2013	KR073430	Yam-2	KR073599	Yam-2	Yamagata	24-Apr-13	4 days	14	Yes	Yes	Yes	Yes	No	Yes	Yes	Yes
108	B/Malaysia/U2343/2013	KR073431	Vic-1A (V1A-2)	KR073600	Vic-1A (V1A-2)	Victoria	26-Apr-13	1 week	31	Yes	Yes	Yes	Yes	Yes	Yes	Yes	Yes
109	B/Malaysia/U2363/2013	KR073432	Yam-2	KR073601	Yam-2	Yamagata	3-May-13	2 days	62	Yes	Yes	Yes	Yes	Yes	Yes	Yes	Yes
110	B/Malaysia/U2368/2013	KR073433	Yam-2	KR073602	Yam-2	Yamagata	3-May-13	5 days	17	Yes	No	No	Yes	Yes	Yes	Yes	Yes
111	B/Malaysia/U2370/2013	KR073434	Yam-2	KR073603	Yam-2	Yamagata	6-May-13	6 days	67	No	No	No	Yes	No	No	Yes	Yes
112	B/Malaysia/U2388/2013	KR073435	Yam-2	KR073604	Yam-2	Yamagata	10-May-13	1 week	30	Yes	Yes	No	Yes	Yes	Yes	Yes	Yes
113	B/Malaysia/U2396/2013	KR073436	Yam-2	KR073605	Yam-2	Yamagata	10-May-13	4 days	47	Yes	Yes	Yes	Yes	Yes	Yes	Yes	Yes
114	B/Malaysia/U2409/2013	KR073437	Yam-2	KR073606	Yam-2	Yamagata	13-May-13	6 days	48	No	Yes	Yes	Yes	Yes	No	Yes	Yes
115	B/Malaysia/U2425/2013	KR073438	Yam-2	KR073607	Yam-2	Yamagata	17-May-13	1 week	37	No	Yes	No	Yes	No	Yes	Yes	Yes
116	B/Malaysia/U2431/2013	KR073439	Yam-3 (Wisconsin/01)	-	-	Yamagata	20-May-13	5 days	37	Yes	Yes	Yes	Yes	No	Yes	No	Yes
117	B/Malaysia/U2447/2013	KR073440	Yam-3 (Wisconsin/01)	KR073608	Yam-3 (Wisconsin/01)	Yamagata	27-May-13	4 days	73	Yes	No	No	No	No	Yes	Yes	Yes
118	B/Malaysia/U2462/2013	KR073441	Yam-3 (Wisconsin/01)	KR073609	Yam-3 (Stockholm/12)	Yamagata	31-May-13	5 days	26	Yes	Yes	Yes	Yes	Yes	Yes	Yes	Yes
119	B/Malaysia/U2501/2013	KR073442	Yam-2	KR073610	Yam-2	Yamagata	12-Jun-13	4 days	26	Yes	Yes	Yes	Yes	Yes	Yes	Yes	Yes
120	B/Malaysia/U2527/2013	KR073443	Yam-2	KR073611	Yam-2	Yamagata	19-Jun-13	2 days	63	Yes	Yes	No	No	No	No	Yes	No
121	B/Malaysia/U2542/2013	KR073444	Vic-1A (V1A-2)	KR073612	Vic-1A (V1A-2)	Victoria	24-Jun-13	5 days	19	Yes	Yes	Yes	Yes	No	Yes	Yes	Yes
122	B/Malaysia/U2547/2013	KR073445	Yam-3 (Wisconsin/01)	KR073613	Yam-3 (Wisconsin/01)	Yamagata	24-Jun-13	2 days	78	Yes	Yes	No	No	No	No	No	Yes
123	B/Malaysia/U2555/2013	KR073446	Yam-3 (Wisconsin/01)	KR073614	Yam-3 (Stockholm/12)	Yamagata	26-Jun-13	4 days	21	Yes	Yes	Yes	Yes	Yes	Yes	No	Yes
124	B/Malaysia/U2782/2013	KR073447	Vic-1A (V1A-2)	KR073615	Vic-1A (V1A-2)	Victoria	11-Sep-13	4 days	17	Yes	No	Yes	Yes	Yes	Yes	Yes	Yes
125	B/Malaysia/U2807/2013	KR073448	Yam-2	KR073616	Yam-2	Yamagata	20-Sep-13	3 days	70	No	No	No	Yes	Yes	Yes	Yes	Yes
126	B/Malaysia/U3046/2013	KR073449	Yam-3 (Wisconsin/01)	-	-	Yamagata	13-Nov-13	1 day	65	Yes	Yes	Yes	Yes	No	Yes	Yes	No
127	B/Malaysia/U3224/2013	KR073450	Yam-3 (Wisconsin/01)	KR073617	Yam-3 (Stockholm/12)	Yamagata	13-Dec-13	5 days	61	Yes	Yes	Yes	Yes	No	Yes	Yes	Yes
128	B/Malaysia/U3225/2013	KR073451	Yam-3 (Wisconsin/01)	KR073618	Yam-3 (Stockholm/12)	Yamagata	13-Dec-13	4 days	51	No	Yes	No	No	Yes	Yes	Yes	Yes
129	B/Malaysia/U3226/2013	KR073452	Yam-3 (Wisconsin/01)	KR073619	Yam-3 (Stockholm/12)	Yamagata	13-Dec-13	2 days	20	Yes	Yes	Yes	Yes	Yes	No	No	Yes
130	B/Malaysia/U3244/2013	KR073453	Yam-2	KR073620	Yam-2	Yamagata	16-Dec-13	1 week	21	Yes	Yes	Yes	No	Yes	Yes	Yes	No
131	B/Malaysia/U3261/2013	KR073454	Yam-2	KR073621	Yam-2	Yamagata	20-Dec-13	5 days	45	Yes	Yes	No	No	No	Yes	Yes	Yes
132	B/Malaysia/U3277/2013	KR073455	Yam-3 (Wisconsin/01)	KR073622	Yam-3 (Stockholm/12)	Yamagata	23-Dec-13	3 days	25	Yes	Yes	No	Yes	Yes	Yes	Yes	Yes
133	B/Malaysia/U3288/2013	KR073456	Yam-3 (Wisconsin/01)	KR073623	Yam-3 (Stockholm/12)	Yamagata	27-Dec-13	4 days	52	No	No	No	Yes	No	No	Yes	Yes
134	B/Malaysia/U3331/2014	KR073457	Yam-3 (Wisconsin/01)	KR073624	Yam-3 (Stockholm/12)	Yamagata	6-Jan-14	2 days	27	No	Yes	Yes	No	Yes	Yes	Yes	Yes
135	B/Malaysia/U3328/2014	KR073458	Vic-1A (V1A-2)	KR073625	Vic-1A (V1A-2)	Victoria	6-Jan-14	3 days	27	Yes	Yes	Yes	Yes	No	Yes	Yes	Yes
136	B/Malaysia/U3340/2014	KR073459	Yam-2	KR073626	Yam-2	Yamagata	8-Jan-14	1 week	67	Yes	Yes	No	Yes	Yes	Yes	Yes	Yes
137	B/Malaysia/U3349/2014	KR073460	Yam-2	KR073627	Yam-2	Yamagata	10-Jan-14	1 week	67	No	Yes	No	Yes	Yes	Yes	Yes	Yes
138	B/Malaysia/U3404/2014	KR073461	Yam-3 (Wisconsin/01)	KR073628	Yam-3 (Stockholm/12)	Yamagata	20-Jan-14	5 days	52	Yes	Yes	Yes	Yes	Yes	Yes	Yes	Yes
139	B/Malaysia/U3411/2014	KR073462	Yam-3 (Wisconsin/01)	KR073629	Yam-3 (Stockholm/12)	Yamagata	24-Jan-14	1 week	62	No	Yes	Yes	Yes	No	Yes	Yes	Yes
140	B/Malaysia/U3419/2014	KR073463	Yam-2	KR073630	Yam-2	Yamagata	24-Jan-14	3 days	49	No	Yes	Yes	Yes	Yes	Yes	Yes	Yes
141	B/Malaysia/U3435/2014	KR073464	Yam-3 (Wisconsin/01)	KR073631	Yam-3 (Stockholm/12)	Yamagata	29-Jan-14	2 days	34	Yes	Yes	Yes	Yes	No	Yes	Yes	Yes
142	B/Malaysia/U3488/2014	KR073465	Yam-3 (Wisconsin/01)	KR073632	Yam-3 (Stockholm/12)	Yamagata	10-Feb-14	4 days	32	Yes	Yes	No	Yes	Yes	Yes	Yes	Yes
143	B/Malaysia/U3490/2014	KR073466	Yam-2	KR073633	Yam-2	Yamagata	10-Feb-14	4 days	64	No	Yes	No	No	Yes	Yes	Yes	Yes
144	B/Malaysia/U3497/2014	KR073467	Yam-3 (Wisconsin/01)	KR073634	Yam-3 (Stockholm/12)	Yamagata	12-Feb-14	3 days	29	Yes	Yes	Yes	Yes	Yes	Yes	Yes	Yes
145	B/Malaysia/U3503/2014	KR073468	Vic-1A (V1A-2)	KR073635	Vic-1A (V1A-2)	Victoria	12-Feb-14	2 days	19	Yes	Yes	Yes	Yes	Yes	No	Yes	Yes
146	B/Malaysia/U3510/2014	KR073469	Vic-1A (V1A-2)	KR073636	Vic-1A (V1A-2)	Victoria	14-Feb-14	10 days	28	Yes	No	Yes	Yes	Yes	No	Yes	Yes
147	B/Malaysia/U3519/2014	KR073470	Yam-2	KR073637	Yam-2	Yamagata	17-Feb-14	3 days	61	Yes	Yes	Yes	Yes	No	Yes	No	Yes
148	B/Malaysia/U3523/2014	KR073471	Yam-2	KR073638	Yam-2	Yamagata	17-Feb-14	1 week	21	Yes	Yes	Yes	No	Yes	Yes	Yes	Yes
149	B/Malaysia/U3527/2014	KR073472	Vic-1A (V1A-2)	KR073639	Vic-1A (V1A-2)	Victoria	17-Feb-14	5 days	54	No	Yes	Yes	Yes	Yes	Yes	Yes	Yes
150	B/Malaysia/U3561/2014	KR073473	Yam-2	KR073640	Yam-2	Yamagata	26-Feb-14	1 week	60	No	Yes	Yes	Yes	Yes	Yes	No	Yes
151	B/Malaysia/U3587/2014	KR073474	Vic-1A (V1A-2)	KR073641	Vic-1A (V1A-2)	Victoria	3-Mar-14	5 days	26	No	Yes	Yes	Yes	Yes	Yes	Yes	Yes
152	B/Malaysia/U3601/2014	KR073475	Yam-2	KR073642	Yam-2	Yamagata	7-Mar-14	4 days	46	Yes	Yes	Yes	Yes	Yes	Yes	Yes	Yes
153	B/Malaysia/U3626/2014	KR073476	Yam-3 (Wisconsin/01)	KR073643	Yam-3 (Stockholm/12)	Yamagata	12-Mar-14	4 days	49	Yes	Yes	No	Yes	No	Yes	No	Yes
154	B/Malaysia/U3630/2014	KR073477	Yam-3 (Wisconsin/01)	KR073644	Yam-3 (Stockholm/12)	Yamagata	12-Mar-14	3 days	47	Yes	No	Yes	Yes	No	Yes	Yes	Yes
155	B/Malaysia/U3636/2014	KR073478	Yam-3 (Wisconsin/01)	KR073645	Yam-3 (Stockholm/12)	Yamagata	12-Mar-14	5 days	25	Yes	Yes	Yes	Yes	No	Yes	Yes	Yes
156	B/Malaysia/U3655/2014	KR073479	Yam-3 (Wisconsin/01)	KR073646	Yam-3 (Stockholm/12)	Yamagata	17-Mar-14	4 days	52	No	No	No	No	Yes	Yes	Yes	Yes
157	B/Malaysia/U3656/2014	KR073480	Yam-3 (Wisconsin/01)	KR073647	Yam-3 (Stockholm/12)	Yamagata	17-Mar-14	4 days	14	Yes	Yes	Yes	Yes	Yes	Yes	Yes	Yes
158	B/Malaysia/U3663/2014	KR073481	Yam-3 (Wisconsin/01)	KR073648	Yam-3 (Stockholm/12)	Yamagata	17-Mar-14	3 days	27	No	Yes	Yes	No	Yes	Yes	No	Yes
159	B/Malaysia/U3679/2014	KR073482	Yam-3 (Wisconsin/01)	KR073649	Yam-3 (Stockholm/12)	Yamagata	21-Mar-14	3 days	45	Yes	No	No	Yes	Yes	Yes	Yes	Yes
160	B/Malaysia/U3685/2014	KR073483	Yam-3 (Wisconsin/01)	KR073650	Yam-3 (Stockholm/12)	Yamagata	21-Mar-14	2 days	35	Yes	Yes	Yes	Yes	No	No	Yes	No
161	B/Malaysia/U3734/2014	KR073484	Yam-3 (Wisconsin/01)	KR073651	Yam-3 (Stockholm/12)	Yamagata	2-Apr-14	2 days	48	Yes	Yes	No	Yes	Yes	Yes	Yes	Yes
162	B/Malaysia/U3794/2014	KR073485	Yam-3 (Wisconsin/01)	KR073652	Yam-3 (Stockholm/12)	Yamagata	21-Apr-14	11 days	61	Yes	Yes	Yes	No	No	Yes	Yes	Yes
163	B/Malaysia/U3802/2014	KR073486	Yam-3 (Wisconsin/01)	KR073653	Yam-3 (Stockholm/12)	Yamagata	21-Apr-14	4 days	49	No	No	Yes	Yes	Yes	Yes	Yes	Yes
164	B/Malaysia/U3804/2014	KR073487	Yam-2	KR073654	Yam-2	Yamagata	21-Apr-14	4 days	41	No	No	No	Yes	No	Yes	No	Yes
165	B/Malaysia/U3805/2014	KR073488	Yam-3 (Wisconsin/01)	KR073655	Yam-3 (Stockholm/12)	Yamagata	23-Apr-14	3 days	22	Yes	Yes	No	Yes	Yes	Yes	Yes	Yes
166	B/Malaysia/U3857/2014	KR073489	Yam-3 (Wisconsin/01)	-	-	Yamagata	5-May-14	3 days	43	Yes	No	Yes	Yes	Yes	Yes	No	Yes
167	B/Malaysia/U3867/2014	KR073490	Yam-2	KR073656	Yam-2	Yamagata	5-May-14	4 days	43	No	No	No	Yes	No	No	Yes	Yes
168	B/Malaysia/U3876/2014	KR073491	Yam-3 (Wisconsin/01)	KR073657	Yam-3 (Stockholm/12)	Yamagata	9-May-14	1 week	23	No	No	Yes	Yes	Yes	Yes	Yes	Yes
169	B/Malaysia/U3895/2014	KR073492	Yam-3 (Wisconsin/01)	KR073658	Yam-3 (Stockholm/12)	Yamagata	14-May-14	4 days	30	Yes	Yes	Yes	Yes	Yes	Yes	Yes	Yes
170	B/Malaysia/U3901/2014	KR073493	Yam-3 (Wisconsin/01)	KR073659	Yam-3 (Stockholm/12)	Yamagata	16-May-14	3 days	53	Yes	Yes	Yes	No	No	Yes	Yes	Yes
