# Supplementary material for: Epidemiological and Evolutionary Dynamics of Influenza B Viruses in Malaysia, 2012-2014
Source: PLoS One. 2015 Aug 27;10(8):e0136254. doi: 10.1371/journal.pone.0136254 (PMC4552379; doi:10.1371/journal.pone.0136254)
Supplement: S3 Table — (PDF) [file pone.0136254.s009.pdf]

S3 Table. Average nucleotide and amino acid sequence homology.

Year	Victoria Clade 1 vs B/Brisbane/60/2008				Yamagata Clade 2 vs B/Massachusetts/2/2012				Yamagata Clade 3 vs B/Wisconsin/1/2010				WHO Vaccine Component (Southern Hemisphere)	
													Yamagata Lineage	Victoria lineage
	HA (n=67)		NA (n=68)		HA (n=53)		NA (n=52)		HA (n=71)		NA (n=69)			
	nt	aa	nt	aa	nt	aa	nt	aa	nt	aa	nt	aa		
2012	99.29%	99.51%	99.30%	99.26%	99.51%	99.73%	99.50%	99.60%	99.32%	99.29%	99.47%	99.68%	-	B/Brisbane/60/2008-like virus
2013	99.14%	99.52%	99.25%	99.01%	99.33%	99.68%	99.45%	99.58%	99.26%	99.33%	99.31%	99.39%	B/Wisconsin/1/2010-like virus	B/Brisbane/60/2008-like virus
2014	99.11%	99.41%	99.23%	98.92%	99.21%	99.54%	99.42%	99.50%	99.34%	99.24%	99.03%	98.71%	B/Massachusetts/2/2012-like virus	B/Brisbane/60/2008-like virus

n: number of Malaysian influenza B viruses ; nt: nucleotide ; aa: amino acid
