# Supplementary material for: Epidemiological and Evolutionary Dynamics of Influenza B Viruses in Malaysia, 2012-2014
Source: PLoS One. 2015 Aug 27;10(8):e0136254. doi: 10.1371/journal.pone.0136254 (PMC4552379; doi:10.1371/journal.pone.0136254)
Supplement: S4 Table — Grey highlight indicates major signature amino acid substitutions. Substitutions are compared with B/Brisbane/60/2008 vaccine strain. (PDF) [file pone.0136254.s010.pdf]

S4 Table. Amino acid substitutions found on the HA protein for all Malaysian Victoria Clade 1 viruses (n=67).

Amino Acid Position	6	50	73	83	102	105	136	137	144	161	169	179	181	184	186	192	195	205	212	214	217	224	250	282	332	338	360	361	408	433	444	493	570	571
HA1 Position (B-vaccine numbering)		35	58	68	87	90	121	122	129	146	154	164	166	169	171	177	180	190	197	199	202	209	235	267	317	323	345	346	393	418	429	478	555	556
HA1 Position (B/Hong Kong/73 numbering)												161	163	166	168	174	177	187	194	196	199	206	232	264	314	320	342	343	390	415	426	475	552	553
HA2 Position																													46	71	82	131	208	209
**B/Brisbane/60/2008_2008-08-04**	V	T	L	G	V	V	T	H	N	I	A	D	N	A	N	V	I	V	D	T	A	K	T	I	A	I	K	E	N	D	E	V	V	V
394363_B/MALAYSIA/210/2012_2012-01-03	.	.	.	.	.	.	.	.	.	V	.	.	.	.	.	.	.	.	N	.	G	.	.	.	.	.	.	.	.	.	.	.	.	.
379599_B/MALAYSIA/212/2012_2012-01-05	.	.	.	.	.	.	.	Y	.	V	.	.	.	.	.	.	.	.	N	N	E	.	.	.	.	.	.	.	.	.	.	.	.	.
379539_B/MALAYSIA/221/2012_2012-01-12	.	.	.	.	.	.	.	Y	.	V	.	.	.	.	.	.	.	.	N	.	E	.	.	.	.	.	.	.	.	.	.	.	.	.
394365_B/MALAYSIA/283/2012_2012-01-15	.	.	.	.	.	.	.	Y	.	V	.	.	.	.	.	.	.	.	N	.	K	.	.	.	.	.	.	.	.	.	.	.	.	.
379548_B/MALAYSIA/269/2012_2012-02-21	.	.	.	.	.	.	.	Y	.	V	.	N	.	.	.	.	.	.	N	.	E	.	.	.	.	.	.	.	.	.	.	.	.	.
B/Malaysia/U33/2012_2012-02-29	.	.	.	.	.	.	.	Y	.	V	.	.	.	.	.	.	.	.	N	.	E	.	.	.	.	.	.	.	.	.	.	.	.	.
379551_B/MALAYSIA/346/2012_2012-03-01	.	.	.	.	.	.	.	Y	.	V	.	.	T	.	.	.	.	.	S	.	E	.	.	.	.	.	.	.	.	.	.	.	.	.
B/Malaysia/U132/2012_2012-03-16	.	.	.	S	.	.	.	Y	.	V	.	.	.	.	.	.	.	.	N	.	E	.	.	.	.	.	.	.	.	.	.	.	.	.
B/Malaysia/U138/2012_2012-03-16	.	.	.	S	.	.	.	Y	.	V	.	.	.	.	.	.	.	.	N	.	E	.	.	.	.	.	.	.	.	.	.	.	.	I
B/Malaysia/U144/2012_2012-03-19	.	.	.	.	.	.	.	Y	.	V	.	.	.	.	.	.	.	.	N	.	E	.	.	.	.	.	.	.	.	.	.	.	.	.
B/Malaysia/U162/2012_2012-03-21	.	.	.	.	.	.	.	Y	.	V	.	.	.	.	.	.	.	.	N	.	E	.	.	.	.	.	.	.	.	G	.	.	.	.
B/Malaysia/U185/2012_2012-03-26	.	.	.	.	.	.	.	Y	.	V	.	.	.	.	.	.	.	.	N	.	E	.	.	.	.	.	.	.	.	.	.	.	.	.
B/Malaysia/U255/2012_2012-04-06	.	.	.	.	.	.	.	Y	.	V	.	.	.	.	.	.	.	.	N	.	E	.	.	.	.	.	.	.	.	.	.	.	.	.
B/Malaysia/U260/2012_2012-04-06	.	.	.	.	.	.	.	Y	.	V	.	.	.	.	.	.	.	.	N	.	E	.	.	.	.	.	.	.	.	.	.	.	I	.
B/Malaysia/U346/2012_2012-04-20	.	.	.	.	.	.	.	Y	.	V	.	.	.	.	.	.	.	.	N	.	E	.	.	.	.	.	.	.	.	.	.	.	.	.
B/Malaysia/U352/2012_2012-04-23	.	.	.	.	.	.	.	Y	.	V	.	.	.	.	.	.	.	.	N	.	E	.	.	.	.	.	.	.	.	.	.	.	.	.
B/Malaysia/U406/2012_2012-04-30	.	.	.	.	.	.	.	Y	.	V	.	.	.	.	.	.	.	.	N	.	E	.	.	.	.	.	.	.	.	.	.	.	.	.
B/Malaysia/U428/2012_2012-05-02	.	.	.	.	.	.	.	Y	.	V	.	.	.	.	.	.	.	.	N	.	E	.	.	.	.	.	.	.	.	.	.	.	.	.
B/Malaysia/U439/2012_2012-05-04	.	.	.	.	.	.	.	Y	.	V	E	.	.	.	.	.	.	.	N	.	E	.	.	.	.	.	.	.	.	.	.	.	.	.
B/Malaysia/U440/2012_2012-05-04	.	.	.	.	.	.	.	Y	.	V	E	.	.	.	.	.	.	.	N	.	E	.	.	.	.	.	.	.	.	.	.	.	.	.
B/Malaysia/U488/2012_2012-05-11	.	.	.	.	.	.	.	Y	.	V	.	.	.	.	.	.	.	.	N	.	E	.	.	.	.	.	.	.	.	.	.	.	.	.
B/Malaysia/U1250/2012_2012-10-04	.	.	.	.	.	.	.	Y	.	V	.	.	.	.	.	.	.	.	N	.	E	.	.	.	.	.	.	.	.	.	.	.	.	.
B/Malaysia/U1531/2012_2012-11-23	.	.	.	.	.	.	.	Y	.	V	.	.	.	.	.	.	.	.	N	.	E	.	.	.	.	.	.	.	.	.	.	.	.	.
B/Malaysia/U1429/2012_2012-11-09	.	.	P	.	I	.	.	.	S	V	.	.	.	.	D	.	.	.	N	.	.	.	.	.	.	.	.	.	.	.	.	.	.	.
B/Malaysia/U82/2012_2012-03-09	.	.	.	.	.	.	A	.	.	V	.	.	.	.	.	.	.	.	N	.	.	.	.	.	.	.	.	.	.	.	.	.	.	.
B/Malaysia/U83/2012_2012-03-09	.	.	.	.	.	.	.	.	.	V	.	.	.	.	.	.	.	.	N	.	.	.	.	.	.	.	.	.	.	.	.	.	.	.
B/Malaysia/U85/2012_2012-03-09	.	.	.	.	.	.	.	.	.	V	.	.	.	.	.	.	.	.	N	.	.	.	.	.	.	.	.	.	.	.	.	.	.	.
B/Malaysia/U166/2012_2012-03-21	.	.	.	.	.	.	.	.	.	V	.	.	.	.	.	.	.	.	N	.	.	.	.	.	.	.	.	.	.	.	.	.	.	.
B/Malaysia/U227/2012_2012-03-31	.	.	.	.	.	.	.	.	.	V	.	.	.	.	.	.	.	.	N	.	.	.	.	.	.	.	.	.	.	.	.	.	.	.
B/Malaysia/U355/2012_2012-04-23	.	.	.	.	.	.	.	.	.	V	.	.	.	.	.	.	.	.	N	.	.	.	.	.	.	.	.	.	.	.	.	.	.	.
B/Malaysia/U1277/2012_2012-10-08	.	.	.	.	.	.	.	.	.	V	.	.	.	E	.	.	.	.	N	.	.	.	.	V	.	.	.	.	.	.	.	.	.	.
B/Malaysia/U1593/2012_2012-12-03	.	.	.	.	.	.	.	.	.	V	.	.	.	E	.	.	.	.	N	.	.	.	.	V	.	.	.	.	.	.	.	.	.	.
466151_B/MALAYSIA/18/2013_2013-03-31	.	.	.	.	.	.	N	.	.	V	.	.	.	.	.	.	.	.	N	I	.	.	.	.	.	.	.	.	.	.	.	.	.	.
466153_B/MALAYSIA/19/2013_2013-03-31	.	.	.	.	.	.	.	.	.	V	.	.	.	.	.	.	.	.	N	.	.	.	.	.	.	.	.	.	.	.	.	.	.	.
466156_B/MALAYSIA/20/2013_2013-03-31	.	.	.	.	.	.	.	.	.	V	.	.	.	.	.	.	.	.	N	N	.	.	.	.	.	.	.	.	.	.	.	.	.	.
B/Malaysia/U2343/2013_2013-04-26	.	.	.	.	.	.	.	.	.	V	.	.	.	.	.	.	.	.	N	.	.	.	.	.	.	.	.	.	.	.	.	.	.	.
B/Malaysia/U1846/2013_2013-01-14	.	.	.	.	.	I	.	.	.	V	E	.	.	.	.	.	.	.	N	.	.	.	.	.	.	.	.	.	D	.	.	.	.	.
B/Malaysia/U2057/2013_2013-02-27	.	.	.	.	.	I	.	.	.	V	E	.	.	.	.	.	.	.	N	.	.	.	.	.	.	.	.	.	D	.	.	.	.	.
541285_B/MALAYSIA/6/2014_2014-02-20	.	.	.	.	.	I	.	.	.	V	E	.	.	.	.	.	.	.	N	.	.	.	.	.	.	.	.	G	D	.	.	.	.	.
B/Malaysia/U2542/2013_2013-06-24	.	.	.	.	.	I	.	.	.	V	E	.	.	.	.	.	.	.	N	.	.	.	.	.	.	.	.	.	D	.	K	.	.	.
B/Malaysia/U2782/2013_2013-09-11	.	.	.	.	.	I	.	.	.	V	E	.	.	.	.	I	.	.	N	.	.	.	.	.	.	.	.	.	D	.	.	.	.	.
B/Malaysia/U2299/2013_2013-04-17	.	.	.	.	.	I	.	.	.	V	E	.	.	.	.	I	.	.	N	.	.	.	.	.	.	.	.	.	D	.	.	.	.	.
B/Malaysia/U498/2012_2012-05-14	.	.	.	.	.	.	.	.	.	V	.	.	.	.	.	.	.	.	N	.	.	N	.	.	.	.	.	.	.	.	.	.	.	.
B/Malaysia/U1267/2012_2012-10-08	.	.	.	.	.	.	.	.	.	V	.	.	.	.	.	.	.	.	N	.	.	N	.	.	.	.	.	.	.	.	.	.	.	.
B/Malaysia/U1331/2012_2012-10-17	.	.	.	.	.	.	.	.	.	V	.	.	.	.	.	.	.	.	N	.	.	N	.	.	.	.	.	.	.	.	.	.	.	.
B/Malaysia/U1710/2012_2012-12-26	.	.	.	.	.	.	.	.	.	V	.	.	.	.	.	.	.	.	N	.	.	N	.	.	.	.	.	.	.	.	.	.	.	.
B/Malaysia/U1827/2013_2013-01-11	.	.	.	.	.	.	.	.	.	V	.	.	.	.	.	.	.	.	N	.	.	N	.	.	.	.	.	.	.	.	.	.	.	.
B/Malaysia/U1876/2013_2013-01-21	.	.	.	.	.	.	.	.	.	V	.	.	.	.	.	.	.	.	N	.	.	N	.	.	.	.	.	.	.	.	.	.	.	.
B/Malaysia/U1889/2013_2013-01-23	.	.	.	.	.	.	.	.	.	V	.	.	.	.	.	.	.	.	N	.	.	N	.	.	.	.	.	.	.	.	.	.	.	.
B/Malaysia/U1890/2013_2013-01-23	.	A	.	.	.	.	.	.	.	V	.	.	.	.	.	.	.	.	N	.	.	N	.	.	.	.	.	.	.	.	.	.	.	.
B/Malaysia/U1996/2013_2013-02-20	.	.	.	.	.	.	.	.	.	V	.	.	.	.	.	.	.	.	N	.	.	N	.	.	.	.	.	.	.	.	.	I	.	.
B/Malaysia/U2230/2013_2013-04-01	.	.	.	.	.	.	.	.	.	V	.	.	.	.	.	.	.	.	N	.	.	N	.	.	.	.	.	.	.	.	.	.	.	.
B/Malaysia/U2305/2013_2013-04-17	.	.	.	.	.	.	.	.	.	V	.	.	.	.	.	.	.	.	N	.	.	N	.	.	.	.	.	.	.	.	.	.	.	.
529380_B/MALAYSIA/26/2013_2013-09-05	.	.	.	.	.	.	.	.	.	V	.	.	.	.	.	.	.	I	N	.	.	N	.	.	.	.	.	.	.	.	.	.	.	.
B/Malaysia/U3331/2014_2014-01-06	.	.	.	.	.	.	.	.	.	V	.	.	.	.	.	.	.	.	N	.	.	N	.	.	.	.	.	.	.	.	.	.	.	.
541304_B/MALAYSIA/2/2014_2014-01-30	.	.	.	.	.	.	.	.	.	V	.	.	.	.	.	.	.	.	N	.	.	N	.	.	.	V	.	.	.	.	.	.	.	.
B/Malaysia/U3503/2014_2014-02-12	.	.	.	.	.	.	.	.	.	V	.	.	.	.	.	.	.	.	N	.	.	N	.	.	.	.	.	.	.	.	.	.	.	.
B/Malaysia/U3510/2014_2014-02-14	A	.	.	.	.	.	.	.	.	V	.	.	.	.	.	.	.	I	N	.	.	N	.	.	.	.	.	.	.	.	.	.	.	.
B/Malaysia/U3527/2014_2014-02-17	A	.	.	.	.	.	.	.	.	V	.	.	.	.	.	.	.	I	N	.	.	N	.	.	.	.	.	.	.	.	.	.	.	.
B/Malaysia/U3587/2014_2014-03-03	.	.	.	.	.	.	.	.	.	V	.	.	.	.	.	.	.	.	N	.	.	N	.	.	T	.	R	.	.	.	.	.	.	.
B/Malaysia/U38/2012_2012-02-29	.	.	.	.	.	.	.	.	.	V	.	.	.	.	S	.	.	.	N	.	.	.	I	.	.	.	.	.	.	.	.	.	.	.
B/Malaysia/U173/2012_2012-03-23	.	.	.	.	.	.	.	.	.	V	.	.	.	.	S	.	.	.	N	.	.	.	.	.	.	.	.	.	.	.	.	.	.	.
B/Malaysia/U188/2012_2012-03-26	.	.	.	.	.	.	.	.	.	V	.	.	.	.	S	.	.	.	N	.	.	.	.	.	.	.	.	.	.	.	.	.	.	.
B/Malaysia/U917/2012_2012-07-23	.	.	.	.	.	.	.	.	.	V	T	.	.	.	S	.	.	.	N	.	.	.	.	.	.	.	.	.	.	.	.	.	.	.
B/Malaysia/U199/2012_2012-03-28	.	.	.	.	.	.	.	.	.	V	.	.	.	.	.	I	V	.	N	.	.	.	.	.	.	.	.	.	.	.	.	.	.	.
B/Malaysia/U465/2012_2012-05-09	.	.	.	.	.	.	.	.	.	V	.	.	.	.	.	I	V	.	N	.	.	.	.	.	.	.	.	.	.	.	.	.	.	.
B/Malaysia/U837/2012_2012-07-11	.	.	.	.	.	.	.	.	.	V	.	.	.	.	.	I	V	.	N	.	.	.	.	.	.	.	.	.	.	.	.	.	.	.

Grey highlight indicates major clade-defining amino acid substitutions. Substitutions are compared with B/Brisbane/60/2008 vaccine strain.
