# Supplementary material for: Epidemiological and Evolutionary Dynamics of Influenza B Viruses in Malaysia, 2012-2014
Source: PLoS One. 2015 Aug 27;10(8):e0136254. doi: 10.1371/journal.pone.0136254 (PMC4552379; doi:10.1371/journal.pone.0136254)
Supplement: S5 Table — Grey highlight indicates major signature amino acid substitutions. Substitutions are compared with B/Massachusetts/02/2012 vaccine strain. (PDF) [file pone.0136254.s011.pdf]

S5 Table. Amino acid substitutions found on the HA protein for all Malaysian Yamagata Clade 2 viruses (n=53).

Amino Acid Position	6	9	88	90	136	139	142	160	169	178	191	193	197	211	217	244	249	276	293	359	393	406	423	427	540	547
HA1 Position (B-vaccine numbering)			73	75	121	124	127	145	154	163	176	178	182	196	202	229	234	261	278	344	378	391	408	412	525	532
HA1 Position (B/Hong Kong/73 numbering)											174	176	180	194	200	227	232	259	276	342	376	389	406	410	523	530
HA2 Position																					32	45	62	66	179	186
**B/Massachusetts/02/2012_2012-03-13**	V	M	V	T	T	V	A	N	A	D	V	Y	E	D	N	G	T	V	R	K	V	I	N	L	D	T
394452_B/MALAYSIA/412/2012_2012-05-25	.	.	.	.	.	.	.	.	.	.	.	.	.	N	.	.	.	.	.	.	.	.	.	.	.	.
B/Malaysia/U963/2012_2012-08-06	.	.	.	.	.	.	.	.	.	.	.	.	.	N	.	.	.	.	.	.	.	.	.	.	.	.
B/Malaysia/U1154/2012_2012-09-12	.	.	.	.	A	.	.	.	.	.	.	.	.	N	.	.	.	.	.	.	.	.	.	.	.	.
B/Malaysia/U1270/2012_2012-10-08	.	.	.	N	.	.	.	.	.	.	I	.	.	N	.	.	.	.	.	.	.	.	.	.	.	.
B/Malaysia/U1463/2012_2012-11-14	.	.	.	.	.	.	.	.	.	.	.	.	.	N	.	.	.	.	.	.	.	.	.	.	.	I
B/Malaysia/U1573/2012_2012-11-28	.	.	.	.	.	.	.	.	.	.	.	.	.	N	.	.	.	.	.	.	.	.	.	.	.	.
B/Malaysia/U1725/2012_2012-12-28	.	.	.	.	.	.	.	.	.	.	.	.	.	N	.	.	.	.	.	.	.	.	.	.	.	.
B/Malaysia/U1900/2013_2013-01-23	.	.	.	.	.	.	.	.	.	.	.	.	.	N	.	.	.	.	.	.	.	.	.	.	.	.
B/Malaysia/U1962/2013_2013-02-15	.	.	.	.	.	.	.	.	.	.	.	.	.	N	.	.	.	.	K	R	.	.	.	.	.	.
B/Malaysia/U2023/2013_2013-02-22	.	.	.	.	.	.	.	.	.	.	.	.	.	N	.	.	.	.	.	.	.	.	.	.	.	.
B/Malaysia/U2036/2013_2013-02-25	.	.	.	.	.	.	.	.	.	.	.	.	.	N	.	.	.	.	.	.	.	.	.	.	.	.
B/Malaysia/U2043/2013_2013-02-25	.	.	.	.	.	.	.	.	.	.	.	.	.	N	.	.	.	.	.	.	.	.	D	M	.	.
B/Malaysia/U2068/2013_2013-03-01	.	.	.	.	.	.	.	.	.	.	.	.	.	N	.	.	.	.	.	.	.	.	.	.	.	.
B/Malaysia/U2077/2013_2013-03-04	.	.	.	.	.	.	.	.	.	.	.	.	.	N	.	.	.	.	.	.	.	.	.	.	.	.
B/Malaysia/U2163/2013_2013-03-20	.	.	.	.	.	.	.	.	.	.	.	.	.	N	.	.	.	A	.	.	.	.	.	.	.	.
B/Malaysia/U2177/2013_2013-03-20	.	.	.	.	.	.	.	.	.	.	.	.	K	N	.	.	.	.	.	.	.	.	.	.	.	.
B/Malaysia/U2180/2013_2013-03-22	.	.	.	.	.	.	.	.	.	.	.	.	.	N	.	.	.	.	.	.	.	.	.	.	.	.
B/Malaysia/U2187/2013_2013-03-22	.	.	.	.	.	.	.	.	.	.	.	.	.	N	.	.	.	.	.	.	.	.	.	.	.	.
B/Malaysia/U2188/2013_2013-03-22	.	.	.	.	.	.	.	.	.	.	.	.	.	N	.	.	.	.	.	.	.	.	.	.	.	.
B/Malaysia/U2190/2013_2013-03-25	.	.	.	.	.	.	.	.	.	.	.	.	.	N	.	.	.	.	.	.	.	.	.	.	.	.
477623_B/MALAYSIA/15/2013_2013-03-28	.	.	.	N	.	.	.	.	.	.	I	.	.	N	.	D	.	.	.	.	.	.	.	.	.	.
B/Malaysia/U2260/2013_2013-04-08	.	.	.	.	.	.	.	.	.	.	.	.	.	N	.	.	.	.	.	.	.	.	.	.	.	.
B/Malaysia/U2292/2013_2013-04-15	.	.	.	.	.	.	.	.	.	.	.	.	.	N	.	D	.	.	.	.	.	.	.	.	.	.
B/Malaysia/U2368/2013_2013-05-03	.	.	.	.	.	.	.	.	.	.	.	.	.	N	.	.	.	.	.	.	.	.	.	.	.	.
B/Malaysia/U2388/2013_2013-05-10	.	.	.	.	.	.	.	.	V	.	.	.	.	N	.	.	.	.	.	.	.	.	.	.	.	.
B/Malaysia/U2396/2013_2013-05-10	.	.	.	.	.	.	V	.	.	.	.	.	.	N	.	.	.	.	.	.	.	.	.	.	.	.
B/Malaysia/U2409/2013_2013-05-13	.	.	.	.	.	.	.	.	.	G	.	.	.	N	.	D	.	.	.	.	.	.	.	.	.	.
B/Malaysia/U2425/2013_2013-05-17	.	.	.	.	.	.	.	.	.	.	.	.	.	N	S	.	.	.	.	.	.	.	.	.	.	.
B/Malaysia/U2501/2013_2013-06-12	.	.	.	.	S	.	.	.	.	.	.	.	.	N	.	.	.	.	.	.	.	.	.	.	.	.
B/Malaysia/U2527/2013_2013-06-19	.	.	.	.	.	.	.	.	.	.	.	.	.	N	.	.	.	.	.	.	.	.	.	.	.	.
B/Malaysia/U2807/2013_2013-09-20	.	.	.	A	.	.	.	.	.	.	.	.	.	N	.	.	.	.	.	.	.	.	.	.	.	.
B/Malaysia/U3419/2014_2014-01-24	.	.	.	.	.	.	.	.	.	.	.	.	.	N	.	.	R	.	.	.	.	.	.	.	.	.
B/Malaysia/U3490/2014_2014-02-10	.	.	.	.	.	.	.	.	.	.	.	.	.	N	.	.	R	.	.	.	.	.	.	.	.	.
B/Malaysia/U3523/2014_2014-02-17	.	.	.	.	.	.	.	.	.	.	.	.	.	N	.	.	R	.	.	.	.	.	.	.	.	.
B/Malaysia/U1881/2013_2013-01-21	I	.	.	.	.	.	.	.	.	.	.	.	.	N	.	.	.	.	.	.	.	.	.	.	.	.
B/Malaysia/U2214/2013_2013-03-29	I	.	L	.	.	.	.	.	.	.	.	.	.	N	.	.	.	.	.	.	.	.	.	.	.	.
B/Malaysia/U2215/2013_2013-03-29	I	.	.	.	.	.	.	.	.	.	.	.	.	N	.	.	.	.	.	.	.	.	.	.	.	.
B/Malaysia/U2140/2013_2013-03-15	I	.	.	.	.	.	.	.	.	.	.	.	.	N	.	.	.	.	.	.	.	.	.	.	.	.
B/Malaysia/U2335/2013_2013-04-24	I	.	.	.	.	.	.	.	.	.	.	.	.	N	.	.	.	.	.	.	.	.	.	.	.	.
B/Malaysia/U2363/2013_2013-05-03	I	.	.	.	.	.	.	.	.	.	.	.	.	N	.	.	.	.	.	.	.	.	.	.	.	.
B/Malaysia/U2370/2013_2013-05-06	I	.	.	.	.	.	.	.	.	.	.	.	.	N	.	.	.	.	.	.	.	.	.	.	.	.
529354_B/MALAYSIA/27/2013_2013-10-22	I	.	.	.	.	.	.	.	.	.	.	.	.	N	.	.	.	.	.	.	.	.	.	.	.	.
B/Malaysia/U3244/2013_2013-12-16	I	.	.	.	.	.	.	.	.	.	.	.	.	N	.	.	.	.	.	.	.	.	.	.	G	.
B/Malaysia/U3261/2013_2013-12-20	T	.	.	.	.	.	.	.	.	.	.	.	.	N	.	.	.	.	.	.	.	.	.	.	.	.
540750_B/MALAYSIA/1/2014_2014-01-05	I	.	.	.	.	.	.	D	.	.	.	.	.	N	.	.	.	.	.	.	.	.	.	.	.	.
B/Malaysia/U3340/2014_2014-01-08	I	.	.	.	.	.	.	.	.	.	.	.	.	N	.	.	.	.	.	.	.	.	.	.	.	.
B/Malaysia/U3349/2014_2014-01-10	I	.	.	.	.	.	.	.	.	.	.	.	.	N	.	.	.	.	.	.	.	.	.	.	.	.
B/Malaysia/U3519/2014_2014-02-17	I	.	.	.	.	.	.	.	.	.	.	.	.	N	.	.	.	.	.	.	.	.	.	.	.	.
B/Malaysia/U3561/2014_2014-02-26	I	T	.	.	.	A	.	.	.	.	.	.	.	N	.	.	.	.	.	.	.	V	.	.	.	.
B/Malaysia/U3867/2014_2014-05-05	I	.	.	.	.	.	.	.	.	.	.	.	.	N	.	.	.	.	.	.	.	.	.	.	.	.
541276_B/MALAYSIA/3/2014_2014-02-09	I	.	.	.	.	.	.	.	.	.	.	N	.	N	.	.	.	.	.	.	.	.	.	.	.	.
B/Malaysia/U3601/2014_2014-03-07	I	.	.	.	.	.	.	.	.	.	.	N	.	N	.	.	.	.	.	.	L	.	.	.	.	.
B/Malaysia/U3804/2014_2014-04-21	I	.	.	.	.	.	.	.	.	.	.	N	.	N	.	.	.	.	.	.	.	.	.	.	.	.

Grey highlight indicates major clade-defining amino acid substitutions. Substitutions are compared with B/Massachusetts/02/2012 vaccine strain.
