# Supplementary material for: Epidemiological and Evolutionary Dynamics of Influenza B Viruses in Malaysia, 2012-2014
Source: PLoS One. 2015 Aug 27;10(8):e0136254. doi: 10.1371/journal.pone.0136254 (PMC4552379; doi:10.1371/journal.pone.0136254)
Supplement: S6 Table — Grey highlight indicates major signature amino acid substitutions. Substitutions are compared with B/Wisconsin/01/2010 vaccine strain. (PDF) [file pone.0136254.s012.pdf]

S6 Table. Amino acid substitutions found on the HA protein for all Malaysian Yamagata Clade 3 viruses (n=71).

Amino Acid Position	11	14	44	86	91	102	125	131	136	138	139	151	165	187	196	197	198	211	217	222	234	247	266	271	272	283	313	327	334	390	426	427	448	481	530	545	572	576	578
HA1 Position (B-vaccine numbering)			29	71	76	87	110	116	121	123	124	136	150	172	181	182	183	196	202	207	219	232	251	256	257	268	298	312	319	375	411	412	433	466	515	530	557	561	563
HA1 Position (B/Hong Kong/73 numbering)														170	179	180	181	194	200	205	217	230	249	254	255	266	296	310	317	373	409	410	431	464	513	528	555	559	561
HA2 Position																														29	65	66	87	120	169	184	211	215	217
**B/Wisconsin/01/2010_2010-02-20**	V	N	V	M	T	V	L	N	T	N	V	R	I	L	T	E	G	D	S	S	V	D	M	K	T	L	K	E	N	A	R	L	L	R	S	N	M	D	V
**B/Stockholm/12/2011_2011-03-28**	.	.	A	.	.	.	.	.	.	.	.	.	.	Q	.	.	.	D	N	.	.	.	V	.	.	.	.	.	.	.	.	.	.	.	.	.	.	.	.
**B/Phuket/3073/2013_2013-11-21**	.	.	.	.	.	.	.	K	.	.	.	.	.	.	.	.	.	D	.	.	.	.	.	.	.	.	E	K	.	.	.	.	.	.	.	.	.	.	.
B/Malaysia/U169/2012_2012-03-23	.	.	A	.	.	.	.	.	.	.	.	.	.	Q	.	.	.	N	N	.	.	.	V	.	.	.	.	.	.	.	.	.	.	.	.	.	.	.	I
B/Malaysia/U182/2012_2012-03-26	.	.	A	.	.	.	.	.	.	.	.	K	.	Q	.	.	.	N	N	.	.	.	V	.	.	.	.	.	.	.	.	.	.	.	.	.	.	.	.
B/Malaysia/U210/2012_2012-03-28	.	.	A	.	.	.	.	.	.	.	.	.	.	Q	.	.	.	N	N	.	.	.	V	.	.	.	.	.	.	.	.	.	.	.	.	.	.	.	.
B/Malaysia/U316/2012_2012-04-16	.	.	A	.	.	.	.	.	.	.	.	.	.	Q	.	.	.	N	N	.	.	.	V	.	.	.	.	.	.	.	.	.	.	.	.	.	.	.	.
B/Malaysia/U1065/2012_2012-08-29	.	.	A	.	.	.	.	.	A	.	.	.	.	Q	.	.	.	N	N	.	.	.	V	.	.	.	.	.	.	.	S	V	.	.	.	.	.	.	.
B/Malaysia/U1580/2012_2012-11-30	.	.	A	.	.	.	.	.	.	.	.	.	.	Q	.	.	.	N	T	.	.	.	V	.	.	.	.	.	.	.	.	.	.	.	.	.	.	.	.
B/Malaysia/U2046/2013_2013-02-27	.	.	A	.	.	.	.	.	.	T	.	.	.	Q	.	.	.	N	N	.	.	.	V	.	.	.	.	.	.	.	.	.	.	.	.	.	.	.	.
B/Malaysia/U69/2012_2012-03-07	.	.	.	.	.	.	F	K	.	.	.	.	V	.	.	.	.	N	.	.	.	.	.	.	.	.	E	K	.	.	.	.	.	.	.	.	.	.	.
B/Malaysia/U116/2012_2012-03-14	.	.	.	.	.	.	.	K	.	.	.	.	.	.	.	.	.	N	.	.	.	.	.	.	.	.	E	K	.	.	.	.	.	.	.	.	.	.	.
B/Malaysia/U123/2012_2012-03-14	.	.	.	.	.	.	.	K	.	.	.	.	.	.	.	.	.	N	.	.	.	.	.	.	.	.	E	K	.	S	.	.	.	.	.	.	.	.	.
B/Malaysia/U140/2012_2012-03-19	.	.	.	.	.	A	.	K	.	.	.	.	.	.	.	.	.	N	.	.	.	.	.	R	.	.	E	K	.	.	.	.	.	.	.	.	.	.	.
B/Malaysia/U287/2012_2012-04-13	.	.	.	.	.	.	.	K	.	.	.	.	.	.	.	.	.	N	.	.	.	.	.	.	.	.	E	K	.	.	.	.	.	.	.	.	.	.	.
B/Malaysia/U432/2012_2012-05-04	.	.	.	.	.	.	.	K	.	.	.	.	.	.	.	.	.	N	.	.	.	.	.	.	.	.	E	K	.	.	.	.	.	.	.	.	.	.	.
B/Malaysia/U579/2012_2012-05-30	.	.	.	.	.	.	.	K	.	.	.	.	.	.	.	.	.	N	.	.	.	.	.	.	.	.	E	K	.	.	.	.	.	.	.	.	I	.	.
450338_B/MALAYSIA/878/2012_2012-07-06	.	.	.	.	.	.	.	K	.	.	.	.	.	.	.	.	.	N	.	.	.	.	.	.	.	.	E	K	.	.	.	.	.	.	.	.	.	.	.
B/Malaysia/U819/2012_2012-07-09	A	.	.	.	.	.	.	K	.	.	.	.	.	.	.	.	.	N	.	.	.	.	.	.	.	.	E	K	.	.	.	.	.	.	.	.	.	.	.
B/Malaysia/U951/2012_2012-08-03	.	.	.	.	.	.	.	K	.	.	.	.	.	.	.	.	.	N	.	.	.	.	.	.	.	.	E	K	.	.	.	.	.	.	.	.	.	.	.
B/Malaysia/U1264/2012_2012-10-05	.	.	.	.	I	.	.	K	.	.	.	.	.	.	.	.	.	N	.	.	.	.	.	.	.	.	E	K	.	.	.	.	.	.	.	.	.	.	.
B/Malaysia/U1338/2012_2012-10-19	.	.	.	.	.	.	.	K	.	.	.	.	.	Q	.	.	E	N	.	.	.	.	.	.	.	.	E	K	.	.	.	.	.	.	.	.	.	.	.
B/Malaysia/U1879/2013_2013-01-21	.	.	.	.	.	.	.	K	.	.	.	.	.	.	.	.	.	N	.	.	.	.	V	.	.	.	E	K	.	.	.	.	.	.	.	.	.	.	.
B/Malaysia/U1936/2013_2013-02-04	.	.	.	.	.	.	.	K	.	.	.	.	.	.	.	.	.	N	.	.	.	.	.	.	.	.	E	K	.	.	.	.	.	.	.	.	.	.	.
B/Malaysia/U1995/2013_2013-02-20	.	.	.	.	.	.	.	K	.	.	.	.	.	.	.	.	.	N	.	.	.	.	.	.	.	.	E	K	.	.	.	.	.	.	.	.	.	.	.
466159_B/MALAYSIA/4/2013_2013-02-27	.	.	.	.	.	.	.	K	.	.	.	.	.	.	.	.	.	N	.	.	.	.	.	.	.	.	E	K	.	.	.	.	.	.	.	.	.	.	.
B/Malaysia/U2080/2013_2013-03-06	.	.	.	.	.	.	.	K	.	.	.	.	.	.	.	.	.	N	.	.	.	.	.	.	.	.	E	K	.	.	.	.	.	.	.	.	.	.	.
B/Malaysia/U2179/2013_2013-03-22	.	.	.	.	.	.	.	K	.	.	.	.	.	Q	.	.	.	N	.	.	.	.	.	.	.	.	E	K	.	.	.	.	.	.	.	.	.	.	.
B/Malaysia/U2234/2013_2013-04-01	.	.	.	.	.	.	.	K	.	.	.	.	.	.	.	.	.	N	.	.	.	.	.	.	.	.	E	K	.	.	.	.	.	.	.	.	.	.	.
B/Malaysia/U2265/2013_2013-04-08	.	.	.	.	.	.	.	K	.	.	.	.	.	.	.	.	.	N	.	.	.	.	.	.	.	.	E	K	.	.	.	.	.	.	.	.	.	.	.
B/Malaysia/U2154/2013_2013-03-18	.	.	.	.	.	.	.	K	.	.	.	.	.	.	.	.	.	N	.	P	.	.	V	.	.	.	E	K	.	.	.	.	.	.	.	.	.	.	.
B/Malaysia/U2431/2013_2013-05-20	.	.	.	.	.	.	.	K	.	.	.	.	.	.	.	.	.	N	.	P	.	.	V	.	.	F	E	K	.	.	.	.	.	.	.	.	.	.	.
B/Malaysia/U2447/2013_2013-05-27	.	.	.	.	.	.	.	K	.	.	.	.	.	.	.	.	.	N	.	P	.	.	V	.	.	.	E	K	.	.	.	.	.	.	.	.	.	.	.
B/Malaysia/U2462/2013_2013-05-31	.	.	.	.	.	.	.	K	.	.	.	.	.	.	.	.	.	N	.	.	.	.	.	.	.	.	E	K	.	.	.	.	.	.	.	.	.	.	.
B/Malaysia/U2555/2013_2013-06-26	.	.	.	.	.	.	.	K	.	.	.	.	.	.	.	.	.	N	.	.	.	.	.	.	.	.	E	K	.	.	.	.	.	.	.	.	.	.	.
B/Malaysia/U3224/2013_2013-12-13	.	.	.	.	.	.	.	K	.	.	.	.	.	.	.	.	.	N	.	.	.	.	.	.	.	.	E	K	.	.	.	.	.	.	.	.	.	.	.
B/Malaysia/U3225/2013_2013-12-13	.	.	.	.	.	.	.	K	.	.	.	.	.	.	.	.	.	N	.	.	.	N	.	.	.	.	E	K	.	.	.	.	.	.	.	.	.	.	.
B/Malaysia/U3226/2013_2013-12-13	.	.	.	.	.	.	.	K	.	.	.	.	.	.	.	.	.	N	.	.	.	.	.	.	.	.	E	K	.	.	.	.	.	.	.	.	.	.	.
B/Malaysia/U3277/2013_2013-12-23	.	.	.	.	.	.	.	K	.	.	.	.	.	.	.	.	.	N	.	.	.	.	.	.	.	.	E	K	.	.	.	.	.	.	.	.	.	.	.
B/Malaysia/U3288/2013_2013-12-27	.	.	.	.	.	.	.	K	.	.	.	.	.	.	.	.	.	N	.	.	.	.	.	.	.	.	E	K	.	.	.	.	.	.	.	.	.	.	.
B/Malaysia/U3328/2014_2014-01-06	.	.	.	K	.	.	.	K	.	.	.	.	.	.	.	.	.	N	.	.	.	.	.	.	.	.	E	K	.	.	.	.	.	.	.	.	.	.	.
B/Malaysia/U699/2012_2012-06-18	.	.	.	.	.	.	.	K	A	.	.	.	.	.	.	.	.	N	.	.	.	.	.	.	.	.	E	K	.	.	.	.	.	.	.	.	.	.	.
B/Malaysia/U960/2012_2012-08-06	.	.	.	.	.	.	.	K	A	.	.	.	.	.	.	.	.	N	.	.	.	.	.	.	.	.	E	K	.	.	.	.	.	.	.	.	.	.	.
B/Malaysia/U2002/2013_2013-02-20	.	.	.	.	.	.	.	K	A	.	.	.	.	.	.	.	.	N	.	.	.	.	.	.	.	.	E	K	.	.	.	.	F	.	.	.	.	.	.
B/Malaysia/U2094/2013_2013-03-06	.	.	.	.	.	.	.	K	A	.	.	.	.	.	.	.	.	N	.	.	.	.	.	.	.	.	E	K	.	.	.	.	.	.	.	.	.	.	.
B/Malaysia/U2111/2013_2013-03-11	.	.	.	.	.	.	.	K	A	.	.	.	.	.	.	.	.	N	.	.	.	.	.	.	.	.	E	K	.	.	.	.	.	.	.	.	.	N	.
B/Malaysia/U2120/2013_2013-03-11	.	.	.	.	.	.	.	K	A	.	.	.	.	.	.	.	.	N	.	.	.	.	.	.	.	.	E	K	.	.	.	.	.	.	.	.	.	.	.
477626_B/MALAYSIA/16/2013_2013-03-21	.	.	.	.	.	.	.	K	A	.	.	.	.	.	.	.	.	N	.	.	.	.	.	.	.	.	E	K	.	.	.	.	.	.	.	.	.	.	.
B/Malaysia/U2547/2013_2013-06-24	.	.	.	.	.	.	.	K	A	.	.	.	.	.	.	.	.	N	.	.	.	.	.	.	.	.	E	K	.	.	.	.	.	.	.	.	.	.	.
B/Malaysia/U3046/2013_2013-11-13	.	.	.	.	.	.	.	K	A	.	.	.	.	.	A	.	.	N	.	.	.	.	.	.	.	.	E	K	.	.	.	.	.	.	L	.	.	.	.
B/Malaysia/U3488/2014_2014-02-10	.	.	.	.	.	.	.	K	.	.	.	.	.	.	.	.	.	N	.	.	.	.	.	.	.	.	E	K	.	.	.	.	.	.	.	.	.	.	.
B/Malaysia/U3734/2014_2014-04-02	.	S	.	.	.	.	.	K	.	.	.	.	.	.	.	.	.	N	.	.	.	.	.	.	.	.	E	K	.	.	.	.	.	.	.	.	.	.	.
B/Malaysia/U3876/2014_2014-05-09	.	.	.	.	.	.	.	K	.	.	.	.	.	.	.	.	.	N	.	.	.	.	.	.	.	.	E	K	S	.	.	.	.	.	.	.	.	.	.
B/Malaysia/U3404/2014_2014-01-20	.	.	.	.	.	.	.	K	.	.	.	.	.	Q	.	.	.	N	.	.	.	.	.	.	.	.	E	K	.	.	.	.	.	.	.	.	.	.	.
B/Malaysia/U3411/2014_2014-01-24	.	.	.	.	.	.	.	K	.	.	.	.	.	Q	.	.	.	N	.	.	.	.	.	.	.	.	E	K	.	.	.	.	.	.	.	.	.	.	.
B/Malaysia/U3435/2014_2014-01-29	.	.	.	.	.	.	.	K	.	.	.	.	.	Q	.	.	.	N	.	.	.	.	.	.	.	.	E	K	.	.	.	.	.	.	.	.	.	.	.
B/Malaysia/U3497/2014_2014-02-12	.	.	.	.	.	.	.	K	.	.	.	.	.	Q	.	.	.	N	.	.	.	.	.	.	.	.	E	K	.	.	.	.	.	.	.	.	.	.	.
541232_B/MALAYSIA/7/2014_2014-02-20	.	.	.	.	.	.	.	K	.	.	.	.	.	Q	.	.	.	N	.	.	.	.	.	.	.	.	E	K	.	.	.	.	.	.	.	.	.	.	.
B/Malaysia/U3626/2014_2014-03-12	.	.	.	.	.	.	.	K	.	.	A	.	.	Q	.	.	.	N	.	.	.	.	.	.	.	.	E	K	.	.	.	.	.	.	.	.	.	.	.
B/Malaysia/U3630/2014_2014-03-12	.	.	.	.	.	.	.	K	.	.	.	.	.	Q	.	.	.	N	.	.	.	.	.	.	.	.	E	K	.	.	.	.	.	.	.	.	.	.	.
B/Malaysia/U3636/2014_2014-03-12	.	.	.	.	.	.	.	K	.	.	.	.	.	Q	.	.	.	N	.	.	.	.	.	.	.	.	E	K	.	.	.	.	.	.	.	.	.	.	.
B/Malaysia/U3655/2014_2014-03-17	.	.	.	.	.	.	.	K	.	.	.	.	.	Q	.	.	.	N	.	.	.	.	.	.	.	.	E	K	.	.	.	.	.	.	.	.	.	.	.
B/Malaysia/U3656/2014_2014-03-17	.	.	.	.	.	.	.	K	.	.	.	.	.	Q	.	.	.	N	.	.	.	.	.	.	A	.	E	K	.	.	.	.	.	.	.	.	.	.	.
B/Malaysia/U3663/2014_2014-03-17	.	.	.	.	.	.	.	K	.	.	.	.	.	Q	.	.	.	N	.	.	I	.	.	.	.	.	E	K	.	.	.	.	.	K	.	.	.	.	.
B/Malaysia/U3679/2014_2014-03-21	A	.	.	.	.	.	.	K	.	.	.	.	.	Q	.	.	.	N	.	.	.	.	.	.	.	.	E	K	.	.	.	.	.	.	.	.	.	.	.
B/Malaysia/U3685/2014_2014-03-21	.	.	.	.	.	.	.	K	.	.	.	.	.	Q	.	.	.	N	.	.	.	.	.	.	.	.	E	K	.	.	.	.	.	.	.	.	.	.	.
541282_B/MALAYSIA/8/2014_2014-03-26	.	.	.	.	.	.	.	K	.	.	.	.	T	Q	.	.	.	N	.	.	.	.	.	.	.	.	E	K	.	.	.	.	.	.	.	.	.	.	.
B/Malaysia/U3794/2014_2014-04-21	.	.	.	.	.	.	.	K	.	.	.	.	.	Q	.	.	.	N	.	.	.	.	.	.	.	.	E	K	.	.	.	.	.	.	.	D	.	.	.
B/Malaysia/U3802/2014_2014-04-21	.	.	.	.	.	.	.	K	.	.	.	.	.	Q	.	.	.	N	.	.	.	.	.	.	.	.	E	K	.	.	.	.	.	.	.	.	.	.	.
B/Malaysia/U3805/2014_2014-04-23	.	.	.	.	.	.	.	K	.	.	.	.	.	Q	.	.	.	N	.	.	.	.	V	.	.	.	E	K	.	.	.	.	.	.	.	.	.	.	.
B/Malaysia/U3857/2014_2014-05-05	.	.	.	.	.	.	.	K	.	.	.	.	.	Q	.	G	.	N	.	.	.	.	.	.	.	.	E	K	.	.	.	.	.	.	.	.	.	.	.
B/Malaysia/U3895/2014_2014-05-14	.	.	.	.	.	.	.	K	.	.	.	.	.	Q	.	.	.	N	.	.	.	.	.	.	.	.	E	K	.	.	.	.	.	.	.	.	.	.	.
B/Malaysia/U3901/2014_2014-05-16	.	.	.	.	.	.	.	K	.	.	A	.	.	Q	.	.	.	N	.	.	.	.	.	.	.	.	E	K	.	.	.	.	.	.	.	.	.	.	.
551267_B/MALAYSIA/10/2014_2014-06-11	.	.	.	.	.	.	.	K	.	.	.	.	.	Q	.	.	.	N	.	.	.	.	V	.	.	.	E	K	.	.	.	.	.	.	.	.	.	.	.

Grey highlight indicates major clade-defining amino acid substitutions. Substitutions are compared with B/Wisconsin/01/2010 vaccine strain.
