# Supplementary material for: Epidemiological and Evolutionary Dynamics of Influenza B Viruses in Malaysia, 2012-2014
Source: PLoS One. 2015 Aug 27;10(8):e0136254. doi: 10.1371/journal.pone.0136254 (PMC4552379; doi:10.1371/journal.pone.0136254)
Supplement: S7 Table — Grey highlight indicates major signature amino acid substitutions. Substitutions are compared with B/Brisbane/60/2008 vaccine strain. (PDF) [file pone.0136254.s013.pdf]

S7 Table. Amino acid substitutions found on the NA protein for all Malaysian Victoria Clade 1 viruses (n=68).

Amino Acid Position	7	8	15	21	35	36	42	43	44	45	53	54	55	63	65	69	70	72	73	81	95	107	120	129	142	145	151	230	246	249	273	295	340	342	343	345	348	358	360	384	389	390	392	395	417	427	432	452	463
N2 Numbering System	11	12	19	25			42		43	44	52	53	54	62	64	68	69	71	72		100		122	131	144	147	153	231	247	250	274	295	340	341	342	344	349			382	385	386	388	391	415	425	430	455	466
B/Brisbane/60/2008_2008-08-04	Q	T	S	S	D	I	P	T	E	I	D	C	A	V	R	K	G	T	L	T	A	K	I	H	Y	G	N	N	S	S	H	S	N	D	K	S	I	E	K	D	A	D	D	A	D	I	D	G	D
394362_B/MALAYSIA/210/2012_2012-01-03	.	.	.	.	.	.	.	.	.	.	.	.	.	.	.	.	.	.	.	.	.	.	.	.	H	R	.	.	.	.	.	.	D	.	.	.	.	.	.	.	T	.	.	.	.	.	.	.	.
379598_B/MALAYSIA/212/2012_2012-01-05	.	.	.	.	.	.	.	.	.	.	.	.	.	.	.	.	.	.	.	.	.	.	.	.	.	.	.	.	.	N	.	.	D	.	.	.	.	.	.	.	T	.	.	.	N	.	.	.	.
379538_B/MALAYSIA/221/2012_2012-01-12	.	.	.	.	.	.	.	.	.	.	.	.	.	.	.	.	.	.	.	.	.	.	.	.	.	.	.	.	.	.	.	.	D	.	.	.	.	.	.	.	T	.	.	.	.	.	G	.	.
394364_B/MALAYSIA/283/2012_2012-01-15	.	.	.	.	.	.	.	.	.	.	.	.	.	.	.	.	.	.	.	.	.	.	.	.	.	.	.	.	.	.	.	.	D	.	.	.	.	.	E	.	T	.	.	.	.	.	.	.	.
379547_B/MALAYSIA/269/2012_2012-02-21	P	M	.	.	.	.	.	.	.	.	.	.	.	.	.	.	.	.	.	.	.	.	.	.	.	.	.	S	.	.	.	.	D	.	.	.	.	.	.	.	T	.	.	.	.	.	.	.	.
B/Malaysia/U33/2012_2012-02-29	.	.	.	.	.	.	.	.	.	.	.	.	.	.	.	.	.	.	.	.	.	.	.	.	.	.	.	.	.	.	.	.	D	.	.	.	.	.	.	.	T	.	.	.	.	.	.	.	.
379550_B/MALAYSIA/346/2012_2012-03-01	.	.	.	.	.	.	.	.	.	.	.	.	.	.	.	.	E	.	.	.	.	.	.	.	.	.	.	.	.	.	.	.	D	.	.	.	.	.	.	.	T	.	.	.	.	.	.	.	.
B/Malaysia/U132/2012_2012-03-16	.	.	.	.	.	.	.	.	.	.	.	.	.	.	.	.	.	.	.	.	.	.	.	.	.	.	.	.	.	.	.	.	D	.	.	.	.	.	.	.	T	.	.	.	.	.	.	.	.
B/Malaysia/U138/2012_2012-03-16	.	.	.	.	.	.	.	.	.	.	.	.	.	.	.	.	.	.	.	.	.	.	.	.	.	.	.	.	.	.	.	.	D	.	.	.	.	.	.	.	T	.	.	.	.	.	.	.	.
B/Malaysia/U144/2012_2012-03-19	.	.	.	.	.	.	.	.	.	.	.	.	.	.	.	.	.	.	.	.	.	.	.	.	.	.	.	.	.	.	.	.	D	.	.	.	.	.	.	.	T	.	.	.	.	.	.	E	.
B/Malaysia/U162/2012_2012-03-21	.	.	.	.	.	.	.	.	.	.	.	.	.	.	.	.	.	.	.	.	.	.	.	.	.	.	.	.	.	.	.	.	D	.	.	.	.	.	.	.	T	.	.	.	.	.	.	.	.
B/Malaysia/U185/2012_2012-03-26	.	.	.	.	.	.	.	.	.	.	.	.	.	.	.	.	.	.	.	.	.	.	.	.	.	.	.	.	.	.	.	.	D	.	.	.	.	.	.	.	T	.	.	.	.	.	.	.	.
B/Malaysia/U255/2012_2012-04-06	.	.	.	.	.	.	.	.	.	.	.	.	.	.	.	.	.	.	.	.	.	.	.	.	.	.	.	.	.	.	.	.	D	.	.	.	.	.	.	.	T	.	G	.	.	.	.	.	.
B/Malaysia/U260/2012_2012-04-06	.	.	.	.	.	.	.	.	.	.	.	.	.	.	.	.	.	.	.	.	.	.	.	.	.	.	.	.	.	.	.	.	D	.	.	.	.	.	.	N	T	.	.	.	.	.	.	.	.
B/Malaysia/U346/2012_2012-04-20	.	.	.	.	.	.	.	.	.	.	.	.	.	.	.	.	.	.	.	.	.	.	.	Y	.	.	.	.	.	.	.	.	D	.	.	.	.	.	.	.	T	.	.	T	.	.	.	.	.
B/Malaysia/U352/2012_2012-04-23	.	.	.	.	.	.	.	.	.	.	.	.	.	.	.	.	.	.	.	.	.	.	.	.	.	.	.	.	.	.	.	.	D	.	.	.	.	.	.	.	T	.	.	.	.	.	.	.	.
B/Malaysia/U406/2012_2012-04-30	.	.	.	.	.	.	.	.	.	.	.	.	.	.	.	.	.	.	.	.	.	.	.	.	.	.	.	.	.	.	.	.	D	.	.	.	.	.	.	.	T	N	.	.	.	.	.	.	.
B/Malaysia/U428/2012_2012-05-02	.	.	.	.	.	T	.	.	.	.	.	.	.	.	.	.	.	.	.	.	.	.	.	.	.	.	.	.	.	.	.	.	D	.	.	.	.	.	.	.	T	.	.	.	.	.	.	.	.
B/Malaysia/U439/2012_2012-05-04	.	.	.	.	.	.	.	.	.	.	.	.	.	.	.	.	.	.	.	.	.	.	.	.	.	.	.	.	.	.	.	.	D	.	.	.	.	.	.	.	T	.	.	.	.	.	.	.	.
B/Malaysia/U440/2012_2012-05-04	.	.	.	.	.	.	.	.	.	.	.	.	.	.	.	.	.	.	.	.	.	.	.	.	.	.	.	.	.	.	.	.	D	.	.	.	.	.	.	.	T	.	.	.	.	.	.	.	.
B/Malaysia/U488/2012_2012-05-11	.	.	.	L	.	.	.	.	.	.	.	.	.	.	.	.	.	.	.	.	.	.	.	.	.	.	.	.	.	.	.	.	D	.	.	.	.	.	.	.	T	.	.	.	.	.	.	.	.
B/Malaysia/U1250/2012_2012-10-04	.	.	.	.	.	.	.	.	.	.	.	.	.	.	.	.	.	.	.	.	.	.	.	.	.	.	.	.	.	.	.	.	D	.	.	.	.	.	.	.	T	.	.	.	.	.	.	.	.
B/Malaysia/U1531/2012_2012-11-23	.	.	.	.	.	.	.	.	.	.	.	.	.	L	.	.	.	.	.	.	.	.	.	.	.	.	.	.	.	.	.	.	D	.	.	.	.	.	.	.	T	.	.	.	.	.	.	.	.
B/Malaysia/U82/2012_2012-03-09	.	.	.	.	.	.	.	.	.	.	.	.	.	.	.	.	.	I	I	.	.	.	V	.	.	.	.	.	.	.	.	.	D	.	.	.	.	.	.	.	.	.	.	.	.	.	.	.	N
B/Malaysia/U83/2012_2012-03-09	.	.	.	.	.	.	.	.	.	.	.	.	.	.	.	.	.	.	.	.	.	.	.	.	.	.	.	.	.	.	.	R	D	.	.	.	.	K	.	.	.	.	.	.	.	.	.	.	.
B/Malaysia/U85/2012_2012-03-09	.	.	.	.	.	.	.	.	.	.	.	.	.	.	.	.	.	.	.	.	.	.	.	.	.	.	.	.	.	.	.	R	D	.	.	.	.	K	.	.	.	.	.	.	.	.	.	.	.
B/Malaysia/U166/2012_2012-03-21	.	.	.	.	.	.	.	.	.	.	.	.	.	.	.	.	.	.	.	.	.	.	.	.	.	.	.	.	.	.	.	R	D	.	.	.	.	K	.	.	.	.	.	.	.	.	.	.	.
B/Malaysia/U227/2012_2012-03-31	.	.	L	.	.	.	.	.	.	.	.	.	.	.	.	.	.	.	F	.	.	.	.	.	.	.	.	.	T	.	.	R	D	.	.	.	.	K	.	.	.	.	.	.	.	.	.	.	.
B/Malaysia/U355/2012_2012-04-23	.	.	.	.	.	.	.	.	.	.	.	.	.	.	.	.	.	.	.	.	.	.	.	.	.	.	.	.	.	.	.	R	D	.	.	.	.	K	.	.	.	.	.	.	.	.	.	.	.
B/Malaysia/U498/2012_2012-05-14	.	.	.	.	.	.	.	.	.	.	.	.	.	.	.	.	.	.	.	.	.	.	.	.	.	.	.	.	.	.	.	R	D	.	.	.	.	K	.	.	.	.	.	.	.	.	.	.	.
B/Malaysia/U917/2012_2012-07-23	.	.	.	.	.	.	.	.	K	.	G	.	.	.	.	.	.	.	.	.	.	.	.	.	.	.	.	.	.	.	.	R	D	.	.	.	.	K	.	.	.	.	.	.	.	.	.	.	.
B/Malaysia/U1277/2012_2012-10-08	.	.	.	.	.	M	.	.	.	.	.	.	.	.	.	.	.	.	F	.	.	.	.	.	.	.	.	.	.	.	.	R	D	.	.	.	.	K	.	.	.	.	.	.	.	.	.	.	.
B/Malaysia/U1429/2012_2012-11-09	.	.	.	.	.	.	.	.	.	.	.	.	.	.	.	.	.	.	.	.	.	.	.	.	.	.	.	.	.	.	.	R	D	.	.	.	.	K	.	.	.	.	.	.	.	.	.	.	.
B/Malaysia/U1593/2012_2012-12-03	.	.	.	.	.	M	.	.	.	.	.	.	.	.	.	.	.	.	F	.	.	.	.	.	.	.	.	.	.	.	.	R	D	.	.	.	.	K	.	.	.	.	.	.	.	.	.	.	.
B/Malaysia/U1846/2013_2013-01-14	.	.	.	.	.	.	.	.	.	.	.	.	.	.	.	.	.	.	.	.	.	.	.	.	.	.	.	.	.	.	.	R	D	.	.	.	.	K	.	.	.	.	.	.	.	.	.	.	.
B/Malaysia/U1876/2013_2013-01-21	.	.	.	.	.	.	.	.	.	.	.	Y	.	.	.	.	.	.	.	.	.	.	.	.	.	.	.	.	.	.	.	R	D	.	E	.	.	K	.	.	.	.	.	.	.	.	.	.	.
B/Malaysia/U2057/2013_2013-02-27	.	.	.	.	.	.	.	.	.	.	.	.	.	.	.	.	.	.	.	.	.	.	.	.	.	.	.	.	.	.	.	R	D	.	.	.	.	K	.	.	.	.	.	.	.	.	.	.	.
466150_B/MALAYSIA/18/2013_2013-03-31	.	.	.	.	.	.	.	.	.	.	.	.	.	.	.	.	.	.	.	.	.	.	.	.	.	.	.	.	.	.	.	R	D	.	.	.	.	K	.	.	.	.	.	.	.	.	.	.	.
466152_B/MALAYSIA/19/2013_2013-03-31	.	.	.	.	G	.	.	.	.	.	.	.	.	.	.	E	.	.	.	.	.	.	.	.	.	.	.	.	.	.	Y	R	D	.	.	.	.	K	.	.	.	.	.	T	.	.	.	.	.
466155_B/MALAYSIA/20/2013_2013-03-31	.	.	.	.	G	.	.	.	.	.	.	.	.	.	.	E	.	.	.	.	.	.	.	.	.	.	.	.	.	.	Y	R	D	.	.	.	.	K	.	.	.	.	.	T	.	.	.	.	.
B/Malaysia/U2343/2013_2013-04-26	.	.	.	.	.	.	.	.	.	M	.	.	.	.	C	.	.	.	.	.	.	.	.	.	.	.	.	.	.	.	.	R	D	.	.	N	.	K	.	.	.	.	.	.	.	V	.	.	.
B/Malaysia/U2542/2013_2013-06-24	.	.	.	.	.	.	.	.	.	.	.	.	.	.	.	.	.	.	.	.	.	.	.	.	.	.	.	.	.	.	.	R	D	.	.	.	.	K	.	.	.	.	.	.	.	.	.	.	.
B/Malaysia/U1710/2012_2012-12-26	.	.	.	.	.	.	.	.	.	.	.	.	.	.	.	.	.	.	.	.	.	N	.	.	.	.	.	.	.	.	.	R	D	.	.	.	.	K	.	.	.	.	.	.	.	.	.	.	.
B/Malaysia/U2299/2013_2013-04-17	.	.	.	.	.	.	.	.	.	.	.	.	.	.	.	.	.	.	.	.	.	.	.	.	.	.	.	.	.	.	.	R	D	.	.	.	V	K	.	.	.	.	.	V	.	.	.	.	.
B/Malaysia/U2782/2013_2013-09-11	.	.	.	.	.	.	S	.	.	.	.	.	.	.	.	.	.	.	.	.	.	.	.	.	.	.	.	.	.	.	.	R	D	.	.	.	V	K	.	.	.	.	.	.	.	.	.	.	.
541284_B/MALAYSIA/6/2014_2014-02-20	.	.	.	.	.	.	.	.	.	.	.	Y	.	.	H	.	.	.	.	.	.	.	.	.	.	.	.	.	.	.	.	R	D	.	.	.	V	K	.	.	.	.	.	.	.	.	.	.	.
541303_B/MALAYSIA/2/2014_2014-01-30	.	.	.	.	.	.	.	.	.	.	.	.	.	.	.	.	.	.	F	.	.	.	.	.	.	.	T	.	.	.	.	R	D	.	E	.	.	K	.	.	.	.	.	.	.	.	.	.	.
B/Malaysia/U3510/2014_2014-02-14	.	.	.	.	.	.	.	.	.	.	.	.	.	.	.	.	.	.	.	.	.	.	.	.	.	.	.	.	.	.	.	R	D	N	E	.	.	K	.	.	.	.	.	.	.	.	.	.	N
B/Malaysia/U3527/2014_2014-02-17	.	.	.	.	.	.	.	.	.	.	.	.	.	.	.	.	.	.	.	.	.	.	.	.	.	.	.	.	.	.	.	R	D	N	E	.	.	K	.	.	.	.	.	.	.	.	.	.	N
B/Malaysia/U1267/2012_2012-10-08	.	.	.	.	.	.	.	.	.	.	.	.	.	.	.	.	.	.	.	.	.	N	.	.	.	.	.	.	.	.	.	R	D	.	E	.	.	K	.	.	.	.	.	.	.	.	.	.	.
B/Malaysia/U1331/2012_2012-10-17	.	.	.	.	.	.	.	.	.	.	.	.	.	.	.	.	.	.	.	.	.	N	.	.	.	.	.	.	.	.	.	R	D	.	E	.	.	K	.	.	.	.	.	.	.	.	.	.	.
B/Malaysia/U1827/2013_2013-01-11	.	.	.	.	.	.	.	.	.	.	.	.	.	.	.	.	.	.	.	.	.	N	.	.	.	.	.	.	.	.	.	R	D	.	E	.	.	K	.	.	.	.	.	.	.	.	.	.	.
B/Malaysia/U1889/2013_2013-01-23	.	.	.	.	.	.	.	.	.	.	.	.	.	.	.	.	.	.	.	.	.	N	.	.	.	.	.	.	.	.	.	R	D	.	E	.	.	K	.	.	.	.	.	.	.	.	.	.	.
B/Malaysia/U1890/2013_2013-01-23	.	.	.	.	.	.	.	.	.	.	.	.	.	.	.	.	.	.	.	.	.	N	.	.	.	.	.	.	.	.	.	R	D	.	E	.	.	K	.	.	.	.	.	.	.	.	.	.	.
B/Malaysia/U1996/2013_2013-02-20	.	.	.	.	.	.	.	.	.	.	.	.	.	.	.	.	.	.	.	.	.	N	.	.	.	.	.	.	.	.	.	R	D	.	E	.	.	K	.	.	.	.	.	.	.	.	.	.	.
B/Malaysia/U2230/2013_2013-04-01	.	.	.	.	.	.	.	.	.	.	.	.	.	.	.	.	.	.	.	.	.	N	.	.	.	.	.	.	.	.	.	R	D	.	E	.	.	K	.	.	.	.	.	.	.	.	.	.	.
B/Malaysia/U2305/2013_2013-04-17	.	.	.	.	.	.	.	.	.	.	N	.	.	.	.	.	.	.	.	.	.	N	.	.	.	.	.	.	.	.	.	R	D	.	E	.	.	K	.	G	.	.	.	.	.	.	.	.	.
529379_B/MALAYSIA/26/2013_2013-09-05	.	.	.	.	.	.	.	.	.	.	.	.	.	.	.	.	.	.	.	.	.	N	.	.	.	.	.	.	.	.	.	R	D	.	E	.	.	K	.	.	V	.	.	.	.	.	.	.	.
B/Malaysia/U3331/2014_2014-01-06	.	.	.	.	.	.	.	.	.	.	.	.	.	.	.	.	.	.	.	.	.	N	.	.	.	.	.	.	.	.	.	R	D	.	E	.	.	K	.	.	V	.	.	.	.	.	.	.	.
B/Malaysia/U3503/2014_2014-02-12	.	.	.	.	.	.	.	.	.	.	.	.	.	.	.	.	.	.	.	I	.	N	.	.	.	.	.	.	.	.	.	R	D	.	E	.	.	K	.	.	.	.	.	.	.	.	.	.	.
B/Malaysia/U3587/2014_2014-03-03	.	.	.	.	.	.	.	.	.	.	.	.	.	.	.	.	.	.	.	.	.	N	.	.	.	.	.	.	.	.	.	R	D	.	E	.	.	K	.	.	.	.	.	.	.	.	.	.	.
B/Malaysia/U199/2012_2012-03-28	.	.	.	.	.	.	.	K	.	.	.	.	.	.	.	.	.	.	.	.	.	.	.	.	.	.	.	.	.	.	.	R	D	.	.	.	.	K	.	.	.	.	.	.	.	.	.	.	.
B/Malaysia/U252/2012_2012-04-04	.	.	.	.	.	.	.	K	.	.	.	.	.	.	.	.	.	.	.	.	.	.	.	.	.	.	.	.	.	.	.	R	D	.	.	.	.	K	.	.	.	.	.	.	.	.	.	.	.
B/Malaysia/U465/2012_2012-05-09	.	.	.	.	.	.	.	K	.	.	.	.	.	.	.	.	.	.	.	.	.	.	.	.	.	.	.	.	.	.	.	R	D	.	.	.	.	K	.	.	.	.	.	.	.	.	.	.	.
B/Malaysia/U837/2012_2012-07-11	.	.	.	.	.	.	.	K	.	.	.	.	V	.	.	.	.	.	.	.	.	.	.	.	.	.	.	.	.	.	.	R	D	.	.	.	.	K	.	.	.	.	.	.	.	.	.	.	.
B/Malaysia/U38/2012_2012-02-29	.	.	.	.	.	.	.	.	K	.	.	.	.	.	.	.	.	.	.	.	V	.	.	.	.	.	.	.	.	.	.	R	D	.	.	.	.	K	.	.	.	.	.	.	.	.	.	.	.
B/Malaysia/U173/2012_2012-03-23	.	.	.	.	.	.	.	.	K	.	.	.	.	.	.	.	.	.	.	.	.	.	.	.	.	.	.	.	.	.	.	R	D	.	.	.	.	K	.	.	.	.	.	.	.	.	.	.	.
B/Malaysia/U188/2012_2012-03-26	.	.	.	.	.	.	.	.	K	.	.	.	.	.	.	.	.	.	.	.	.	.	.	.	.	.	.	.	.	.	.	R	D	.	.	.	.	K	.	.	.	.	.	.	.	.	.	.	.

Grey highlight indicates major clade-defining amino acid substitutions. Substitutions are compared with B/Brisbane/60/2008 vaccine strain.
