# Supplementary material for: Epidemiological and Evolutionary Dynamics of Influenza B Viruses in Malaysia, 2012-2014
Source: PLoS One. 2015 Aug 27;10(8):e0136254. doi: 10.1371/journal.pone.0136254 (PMC4552379; doi:10.1371/journal.pone.0136254)
Supplement: S8 Table — Grey highlight indicates major signature amino acid substitutions. Substitutions are compared with B/Massachusetts/02/2012 vaccine strain. (PDF) [file pone.0136254.s014.pdf]

S8 Table. Amino acid substitutions found on the NA protein for all Malaysian Yamagata Clade 2 viruses (n=52).

Amino Acid Position	5	15	46	47	49	50	65	67	68	71	73	76	77	88	94	114	120	128	175	198	200	262	295	320	334	345	358	360	381	384	392	402	436	465
N2 Numbering System	9	19	45	46	48	49	64	66	67	70	72	75	76	93	99	116	122	130	176	199	201	263	295	320	334	344			379	382	388	398	434	469
B/Massachusetts/02/2012_2012-03-13	T	S	T	A	I	M	H	A	A	V	L	P	E	P	K	I	I	K	A	S	A	I	R	E	T	S	A	K	V	D	E	S	T	A
394451_B/MALAYSIA/412/2012_2012-05-25	.	.	.	.	.	.	.	.	.	.	.	.	K	.	.	.	.	.	.	N	.	.	.	.	.	.	.	.	.	.	.	.	.	.
B/Malaysia/U2292/2013_2013-04-15	.	.	.	.	.	.	.	.	.	.	.	.	.	.	.	.	.	.	.	.	.	.	.	.	.	.	T	.	.	.	G	.	.	.
B/Malaysia/U2409/2013_2013-05-13	.	.	N	T	.	.	.	.	.	.	.	.	.	.	I	.	.	.	.	.	.	.	.	.	.	.	T	.	.	.	G	.	.	.
B/Malaysia/U1154/2012_2012-09-12	.	.	.	.	.	.	R	.	.	.	.	.	.	.	.	.	.	R	.	.	.	.	.	.	.	.	.	.	.	.	.	.	.	.
B/Malaysia/U1270/2012_2012-10-08	.	.	.	.	.	.	R	.	.	.	.	.	.	.	.	.	.	.	.	.	.	.	.	.	.	.	.	.	.	.	.	.	.	.
B/Malaysia/U1463/2012_2012-11-14	.	.	.	.	.	I	R	.	.	.	.	.	.	.	.	.	.	.	.	.	.	.	.	.	.	.	.	.	.	.	.	.	.	.
B/Malaysia/U1573/2012_2012-11-28	.	.	.	.	.	.	R	.	.	.	.	.	.	.	.	.	.	.	.	.	.	.	.	.	.	.	.	.	.	.	.	.	.	.
B/Malaysia/U1881/2013_2013-01-21	.	.	.	.	.	.	R	.	.	.	.	.	.	.	.	.	.	.	.	.	.	.	.	.	.	.	.	.	.	.	.	.	.	.
B/Malaysia/U1962/2013_2013-02-15	.	.	.	.	.	.	R	.	V	.	.	.	.	.	.	M	.	.	.	.	.	.	.	.	I	.	.	.	.	.	.	.	.	.
B/Malaysia/U2068/2013_2013-03-01	.	.	.	.	.	.	R	.	.	.	.	.	.	.	.	.	.	.	.	.	.	.	.	.	.	.	.	.	.	.	.	.	.	.
B/Malaysia/U2140/2013_2013-03-15	.	.	.	.	.	.	R	.	.	.	.	.	.	.	.	.	.	.	.	.	.	.	.	.	.	.	.	.	.	.	.	.	.	.
B/Malaysia/U1725/2012_2012-12-28	.	.	.	.	T	.	R	.	.	.	.	.	.	.	.	.	.	.	.	.	.	.	.	.	.	.	.	.	.	.	.	.	.	.
B/Malaysia/U2043/2013_2013-02-25	.	.	.	.	T	.	R	.	.	.	.	.	.	.	.	.	.	.	.	.	.	.	.	.	.	.	.	.	.	.	.	.	.	.
B/Malaysia/U2163/2013_2013-03-20	.	L	.	.	T	.	R	.	.	.	.	.	.	.	.	.	.	.	.	.	.	.	.	.	.	.	.	.	.	.	.	.	.	.
B/Malaysia/U2177/2013_2013-03-20	.	.	.	.	T	.	R	.	.	.	.	.	.	.	.	.	.	.	.	.	.	.	.	.	.	.	.	.	.	.	.	.	.	.
B/Malaysia/U2180/2013_2013-03-22	.	.	.	.	T	.	R	.	.	.	.	.	.	.	.	.	.	.	.	.	.	.	.	.	.	.	.	.	.	.	.	.	.	.
B/Malaysia/U2190/2013_2013-03-25	.	.	.	.	T	.	R	.	.	.	.	.	.	.	.	.	.	.	.	.	.	.	.	.	.	.	.	.	.	.	.	.	.	.
B/Malaysia/U2807/2013_2013-09-20	.	.	.	.	T	.	R	.	.	.	.	.	.	.	.	.	.	.	.	.	.	.	.	.	.	.	.	.	.	.	.	.	.	.
477622_B/MALAYSIA/15/2013_2013-03-28	.	.	.	.	.	.	R	.	.	.	.	.	.	.	.	.	.	.	.	.	.	.	.	.	.	.	.	.	.	.	.	.	.	.
B/Malaysia/U2215/2013_2013-03-29	.	.	.	.	.	.	R	.	.	.	.	.	.	.	.	.	.	.	.	.	.	.	.	.	.	.	.	.	.	.	.	.	.	.
B/Malaysia/U2260/2013_2013-04-08	.	.	.	.	.	.	R	.	.	L	.	.	.	.	.	.	.	.	.	.	.	.	.	.	.	.	.	.	.	.	.	P	.	.
B/Malaysia/U2363/2013_2013-05-03	.	.	.	.	.	.	R	.	.	.	.	.	.	.	.	.	.	.	.	.	.	.	.	.	.	.	.	.	.	.	.	.	.	.
B/Malaysia/U1900/2013_2013-01-23	.	.	.	.	.	.	R	.	.	.	.	.	.	.	.	.	.	.	.	.	.	.	.	.	.	.	.	.	.	.	.	.	A	.
B/Malaysia/U2023/2013_2013-02-22	.	.	.	.	.	.	R	.	.	.	.	.	.	.	.	.	.	.	.	.	.	.	.	.	.	.	.	.	.	.	.	.	A	.
B/Malaysia/U2036/2013_2013-02-25	N	.	.	.	.	.	R	.	.	.	.	.	.	.	.	.	.	.	.	.	.	.	.	.	.	.	.	.	.	.	.	.	A	.
B/Malaysia/U2077/2013_2013-03-04	.	.	.	.	.	.	R	.	.	.	.	.	.	.	.	.	.	.	.	.	.	.	.	.	.	.	.	.	.	.	.	.	A	.
B/Malaysia/U2187/2013_2013-03-22	.	.	.	.	.	.	R	.	.	.	.	.	.	.	.	.	.	.	.	.	.	.	.	.	.	G	.	.	.	.	.	.	A	.
B/Malaysia/U2188/2013_2013-03-22	.	.	.	.	.	.	R	.	.	.	.	.	.	.	.	.	.	.	.	.	.	.	.	.	.	.	.	.	.	.	.	.	A	.
B/Malaysia/U2368/2013_2013-05-03	.	.	.	.	.	.	R	.	.	.	.	.	.	.	.	.	.	.	.	.	.	.	.	.	.	.	.	.	.	.	.	.	A	.
B/Malaysia/U2370/2013_2013-05-06	.	.	.	.	.	.	R	.	.	.	.	.	.	.	.	.	.	.	.	.	.	.	.	.	.	.	.	.	.	.	.	.	.	.
B/Malaysia/U2388/2013_2013-05-10	.	.	.	.	.	.	R	.	.	.	.	.	.	.	.	.	.	.	.	N	.	.	.	.	.	.	.	.	.	.	.	.	.	.
B/Malaysia/U2396/2013_2013-05-10	.	.	.	.	.	.	R	.	.	.	.	.	.	.	.	.	.	.	.	.	.	.	.	.	.	.	.	.	.	.	.	.	.	.
B/Malaysia/U2425/2013_2013-05-17	.	.	.	.	.	.	R	.	.	.	.	.	.	.	.	.	.	.	.	.	.	.	.	.	.	.	.	.	.	.	.	.	.	.
B/Malaysia/U2501/2013_2013-06-12	.	.	.	.	.	.	R	.	.	.	.	.	.	.	.	.	.	.	.	.	.	.	.	K	.	.	.	.	.	.	.	.	.	.
B/Malaysia/U2527/2013_2013-06-19	.	.	.	.	.	.	R	.	.	.	.	.	.	.	.	.	.	.	.	.	.	.	.	.	.	.	.	.	.	.	.	.	.	.
529353_B/MALAYSIA/27/2013_2013-10-22	.	.	.	.	.	.	R	.	.	.	.	.	.	.	.	.	.	.	.	.	.	T	.	.	.	.	.	.	.	.	.	.	.	.
B/Malaysia/U3244/2013_2013-12-16	.	.	.	.	.	.	R	.	.	.	.	.	.	.	.	.	.	.	.	.	.	.	.	.	.	.	.	.	.	.	.	.	.	.
B/Malaysia/U3261/2013_2013-12-20	.	.	.	.	.	.	R	.	.	.	.	.	.	.	.	.	V	.	.	.	.	.	.	.	.	.	.	.	.	.	.	.	.	.
540749_B/MALAYSIA/1/2014_2014-01-05	.	.	.	.	.	.	R	.	.	.	.	.	.	T	.	.	.	.	.	.	.	.	.	.	.	.	.	.	.	.	.	.	.	T
B/Malaysia/U3349/2014_2014-01-10	.	.	.	.	.	.	R	.	.	.	.	.	.	.	.	.	.	.	.	.	.	.	.	.	.	.	.	.	.	.	.	.	.	.
B/Malaysia/U3419/2014_2014-01-24	.	.	.	.	.	.	R	.	.	.	.	.	.	.	.	.	.	.	.	.	.	.	.	.	.	.	.	.	.	.	.	.	.	.
B/Malaysia/U3490/2014_2014-02-10	.	.	.	.	.	.	R	.	.	.	.	.	.	.	.	.	.	.	.	.	.	.	.	.	.	.	.	.	.	.	.	.	.	.
B/Malaysia/U3523/2014_2014-02-17	.	.	.	.	.	.	R	T	.	.	.	.	.	.	.	.	.	.	.	.	.	.	.	.	.	.	.	.	.	.	.	.	.	.
B/Malaysia/U3561/2014_2014-02-26	.	.	.	.	M	.	R	.	.	.	.	S	.	.	.	.	.	.	.	.	.	.	.	.	.	.	.	.	.	.	.	.	.	.
B/Malaysia/U3867/2014_2014-05-05	.	.	.	.	.	.	R	.	.	.	F	.	.	.	.	.	.	.	.	.	.	.	.	.	.	.	.	N	.	.	.	.	.	.
B/Malaysia/U963/2012_2012-08-06	.	.	.	.	.	.	R	.	.	.	.	.	.	.	.	.	.	.	T	.	.	.	H	.	.	.	.	.	.	.	.	.	.	.
B/Malaysia/U2335/2013_2013-04-24	.	.	.	.	.	.	R	.	.	.	.	.	.	.	.	.	.	.	.	.	.	.	H	.	.	.	.	.	.	.	.	.	.	.
B/Malaysia/U3340/2014_2014-01-08	.	.	.	.	.	.	R	.	.	.	.	.	.	.	.	.	.	.	.	.	.	.	H	.	.	.	.	.	L	.	.	.	.	.
541275_B/MALAYSIA/3/2014_2014-02-09	.	.	.	.	.	.	R	.	.	.	.	.	.	.	.	.	.	.	.	.	T	.	H	.	.	.	.	.	.	.	.	.	.	.
B/Malaysia/U3519/2014_2014-02-17	.	.	.	.	.	.	R	.	.	.	.	.	.	.	.	.	.	.	.	.	.	.	H	.	.	.	.	.	L	.	.	.	.	.
B/Malaysia/U3601/2014_2014-03-07	.	.	.	.	.	.	R	.	.	.	.	.	.	.	.	.	.	.	.	.	.	.	H	K	.	.	.	.	.	N	.	.	.	.
B/Malaysia/U3804/2014_2014-04-21	.	.	.	.	.	.	.	.	.	.	.	.	.	.	.	.	.	.	.	.	.	.	H	.	.	.	.	.	.	.	.	.	.	.

Grey highlight indicates major clade-defining amino acid substitutions. Substitutions are compared with B/Massachusetts/02/2012 vaccine strain.
