# Supplementary material for: Epidemiological and Evolutionary Dynamics of Influenza B Viruses in Malaysia, 2012-2014
Source: PLoS One. 2015 Aug 27;10(8):e0136254. doi: 10.1371/journal.pone.0136254 (PMC4552379; doi:10.1371/journal.pone.0136254)
Supplement: S9 Table — Grey highlight indicates major signature amino acid substitutions. Substitutions are compared with B/Wisconsin/01/2010 vaccine strain. (PDF) [file pone.0136254.s015.pdf]

S9 Table. Amino acid substitutions found on the NA protein for all Malaysian Yamagata Clade 3 viruses (n=69).

Amino Acid Position	8	13	27	36	40	41	45	46	50	55	60	62	68	71	72	73	75	76	106	109	221	222	223	244	260	261	268	283	290	295	320	334	340	342	343	357	371	395	399	403	416	427	439	464
N2 Numbering System	12	17	30		40	41	44	45	49	54	59	61	67	70	71	72	74	75		111	222	223	224	245	261	262	269	283	290	295	320	334	340	341	342		369	391	395	399	414	425	440	467
B/Wisconsin/01/2010_2010-02-20	T	L	S	I	F	S	I	T	M	A	V	A	T	V	T	L	L	P	T	N	I	L	R	P	R	I	T	S	A	S	E	T	N	D	K	M	K	A	V	M	K	I	H	M
B/Stockholm/12/2011_2011-03-28	.	.	.	.	.	.	.	.	.	.	.	.	.	.	.	P	.	.	.	.	.	.	.	.	.	.	.	.	.	.	.	.	.	.	E	.	.	.	.	.	.	.	.	.
B/Phuket/3073/2013_2013-11-21	.	.	.	.	.	.	V	.	.	.	.	.	.	.	.	P	.	.	.	.	.	.	.	.	.	.	.	.	.	.	K	.	D	.	E	.	.	.	.	.	.	.	.	.
B/Malaysia/U69/2012_2012-03-07	.	I	.	.	.	.	.	.	.	.	.	.	.	.	.	.	.	.	.	.	.	.	.	.	.	.	.	.	.	.	.	.	.	.	.	.	.	.	.	.	.	.	.	.
B/Malaysia/U116/2012_2012-03-14	.	.	.	.	.	.	.	.	.	.	.	.	.	.	.	.	.	.	.	.	.	.	.	.	.	.	.	.	.	.	.	.	.	.	.	.	.	V	.	.	.	.	.	.
B/Malaysia/U123/2012_2012-03-14	.	.	.	.	.	.	.	.	.	.	.	.	.	.	.	.	.	.	.	.	.	.	.	.	.	.	.	.	.	.	.	.	.	.	.	.	.	.	.	.	.	.	.	.
B/Malaysia/U140/2012_2012-03-19	.	.	.	.	.	.	.	.	.	.	.	.	.	.	.	.	.	.	.	.	.	.	.	.	.	.	.	.	.	.	.	.	.	.	.	.	.	.	.	.	.	.	.	.
B/Malaysia/U287/2012_2012-04-13	.	.	.	.	.	.	.	.	.	.	.	.	.	.	.	.	.	.	.	.	.	.	.	.	.	.	.	.	.	.	.	.	.	.	.	.	.	.	.	.	.	.	.	.
B/Malaysia/U432/2012_2012-05-04	.	.	L	.	.	.	.	.	.	.	.	.	.	.	.	.	.	.	.	.	.	.	.	.	.	.	.	.	.	.	.	.	.	.	.	.	.	.	.	.	.	.	.	.
B/Malaysia/U579/2012_2012-05-30	.	.	.	.	.	.	.	.	.	.	.	.	.	.	.	.	.	.	.	.	.	.	.	.	.	.	.	.	.	.	.	.	.	.	.	.	.	.	.	.	.	.	.	.
B/Malaysia/U699/2012_2012-06-18	.	.	.	.	.	.	.	.	.	.	.	.	.	.	.	.	.	.	.	.	.	.	.	.	.	.	.	.	.	.	.	.	.	.	.	.	.	.	.	.	.	.	.	.
450337_B/MALAYSIA/878/2012_2012-07-06	.	.	.	.	.	.	.	.	.	.	.	.	.	.	.	.	.	.	.	.	.	.	.	.	.	V	.	.	.	.	.	.	.	.	.	.	.	.	.	.	R	.	.	.
B/Malaysia/U951/2012_2012-08-03	.	.	.	.	.	.	.	.	.	.	.	.	.	.	.	.	.	.	.	.	.	.	.	.	.	.	.	.	.	.	.	.	.	.	.	.	.	.	.	.	.	.	.	.
B/Malaysia/U960/2012_2012-08-06	.	.	.	.	.	.	.	.	.	.	.	.	.	.	.	.	.	.	.	.	.	.	.	.	.	.	.	.	.	.	.	.	.	.	.	.	.	.	.	.	.	.	.	.
B/Malaysia/U1264/2012_2012-10-05	M	.	.	.	.	.	.	.	.	.	.	.	.	.	A	.	.	.	.	.	.	.	.	.	.	.	.	.	.	.	.	.	D	.	.	.	.	.	.	.	.	.	.	.
B/Malaysia/U1338/2012_2012-10-19	.	.	.	M	.	.	.	.	.	.	.	.	.	.	.	.	.	.	.	.	.	.	.	.	.	.	.	.	.	.	.	.	.	.	.	.	.	.	.	L	.	.	.	.
B/Malaysia/U1879/2013_2013-01-21	.	.	.	.	.	.	.	.	.	.	.	.	.	.	.	.	.	.	.	.	.	.	.	.	.	.	.	.	.	.	.	.	.	.	.	.	.	.	.	.	.	.	.	.
B/Malaysia/U1936/2013_2013-02-04	.	.	.	.	.	.	.	.	.	.	.	.	.	M	.	.	.	.	.	.	.	.	.	.	.	.	.	.	.	.	.	.	.	.	.	.	.	.	.	.	.	.	.	.
B/Malaysia/U1995/2013_2013-02-20	.	.	.	.	.	.	.	.	.	.	.	.	.	M	.	.	.	.	.	.	.	.	.	.	.	.	I	.	.	.	.	.	.	.	.	.	.	.	.	.	.	.	.	.
B/Malaysia/U2002/2013_2013-02-20	.	.	.	.	.	.	.	.	I	.	.	.	.	.	.	.	.	.	.	.	.	.	.	.	.	.	.	.	.	.	.	.	.	.	.	.	.	.	.	.	.	.	.	.
B/Malaysia/U2094/2013_2013-03-06	.	.	.	.	.	.	.	.	I	.	.	.	.	.	.	.	.	.	.	.	.	.	.	.	.	.	.	.	.	.	.	.	.	.	.	.	.	.	.	.	.	.	.	.
477625_B/MALAYSIA/16/2013_2013-03-21	.	.	.	.	.	.	.	.	I	.	.	.	.	.	.	.	.	.	.	.	.	.	.	.	.	.	.	.	.	.	.	.	.	.	.	.	.	.	.	.	.	.	.	.
B/Malaysia/U2547/2013_2013-06-24	.	.	.	.	.	.	.	.	I	.	I	.	.	.	.	.	.	.	.	.	.	.	.	.	.	.	.	.	.	.	.	.	.	.	.	.	.	.	.	.	.	.	.	.
466158_B/MALAYSIA/4/2013_2013-02-27	.	.	.	.	.	.	.	.	.	.	.	.	.	.	.	.	.	.	.	.	N	P	K	.	.	.	.	.	.	.	.	.	.	.	.	.	.	.	.	.	.	.	.	.
B/Malaysia/U2080/2013_2013-03-06	.	.	.	.	.	.	.	.	.	.	.	.	.	.	.	.	.	.	.	.	.	.	.	.	.	V	.	.	.	.	.	.	.	.	.	.	.	.	.	.	R	.	.	.
B/Malaysia/U2111/2013_2013-03-11	.	.	.	.	.	.	.	.	.	.	.	.	.	.	.	.	.	.	.	.	.	.	.	.	.	.	.	.	.	.	.	.	.	.	.	.	.	.	.	.	.	.	.	.
B/Malaysia/U2120/2013_2013-03-11	.	.	.	.	.	.	.	.	.	.	.	.	.	.	.	.	.	.	.	.	.	.	.	.	.	.	.	.	T	.	.	.	.	.	.	.	.	.	.	.	.	.	.	.
B/Malaysia/U2154/2013_2013-03-18	.	.	.	.	.	.	.	.	.	.	.	.	.	.	.	.	.	.	.	.	.	.	.	.	.	.	.	.	.	.	.	.	.	.	.	.	.	.	.	.	.	.	.	.
B/Malaysia/U2179/2013_2013-03-22	.	.	.	M	.	L	.	.	.	.	.	.	.	.	.	F	.	.	.	.	.	.	.	.	.	.	.	.	.	.	.	.	.	.	.	.	.	.	.	.	R	.	.	.
B/Malaysia/U2234/2013_2013-04-01	.	.	.	.	.	.	.	.	.	.	.	.	.	.	.	.	.	.	.	.	.	.	.	.	.	.	.	.	.	.	.	.	.	.	.	.	.	.	.	.	.	.	.	.
B/Malaysia/U2265/2013_2013-04-08	.	.	.	.	.	.	.	.	.	.	.	.	.	.	.	.	.	.	.	.	.	.	.	.	.	.	.	.	.	.	.	.	.	.	.	.	.	.	.	.	.	.	.	.
B/Malaysia/U2447/2013_2013-05-27	.	.	.	.	.	.	.	.	.	.	.	.	.	.	.	.	.	.	.	.	.	.	.	.	.	.	.	.	.	.	.	.	.	.	.	.	.	.	.	.	.	.	.	.
B/Malaysia/U159/2012_2012-03-21	.	.	.	.	.	.	.	N	.	.	.	.	.	.	A	.	.	.	.	.	.	.	.	.	.	.	.	.	.	.	.	.	.	.	.	.	.	.	.	.	.	.	.	.
B/Malaysia/U169/2012_2012-03-23	.	.	.	.	.	.	.	.	.	.	.	.	.	.	.	P	.	.	.	.	.	.	.	.	.	.	.	.	.	.	.	.	.	.	E	.	.	.	.	.	.	.	.	.
B/Malaysia/U182/2012_2012-03-26	.	.	.	.	.	.	.	.	.	.	.	.	.	.	.	P	.	.	.	.	.	.	.	.	.	.	.	.	.	.	.	.	.	.	E	.	.	.	.	.	.	.	.	.
B/Malaysia/U210/2012_2012-03-28	.	.	.	.	.	.	.	.	.	.	.	.	.	.	.	P	.	.	A	.	.	.	.	.	.	.	.	.	.	.	.	.	.	.	E	.	.	.	.	.	.	.	.	.
B/Malaysia/U316/2012_2012-04-16	.	.	.	M	.	.	.	.	.	S	.	.	.	.	.	P	.	S	.	.	.	.	.	S	.	.	.	.	.	.	.	.	.	.	E	.	.	.	.	.	.	.	.	.
B/Malaysia/U1065/2012_2012-08-29	.	.	.	.	.	.	.	.	.	.	.	.	.	.	.	P	.	.	.	.	.	.	.	.	.	.	.	.	.	.	.	.	.	.	E	.	.	.	.	.	.	.	.	.
B/Malaysia/U1580/2012_2012-11-30	.	.	.	.	.	.	.	.	.	.	.	.	.	.	.	P	.	.	.	.	.	.	.	S	.	.	.	.	.	.	.	.	.	.	E	.	.	.	.	.	.	.	.	.
B/Malaysia/U2046/2013_2013-02-27	.	.	.	.	.	.	.	.	.	.	.	.	.	.	.	P	.	.	.	.	.	.	.	.	.	.	.	.	.	.	.	.	.	N	E	.	.	T	.	.	.	.	.	.
B/Malaysia/U2462/2013_2013-05-31	.	.	.	.	.	.	V	.	.	.	.	.	.	.	.	P	.	.	.	.	.	.	.	.	.	.	.	.	.	.	K	.	D	.	E	.	.	.	.	.	.	.	.	.
B/Malaysia/U2555/2013_2013-06-26	.	.	.	.	.	.	V	.	.	.	.	.	.	.	.	P	.	.	.	K	.	.	.	.	.	.	.	.	.	.	K	.	D	.	E	.	.	.	.	.	.	.	.	.
B/Malaysia/U3225/2013_2013-12-13	.	.	.	.	.	.	V	.	.	.	.	.	.	.	.	P	.	.	.	.	.	.	.	.	.	.	.	.	.	.	K	.	D	.	E	.	.	.	.	.	.	.	.	.
B/Malaysia/U3328/2014_2014-01-06	.	.	.	.	.	.	V	.	.	.	.	.	.	.	.	P	.	.	.	.	.	.	.	.	.	.	.	.	.	.	K	.	D	.	E	.	.	.	.	.	.	.	.	.
B/Malaysia/U3404/2014_2014-01-20	.	.	.	.	.	.	V	.	.	.	.	.	.	.	.	P	.	.	.	.	.	.	.	.	.	.	.	.	.	.	K	.	D	.	E	.	.	.	.	.	.	.	.	.
B/Malaysia/U3411/2014_2014-01-24	.	.	.	.	.	.	V	.	.	.	.	.	.	.	.	P	.	.	.	.	.	.	.	.	.	.	.	.	.	.	K	.	D	.	E	.	.	.	.	.	.	.	.	.
B/Malaysia/U3435/2014_2014-01-29	.	.	.	.	.	.	V	I	.	.	.	.	.	.	.	P	.	.	.	.	.	.	.	.	.	.	.	.	.	.	K	.	D	.	E	.	.	.	.	.	.	.	.	.
B/Malaysia/U3497/2014_2014-02-12	.	.	.	.	.	.	V	.	.	.	.	T	.	.	.	P	.	.	.	.	.	.	.	.	.	.	.	.	.	.	K	.	D	.	E	.	.	.	.	I	.	.	.	.
541231_B/MALAYSIA/7/2014_2014-02-20	.	.	.	.	.	.	V	.	.	.	.	.	.	.	.	P	.	.	.	.	.	.	.	.	.	.	.	.	.	.	K	.	D	.	E	.	.	.	.	.	.	.	.	.
B/Malaysia/U3626/2014_2014-03-12	.	.	.	.	.	.	V	.	.	.	.	T	.	.	.	P	.	.	.	.	.	.	.	.	.	.	.	.	.	.	K	.	D	.	E	.	.	.	.	.	.	.	.	.
B/Malaysia/U3630/2014_2014-03-12	.	.	.	.	.	.	V	.	.	.	.	.	.	.	.	P	.	.	.	.	.	.	.	.	.	.	.	.	.	.	K	.	D	.	E	.	.	.	.	.	.	.	.	.
B/Malaysia/U3636/2014_2014-03-12	.	.	.	.	.	.	V	.	.	.	.	.	.	.	.	P	.	.	.	.	.	.	.	.	.	.	.	.	.	.	K	.	D	.	E	.	.	.	.	.	.	.	.	.
B/Malaysia/U3655/2014_2014-03-17	.	.	.	.	.	.	V	.	.	.	.	.	.	.	.	P	F	.	.	.	.	.	.	.	.	.	.	.	.	.	K	.	D	.	E	.	.	.	.	.	.	.	.	.
B/Malaysia/U3656/2014_2014-03-17	.	.	.	.	.	.	V	.	.	.	.	.	.	.	.	P	.	.	.	.	.	.	.	.	.	.	.	.	.	.	K	.	D	.	E	.	.	.	.	.	.	.	.	.
B/Malaysia/U3685/2014_2014-03-21	.	.	.	.	L	.	V	.	.	.	.	.	.	.	.	P	.	.	.	.	.	.	.	.	.	.	.	.	.	N	K	.	D	.	E	.	.	.	A	.	.	.	.	.
541281_B/MALAYSIA/8/2014_2014-03-26	.	.	.	.	.	.	V	.	.	.	.	.	.	.	.	P	.	.	.	.	.	.	.	.	.	.	.	.	.	.	K	.	D	.	E	.	.	.	.	.	.	.	.	.
B/Malaysia/U3802/2014_2014-04-21	.	.	.	.	.	.	V	.	.	.	.	.	.	.	.	P	F	.	.	.	.	.	.	.	.	.	.	.	.	.	K	.	D	.	E	.	.	.	.	.	.	.	.	.
B/Malaysia/U3805/2014_2014-04-23	.	.	.	.	.	.	V	.	.	.	.	.	.	.	.	P	.	.	.	.	.	.	.	.	.	.	.	.	.	.	K	.	D	.	E	T	.	.	.	.	.	.	.	.
B/Malaysia/U3901/2014_2014-05-16	.	.	.	.	.	.	V	.	.	.	.	T	.	.	.	P	.	.	.	.	.	.	.	.	.	.	.	.	.	.	K	.	D	.	E	.	.	.	.	.	.	.	.	.
551266_B/MALAYSIA/10/2014_2014-06-11	.	.	.	.	.	.	V	.	.	.	.	.	.	.	.	P	.	.	.	.	.	.	.	.	.	.	.	N	.	.	K	.	D	.	E	.	.	.	.	.	.	.	.	.
B/Malaysia/U3224/2013_2013-12-13	.	.	.	.	.	.	V	.	.	.	A	.	.	.	.	P	.	.	.	.	.	.	.	.	Q	.	.	.	.	.	K	.	D	.	E	.	.	.	.	.	.	.	.	I
B/Malaysia/U3226/2013_2013-12-13	.	.	.	.	.	.	V	.	.	.	A	.	.	.	.	P	.	.	.	.	.	.	.	.	.	.	.	.	.	.	K	.	D	.	E	.	.	.	.	.	.	.	.	.
B/Malaysia/U3277/2013_2013-12-23	.	.	.	.	.	.	V	.	.	.	A	.	.	.	.	P	.	.	.	.	.	.	.	.	.	.	.	.	.	.	K	I	D	.	E	.	.	.	.	.	.	.	.	I
B/Malaysia/U3288/2013_2013-12-27	.	.	.	.	.	.	V	.	.	.	A	.	.	.	.	P	.	.	.	.	.	.	.	.	.	.	.	.	.	.	K	.	D	.	E	.	.	.	.	.	.	.	Q	.
B/Malaysia/U3488/2014_2014-02-10	.	.	.	.	.	.	V	.	.	.	A	.	.	.	.	P	.	.	.	.	.	.	.	.	.	.	.	.	.	.	K	.	D	.	E	.	.	.	.	.	.	.	.	.
B/Malaysia/U3734/2014_2014-04-02	.	.	.	.	.	.	V	.	.	.	A	.	.	.	.	P	.	.	.	.	.	.	.	.	.	.	I	.	.	.	K	.	D	.	E	.	E	.	.	.	.	.	.	I
B/Malaysia/U3876/2014_2014-05-09	.	.	.	.	.	.	V	.	.	.	A	.	.	.	.	P	.	.	.	.	.	.	.	.	.	.	.	.	.	.	K	.	D	.	E	.	.	.	.	.	.	.	.	.
B/Malaysia/U3663/2014_2014-03-17	.	.	.	.	.	.	V	.	.	.	.	.	.	.	.	P	.	.	.	.	.	.	.	.	.	.	.	.	.	.	K	.	D	N	E	.	.	.	.	.	.	.	.	.
B/Malaysia/U3679/2014_2014-03-21	.	.	.	.	.	.	V	.	.	.	.	.	A	.	.	P	.	.	.	.	.	.	.	.	.	.	.	.	.	.	K	.	D	N	E	.	.	.	.	.	.	V	.	.
B/Malaysia/U3794/2014_2014-04-21	.	.	.	.	.	.	V	.	.	.	.	.	.	.	.	P	.	.	.	.	.	.	.	.	.	.	.	.	.	.	K	.	D	N	E	.	.	.	.	.	.	.	.	.
B/Malaysia/U3895/2014_2014-05-14	.	.	.	.	.	.	V	.	.	.	.	.	.	.	.	P	.	.	.	.	.	.	.	.	.	.	.	.	.	.	K	.	D	N	E	.	.	.	.	.	.	.	.	.
B/Malaysia/U2214/2013_2013-03-29	.	.	.	.	.	.	.	.	.	.	.	.	.	.	.	P	.	.	.	.	.	.	.	.	.	.	.	.	.	.	.	.	.	N	E	.	.	T	.	.	.	.	.	.

Grey highlight indicates major clade-defining amino acid substitutions. Substitutions are compared with B/Wisconsin/01/2010 vaccine strain.
