# Supplementary material for: Epidemiological and Evolutionary Dynamics of Influenza B Viruses in Malaysia, 2012-2014
Source: PLoS One. 2015 Aug 27;10(8):e0136254. doi: 10.1371/journal.pone.0136254 (PMC4552379; doi:10.1371/journal.pone.0136254)

NA Phylogeny - NJ Method (Yamagata Clade 3 Viruses)

Malaysian Yamagata Clade 3 viruses

WHO Reference strains

WHO Candidate vaccine strains

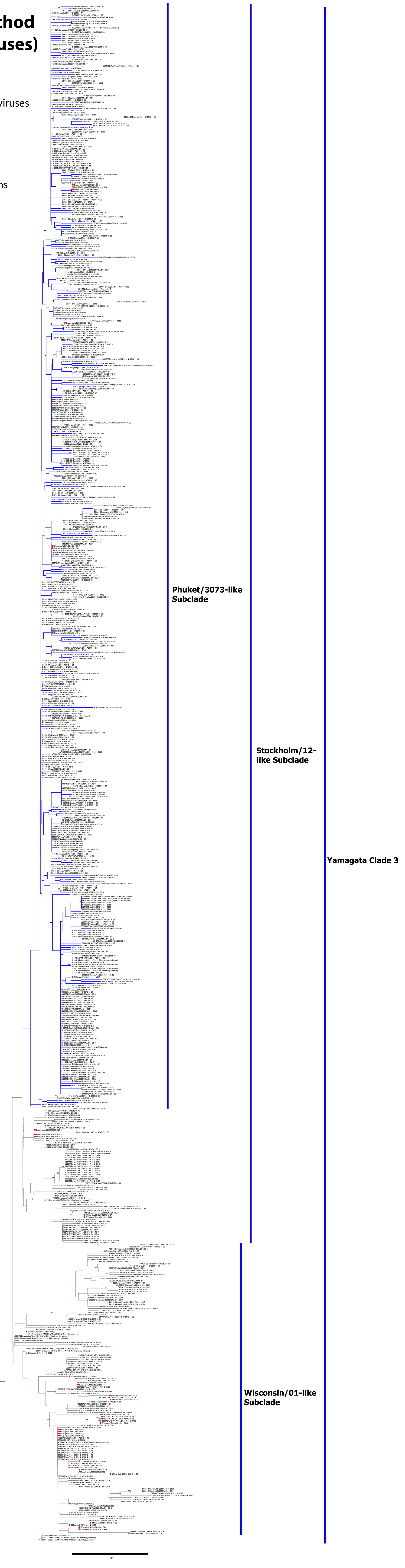

Supplement: S4 Fig — (PDF) [file pone.0136254.s004.pdf]
