# Supplementary material for: Epidemiological and Evolutionary Dynamics of Influenza B Viruses in Malaysia, 2012-2014
Source: PLoS One. 2015 Aug 27;10(8):e0136254. doi: 10.1371/journal.pone.0136254 (PMC4552379; doi:10.1371/journal.pone.0136254)

## HA Phylogeny - MCC Tree
(Phuket/3073-like subclade)

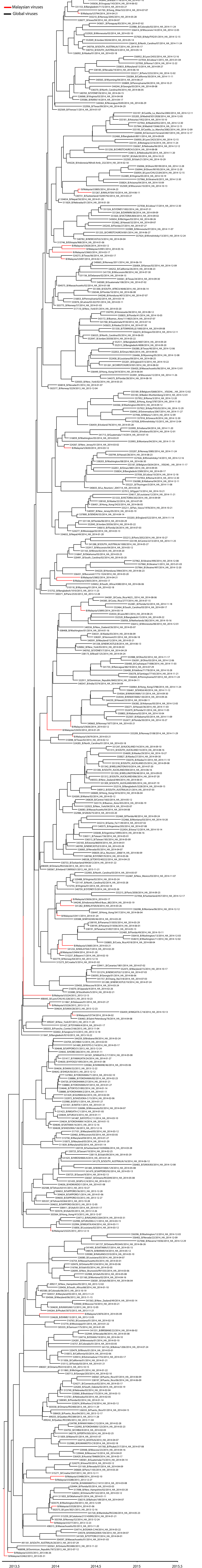

Supplement: S5 Fig — (PDF) [file pone.0136254.s005.pdf]
