# Supplementary material for: Epidemiological and Evolutionary Dynamics of Influenza B Viruses in Malaysia, 2012-2014
Source: PLoS One. 2015 Aug 27;10(8):e0136254. doi: 10.1371/journal.pone.0136254 (PMC4552379; doi:10.1371/journal.pone.0136254)
Supplement: S6 Fig — (PDF) [file pone.0136254.s006.pdf]

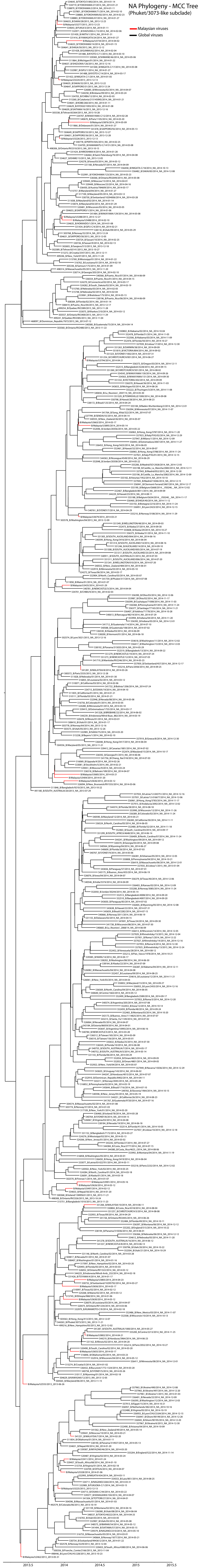
NA Phylogeny - MCC Tree
(Phuket/3073-like subclade)
Malaysian viruses
Global viruses
2013.5
2014
2014.5
2015
2015.5
